# Ecotype‐specific phenolic acid accumulation and root softness in *Salvia miltiorrhiza* are driven by environmental and genetic factors

**DOI:** 10.1111/pbi.70048

**Published:** 2025-03-19

**Authors:** Haomiao Yu, Jinqiu Liao, Yuanyuan Jiang, Mingzhi Zhong, Shan Tao, Songyue Chai, Long Wang, Li Lin, Ruiwu Yang, Xuexue Deng, Yunsong Zhang, Xiang Pu, Moyang Liu, Li Zhang

**Affiliations:** ^1^ College of Science Sichuan Agricultural University Ya'an China; ^2^ College of Life Science Sichuan Agricultural University Ya'an China; ^3^ Industrial Crop Research Institute Sichuan Academy of Agricultural Sciences Chengdu China; ^4^ Joint Center for Single Cell Biology, Department of Plant Sciences, School of Agriculture and Biology Shanghai Jiao Tong University Shanghai China

**Keywords:** evolution, genetic and environmental factors, phenolic acid metabolism, multi‐omics analysis, *S. miltiorrhiza*

## Abstract

*Salvia miltiorrhiza* Bunge, a renowned medicinal herb in traditional Chinese medicine, displays distinctive root texture and high phenolic acid content, traits influenced by genetic and environmental factors. However, the underlying regulatory networks remain unclear. Here, we performed multi‐omics analyses on ecotypes from four major Chinese regions, focusing on environmental impacts on root structure, phenolic acid accumulation and lignin composition. Lower temperatures and increased UV‐B radiation were associated with elevated rosmarinic acid (RA) and salvianolic acid B (SAB) levels, particularly in the Sichuan ecotype. Structural models indicated that the radial arrangement of xylem conduits contributes to greater root hardness. Genomic assembly and comparative analysis of the Sichuan ecotype revealed a unique phenolic acid metabolism gene cluster, including *SmWRKY40*, a WRKY transcription factor essential for RA and SAB biosynthesis. Overexpression of *SmWRKY40* enhanced phenolic acid levels and lignin content, whereas its knockout reduced root hardness. Integrating high‐throughput (DNA affinity purification sequencing) and point‐to‐point (Yeast One‐Hybrid, Dual‐Luciferase and Electrophoretic Mobility Shift Assay) protein‐DNA interaction detection platform further identified *SmWRKY40* binding sites across ecotypes, revealing specific regulatory networks. Our findings provide insights into the molecular basis of root texture and bioactive compound accumulation, advancing breeding strategies for quality improvement in *S. miltiorrhiza*.

## Introduction

In recent years, *Salvia miltiorrhiza* Bunge, commonly known as Danshen, has garnered extensive interest in pharmaceutical research due to its medicinal properties, particularly in treating cardiovascular diseases and other ailments (Li *et al*., 2018, 2020b; Shi *et al*., 2019; Xiao *et al*., 2020). This perennial herb has long been valued in traditional medicine and serves as a rich source of bioactive compounds, especially phenolic acids like rosmarinic acid (RA) and salvianolic acid B (SAB), which contribute significantly to its therapeutic efficacy (Yan, 2016; Zhou *et al*., 2018). The phenolic acids accumulate primarily in the roots, where their synthesis and accumulation are subject to complex regulatory mechanisms involving genetic and environmental influences (Liang *et al*., 2020). Notably, these variations in phenolic acid levels often correlate with changes in root morphology and mechanical properties, including root softness and lignification levels (Chen *et al*., 2023; Yajun *et al*., 2013). These traits not only impact the quality and consistency of *S. miltiorrhiza*'s medicinal products but also highlight the plant's adaptive responses to the diverse regional environments across China, where it is widely cultivated (Qin *et al*., 2021; Saini *et al*., 2019). As such, elucidating the regulatory networks underlying these traits is essential for optimizing cultivation practices to enhance the quality and consistency of bioactive compounds in *S. miltiorrhiza*.

The biosynthesis of phenolic acids in *S. miltiorrhiza* is a highly regulated process shaped by intricate interactions between genetic and environmental factors (Guo *et al*., 2022; Shi *et al*., 2024). At the genetic level, specific gene clusters have been identified as central to phenolic acid biosynthesis, with transcription factors (Shi *et al*., 2024; Shuangqian *et al*., 2021; Wang *et al*., 2023; Zhang *et al*., 2024a), such as the WRKY (Saha *et al*., 2023) and MYB (Ma *et al*., 2024) families, playing critical regulatory roles in modulating gene expression. These transcription factors are often sensitive to external stimuli and can act as molecular hubs that integrate environmental signals into genetic responses. Additionally, various environmental factors—such as ultraviolet (UV) radiation (Yin *et al*., 2020), temperature fluctuations (Li *et al*., 2020a), heavy metal stress (Fan *et al*., 2023) and soil mineral composition (Lv *et al*., 2024)—have been shown to significantly influence the phenolic acid content in *S. miltiorrhiza* roots. For example, studies indicate that UV‐B exposure and cooler temperatures can stimulate phenolic acid production, potentially due to stress‐induced regulatory pathways that activate biosynthetic genes (Xiaojian Yin *et al*., 2020). Yet, despite these advances, the extent to which genetic and environmental factors interact to shape root structure, lignin distribution and consequently the root's medicinal and mechanical properties, remains largely unexplored.

Previous studies on *S. miltiorrhiza* have primarily focused on identifying individual genes or specific environmental factors that influence phenolic acid accumulation and root characteristics (Cao *et al*., 2024; Deng *et al*., 2020; Huang *et al*., 2018; Jia *et al*., 2024; Li *et al*., 2023; Liu *et al*., 2022; Lv *et al*., 2024; Zhou *et al*., 2021). However, these studies often fall short in capturing the broader, interconnected network of interactions that govern both phenolic acid biosynthesis and root structure, particularly with regard to root softness and lignification patterns. Furthermore, the genetic basis of root softness remains poorly understood, and there is limited knowledge about how lignin distribution patterns vary among ecotypes of *S. miltiorrhiza*, which exhibit distinct phenolic profiles and morphological traits across different growing environments (Chen *et al*., 2023; Yajun *et al*., 2013; Yu *et al*., 2023; Zhang *et al*., 2024b). This gap in understanding poses challenges to developing high‐quality *S. miltiorrhiza* cultivars that combine enhanced phenolic acid levels with optimal root characteristics suited for medicinal use.

Therefore, our study employs a multi‐omics approach that integrates genomic, transcriptomic, metabolomic and interactive‐omics data to unravel the regulatory networks underlying phenolic acid accumulation and root texture variation across different *S. miltiorrhiza* ecotypes. By examining these data across various ecotypes, we aim to identify key environmental factors, transcriptional regulators and gene clusters that collectively influence these critical traits. This comprehensive approach not only reveals potential molecular markers that can guide breeding strategies for high‐quality *S. miltiorrhiza* cultivars but also offers insights into the adaptive evolution of *S. miltiorrhiza* in response to diverse environmental conditions in its natural habitats. By characterizing the complex gene–environment interactions that drive phenolic acid biosynthesis and root morphology, our study contributes to a deeper understanding of the genetic and environmental basis for the medicinal qualities of *S. miltiorrhiza*, thereby paving the way for advancements in both agricultural and medicinal applications of this valuable herb.

## Results

### Regional adaptation and structural traits in *Salvia miltiorrhiza* influence phenolic acid accumulation and root softness

**Figure 1:**
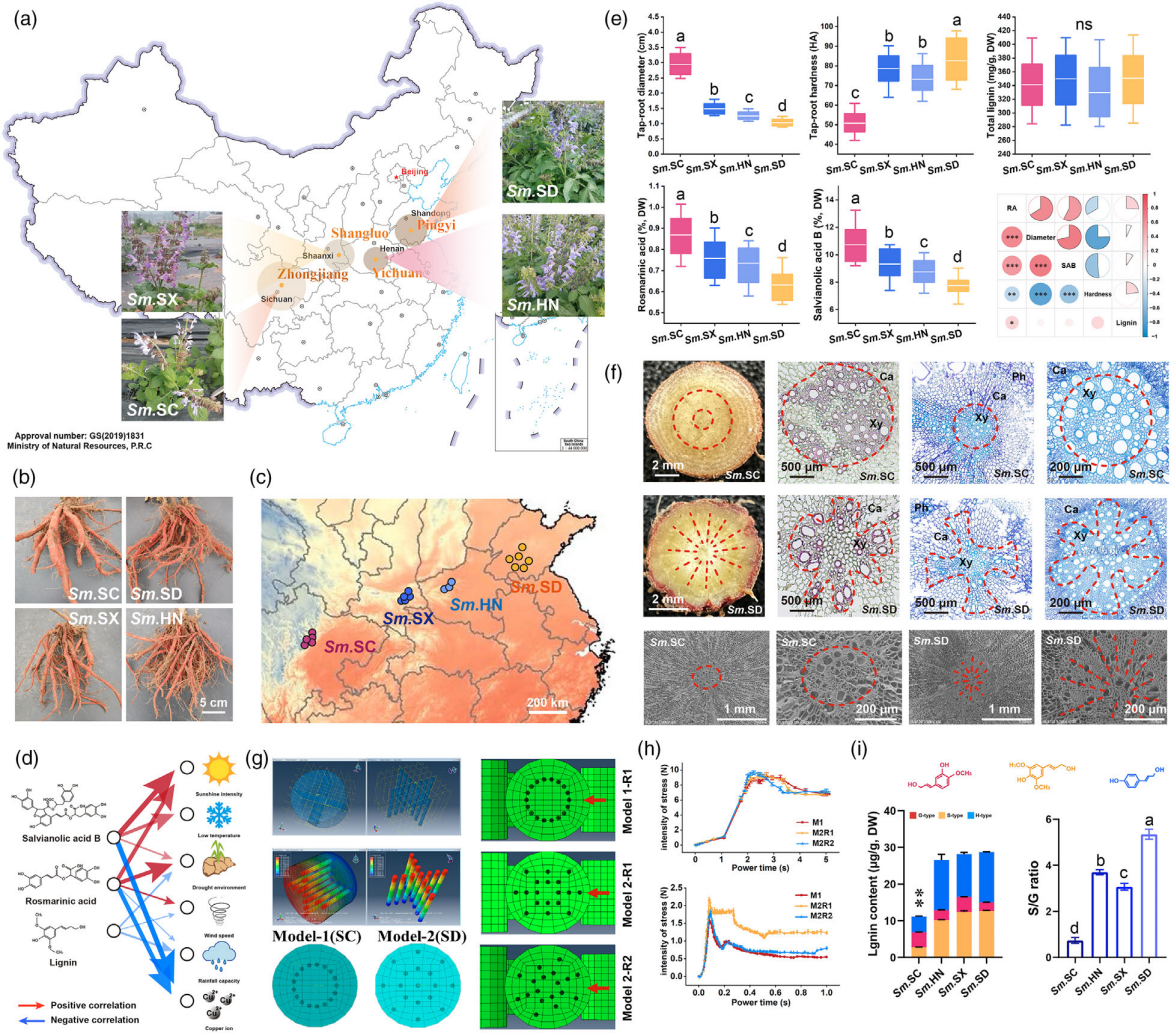
The Component accumulation, morphology, anatomy and environmental correlation of roots of *S. miltiorrhiza* with different ecotypes. (a) Morphological characteristics and geographical distribution of *S. miltiorrhiza* in Sichuan, Shaanxi, Henan and Shandong provinces. (b) Morphological characteristics of mature root strips of *S. miltiorrhiza* with different ecotypes. (c) Daily average temperature heatmap of different *S. miltiorrhiza*‐producing regions in China during the root maturation period. (d) Correlation analysis between rosmarinic acid, salvianolic acid B, lignin content and environmental factors. (e) Root diameter, hardness, total lignin content, rosmarinic acid content, salvianolic acid B content. and correlation analysis between phenotype and components. Data show the arithmetic mean ± SD from 3 biological replicates (*Sm.SC*, *n* = 27; *Sm.SX*, *n* = 10; *Sm.HN*, *n* = 20; *Sm.SD*, *n* = 23; plants each). Different letters indicate significant differences at *P* < 0.05 (one‐way ANOVA, Tukey's posttest). (f) Paraffin sectioning and scanning electron microscopy imaging of mature roots of ecological type *S. miltiorrhiza* in Sichuan and Shandong. The red lines highlight the arrangement of xylem cells, and the Black letters mark the areas of the xylem (Xy), cuticular (Ca) and phloem (Ph). (g) Mechanical simulation analysis of bending deformation and extrusion deformation of root strips with different lignocellulosic arrangements using 3D modelling. A total of 2 rounds (R1 and R2) of force application direction for 2 models (M1 and M2) were analysed. (h) The dynamic changes in action time and stress intensity during bending deformation and extrusion deformation processes were recorded separately. (i) Comparison of different lignin monomers and S/G ratio in the root of Sichuan and Shandong *S. miltiorrhiza*.

The quality traits of traditional Chinese medicinal (TCM) herbs are generally thought to be influenced by both genetic and environmental factors, a hypothesis widely accepted as the basis for their authenticity. To evaluate this hypothesis, we performed a systematic analysis of germplasm resources from four major *S. miltiorrhiza*‐producing regions in China: Zhongjiang, Sichuan (*Sm.SC*); Shangluo, Shaanxi (*Sm.SX*); Yincheng, Henan (*Sm.HN*); and Mengyin, Shandong (*Sm.SD*) (Figure 1a, Table S1). The primary roots of *Sm.SC* exhibited a notably thicker structure, with finer side roots and softer root textures than other ecotypes (Figure 1b). These regions span from the eastern coast to the central plains and from the northwest to the southwest of China. The average daily temperature during the root enlargement phase showed a declining trend with elevation, varying distinctly across these regions without overlap (Figure 1c).

We further assessed the influence of 15 environmental factors, including ultraviolet radiation, sunshine duration and wind speed, on the accumulation of active medicinal compounds in *S. miltiorrhiza* roots (Figure S1a–c, Table S2). Random sampling of air temperature, UV‐B radiation intensity and soil copper (Cu) content in the four regions revealed consistent trends (Figure S1d–f). Lower temperatures and higher UV‐B exposure were associated with elevated levels of rosmarinic acid (RA) and salvianolic acid B (SAB), while elevated Cu concentrations, particularly in the Shandong region (*Sm.SD*), showed a negative correlation with RA and SAB levels (Figure 1d). To further confirm the impact of these environmental factors, we subjected 6‐month‐old *S. miltiorrhiza* seedlings to UV‐B radiation, cold and Cu stress. These treatments significantly inhibited root growth and development (Figure S2a–c). Specifically, UV‐B and cold exposure promoted RA and SAB biosynthesis, whereas Cu stress reduced their accumulation. Lignin content under these stress conditions followed the same trend as RA and SAB (Figure S2d–f).

We then evaluated phenotypic traits, RA, SAB and lignin content across various *S. miltiorrhiza* ecotypes. *Sm.SC* roots exhibited the greatest thickness, softest texture and highest levels of RA and SAB. Interestingly, total lignin content did not show a consistent trend across populations. We observed that RA and SAB levels inversely correlated with root hardness, whereas lignin content correlated positively with root hardness (Figure 1e). Further structural analysis through histological staining and scanning electron microscopy revealed that lignified areas in *Sm.SC* roots displayed a clustered, circular distribution, in contrast to the radial arrangement found in other ecotypes, such as *Sm.SD*, which has lower phenolic acid content and higher hardness (Figure 1f).

To better understand how xylem distribution patterns affect root mechanical strength, we modelled the anatomical structure of *S. miltiorrhiza* roots using two simplified models. A composite material model was developed with high‐hardness materials as the framework and low‐hardness fillers to simulate the mechanical effects of different conduit arrangements (Figure 1g). Stress displacement dynamics were examined under two configurations: circular and radial distributions (Figure S3, Appendix S2). Roots with radially arranged conduits exhibited higher resistance to bending and compression, as shown by stress heatmaps and pressure displacement curves (Figure 1h). Virtual simulation channels further allowed us to monitor root strip deformation under compressive and bending stresses dynamically, presented as video supplements in Appendix S1.

Finally, comparative metabolomic and GC–MS analyses between *Sm.SC* and *Sm.SD* roots indicated a higher concentration of S‐type lignin monomers (syringyl) and derivatives in *Sm.SD* (Figures 1i and S4, Table S3). The three hydroxyl groups on the benzene ring of the S‐type lignin monomer contributed to flexible anisotropy during polymerization (Morreel *et al*., 2004), influencing the radial distribution of lignin within root structures (Meyermans *et al*., 2000). Based on these findings, the significant differences in appearance, environmental adaptability, medicinal quality and mechanical strength, especially the high phenolic acid content and reduced hardness in *Sm.SC* roots prompted the selection of *Sm.SD*, which diverges markedly from *Sm.SC*, for further comparative studies.

### Genetic diversity and selective sweeps in *Salvia miltiorrhiza* reveal tandem duplications linked to lignin and phenolic acid biosynthesis

The blank of high‐quality genetic map of *S. miltiorrhiza* with unique soft roots and high‐phenolic acid traits seriously restricts its quality formation research and genetic improvement. To investigate the genetic influences on the metabolic evolution of *S. miltiorrhiza*, we selected the Sichuan ecotype (Chuandanshen No. 1, CDS‐1) as a representative sample. Using the PacBio platform, we generated 125.47 Gb of genomic data with a coverage depth of 93.84×, resulting in a 603.67 Mb chromosome‐level reference genome (Figure S5, Table S4). Hi‐C‐assisted assembly further organized this genome into eight chromosomes (2*n* = 16), achieving a mapping rate of 97.93% when raw Illumina reads were aligned to the genome. The assembly metrics showed that the contig N50 reached 1.95 Mb, and the scaffold N50 reached 67.23 Mb (Figure S5, Table S5). Genome annotation predicted 31 976 genes and 8929 noncoding RNAs, with over 50% of the genome comprising repetitive sequences and transposable elements. In summary, *Sm*.SC has a larger genome size, more comprehensive reads coverage and higher assembly integrity than *Sm*.SD and *Sm*.SX (Table S4). The high‐quality data generated in this work also provides an expansion from Labiatae for the pan plant genome project (Liu *et al*., 2024a).

**Figure 2:**
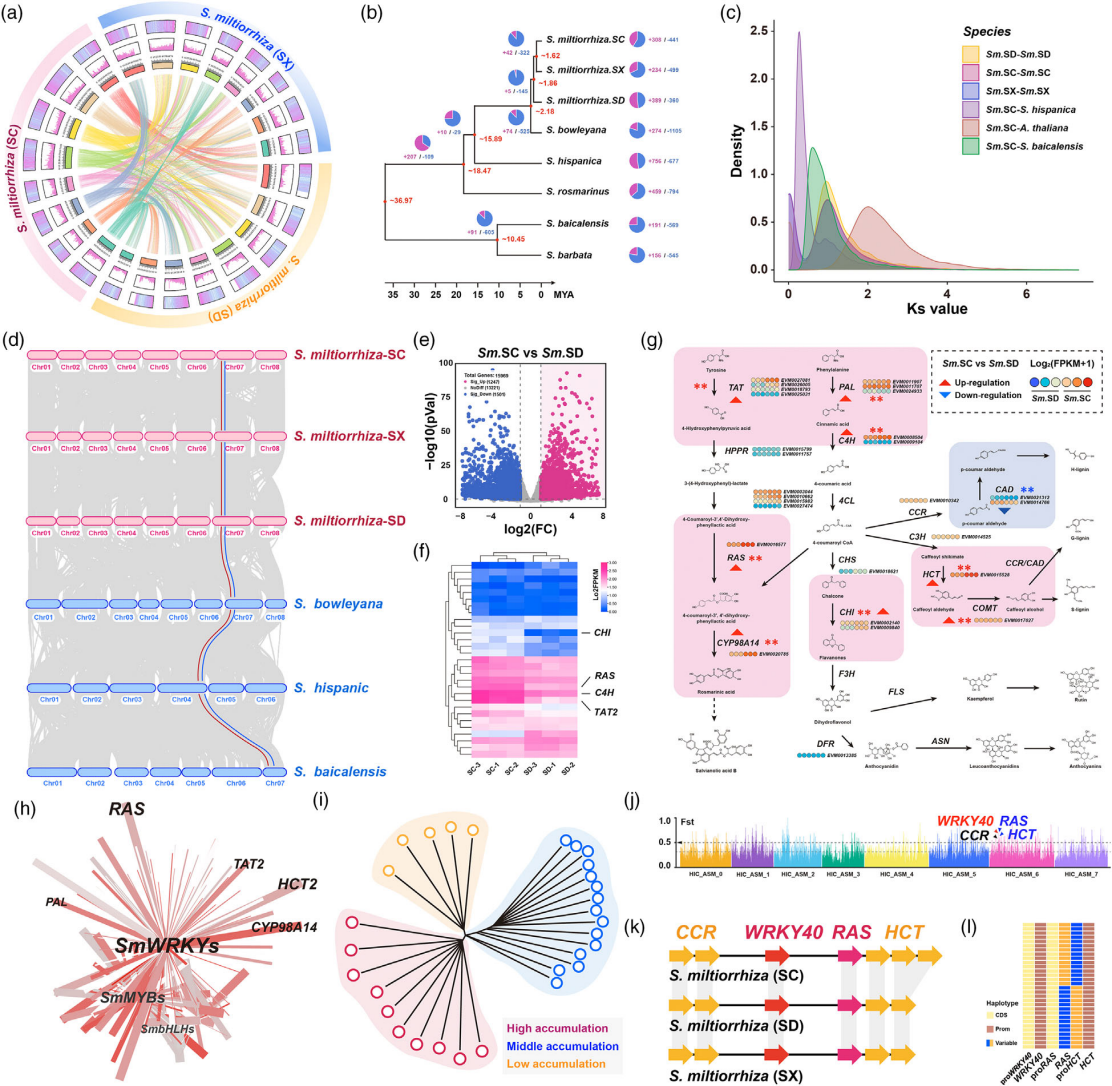
Genome assembly, comparative genomics, population variation and gene cluster mining based on Sichuan *S. miltiorrhiza*. (a) Collinearity circos plot of the genomic features of *S. miltiorrhiza* from Sichuan, Shaanxi and Shandong. (b) The phylogenetic tree was constructed using 2211 single‐copy genes from 6 Labiaceae species. All branches in the tree had posterior probabilities exceeding 0.99. Pie charts and the corresponding numbers represent the expansion and reduction of gene families. (c) Distribution of synonymous substitution rates (Ks) for paired syntenic paralogs was analysed in *S. miltiorrhiza* of 3 ecotypes and three other plants. (d) Synteny maps were generated to compare *S. miltiorrhiza* from Sichuan, Shaanxi, Shandong, *S. bowleyana*, *S. hispanic* and *S. baicalensis*. Light grey lines represent synteny blocks. The colours of connecting lines indicate representative metabolic modules with a high degree of evolutionary conservation. Red lines indicate rosmarinic acid synthase (*RAS*) gene. Blue lines indicate the *BAHD* acyltransferase genes. (e) Volcano plot drawn from RNA‐seq differentially expressed gene (Fold Change > 2, *P* < 0.05) data set from mature roots of Sichuan and Shandong *S. miltiorrhiza*. (f) Heatmap of differentially expressed gene sets derived from tyrosine and phenylalanine metabolic pathway. (g) Gene expression data were mapped to metabolic pathways, with red modules indicating up‐regulated pathways and blue modules indicating down‐regulated pathways. *P < 0.05; **P < 0.01. (h) Among the co‐expression networks formed between tyrosine and phenylalanine metabolic pathway genes and transcription factors, the WRKY TFs have the highest network flux. (i) Neighbour‐joining tree of 30 *S. miltiorrhiza* germplasms, including 10 high phenolic acid, 15 middle phenolic acid and 5 low phenolic acid accumulation germplasms. Branch colours indicate different groups. (j) Highly divergent genomic regions between different germplasm. The horizontal dashed line indicates the top 5% of *F*st and the marked by the letter indicate gene cluster in the highly divergent regions of the phenolic acid metabolism pathways. (k) A key gene cluster of lignin synthase *CCRs* and *HCTs* tandem repeating phenolic acids upstream and downstream centred on transcription factor gene *SmWRKY40* was identified within the selected clearance interval. (l) Haploid analysis of promoters and coding regions of several genes in gene clusters. The same colour represents the same haplotype.

To contextualize these findings, we constructed a comparative genomic map with *Sm.SX* and *Sm.SD* genomes (Figure 2a). Analysis of gene family expansion and contraction (Figure 2b) and evolutionary rates (Figure 2c) indicated that *Sm.SX* diverged from *Sm.SD* approximately 1.86 million years ago, with *Sm.SC* diverging from *Sm.SX* around 1.62 million years ago. The phylogenetic tree, built from 2211 orthologous single‐copy genes across the Lamiaceae family, highlighted 526 gene family expansions and 615 contractions unique to *Sm.SC*. GO and KEGG enrichment analyses identified expanded pathways linked to phenylalanine metabolism, acyltransferase activities and the WRKY transcription factor family, while contracted pathways were primarily related to photosynthesis and tyrosine metabolism (Table S6).

Despite strong genomic collinearity across the Lamiaceae family, we observed chromosomal inversions in *Sm.SX* and *Sm.SD* (Figure 2d). Given these similarities, but marked differences in phenotype and metabolite content, we focused on a comparative analysis between *Sm.SC* and *Sm.SD*. Transcriptome analysis identified 1247 upregulated and 1501 downregulated genes in *Sm.SC*, many of which are involved in phenolic acid metabolism, such as TAT2, C4H, CHI and RAS (Figure 2e,f). Mapping these genes on a metabolic pathway map revealed upregulation in upstream (TAT and PAL) and downstream (RAS and CYP98A) genes, while CAD genes were downregulated in *Sm.SC* (Figure 2g). Metabolomic profiling showed elevated levels of L‐phenylalanine, L‐tyrosine, salvianolic acid B (SAB) and S‐type lignin precursors in *Sm.SC*, while downstream lignin monomers like coniferin were reduced (Figure S7a).

In constructing a transcriptional regulatory network, we identified WRKY family transcription factors as key regulators in the phenolic acid pathway. Further comparison with the *Arabidopsis* genome database (TAIR, https://www.arabidopsis.org/), based on gene sequence homology, a WRKY gene located at the core of the regulatory network was defined as *SmWRKY40*, which shows a particularly strong association with RA, SAB and S‐type lignin components (Figure S7b,c, Figure 2h, Tables S8 and S9). We further examined genetic diversity by resequencing 30 individuals with varying phenolic acid levels, generating data on 581 286 SNPs and 19 788 InDels (Tables S11 and S12). Phylogenetic analysis based on these SNPs identified 27 280 selective sweep intervals across three subpopulations (Figure 2j, Table S13). The *SmWRKY40* gene was found within a gene cluster of lignin synthesis‐related genes, including CCR and HCT, indicating a tandem duplication event that serves as a molecular switch for regulating lignin monomer synthesis (Figure 2j,k). This gene cluster's origins trace back to early plant evolution (Kruse *et al*., 2022, 2023), with HCT and WRKY40 conserved across angiosperms, while RAS is specific to Lamiaceae (Figure S8a–e). SmRAS (*SmiChr070974*) and *SmWRKY40* (*SmiChr070967*), as central elements in the cluster, showed significantly higher expression in *Sm.SC* than in *Sm.SD* (Figure S8f,g). Although the cluster displayed population‐level genetic variation, including InDels in HCT promoter regions and SNPs in RAS coding regions, *SmWRKY40* remained highly conserved (Figure 2l).

### *SmWRKY40* regulates phenolic acid metabolism and cell wall development in *Salvia miltiorrhiza* through transcriptional activation of key enzymes

We cloned the open reading frame (ORF) of *SmWRKY40* and inserted it into the pCAMBIA‐1305 expression vector to verify its nuclear localization in tobacco leaves via a transient expression system (Figure S9a). The transcriptional activation function of the N‐terminal WRKY domain was then confirmed (Figure S9b). Quantitative PCR (qPCR) analysis showed that *SmWRKY40* was highly expressed in the roots of both *S. miltiorrhiza* cultivars, *Sm.SC* and *Sm.SD* (Figure S9c). *SmWRKY40* expression was significantly upregulated in response to abscisic acid (ABA) and yeast extract, as well as under various stress conditions, including UV‐B exposure, low temperature, NaCl and drought (Figure S9d).

**Figure 3:**
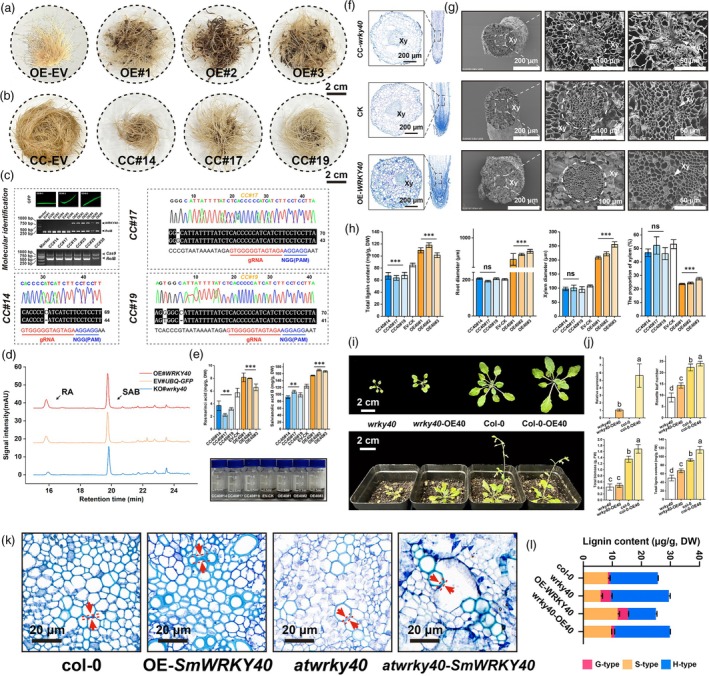
*SmWRKY40* promote root development and improve the accumulation of rosemic acid, salvianolic acid B and total lignin. (a, b) *S. miltiorrhiza* hairy root with overexpression (a) and knockout (b) of the *SmWRKY40* gene. CC, CRISPR‐Cas9; EV, Empty vector; OE, overexpression. (c) Transgenic PCR identification and sequencing validation of CRISPR‐Cas9 mediated gene editing sites. (d) Roseminic acid and salvianolic acid B content in hairy roots by HPLC. (e) Analysis of rosmarinic acid, salvianolic acid B and total phenolic acid in hairy roots. (f, g) Overexpression and knockout of the dissected structure of hairy roots, and observation of the effects of *SmWRKY40* on the development of hairy root xylem using toluidine blue staining (f) and scanning electron microscopy (g), respectively. (h) Analysis of the phenotype and lignin content performed on hairy roots. (i) Comparison of phenotypes between transgenic Arabidopsis with heterologous expression of *SmWRKY40* and after supplementation with *SmWRKY40* in *atwrky40* mutant. (j) Phenotypic statistics and lignin content determination of Arabidopsis roots. (k) Histochemical stain of transgenic Arabidopsis with heterologous expression of *SmWRKY40* and after supplementation with *SmWRKY40* in *atwrky40* mutant. (l) Three kinds of lignin monomer components were quantitatively detected by GC–MS. All data show the arithmetic mean ± SD from 3 biological replicates. Different letters indicate significant differences at *P* < 0.05 (one‐way ANOVA, Tukey's posttest). *P < 0.05; **P < 0.01; ***P < 0.001.

Using a hairy root transformation system (Figure S10), we observed that *SmWRKY40* overexpression promoted the growth of transgenic hairy roots (Figure 3a), while CRISPR‐Cas9‐mediated knockout of *SmWRKY40* resulted in reduced growth and a softer root texture (Figure 3a–c). High‐performance liquid chromatography (HPLC) analysis confirmed that *SmWRKY40* positively regulated the accumulation of phenolic acids, specifically rosmarinic acid (RA) and salvianolic acid B (SAB), in transgenic hairy roots (Figure 3d,e). Toluidine blue staining and scanning electron microscopy further revealed that xylem duct development was more advanced in *SmWRKY40*‐overexpressing hairy roots, while xylem formation was significantly impaired in *smwrky40* mutants (Figure 3f,g). These findings were corroborated by morphological examination and lignin composition analysis of the hairy roots (Figure 3h).

To elucidate *SmWRKY40*'s role in phenolic acid metabolism, we assessed the transcript levels of key enzymes in this pathway. qPCR analysis showed that *SmWRKY40* positively regulated the expression of critical enzymes, including RAS, CYP98A14 and CCR2 (Figure S11). Transgenic *Arabidopsis* lines overexpressing *SmWRKY40*, as well as *atwrky40* mutant lines complemented with *SmWRKY40*, were generated (Figure S12). Comparative phenotypic analysis and lignin component quantification in these lines indicated that *SmWRKY40* promotes growth, development and lignin biosynthesis (Figure 3i,j). Histological staining (Figure 3k) and analysis of lignin monomer composition (Figure 3l). The functional characterization in the ontology and heterologous systems confirmed that *SmWRKY40* regulates cell wall thickening and promotes syringyl lignin accumulation, further highlights its role in lignin and cell wall metabolism, and suggests that *WRKY40* is highly conserved for the regulatory function of plant cell wall and root development, while its complex evolution in Lamiaceae broadens the diversity of secondary metabolic networks of plant kingdom.

### *SmWRKY40* directly regulates phenolic acid and tyrosine metabolism in *Salvia miltiorrhiza* through ecotype‐specific interactions

**Figure 4:**
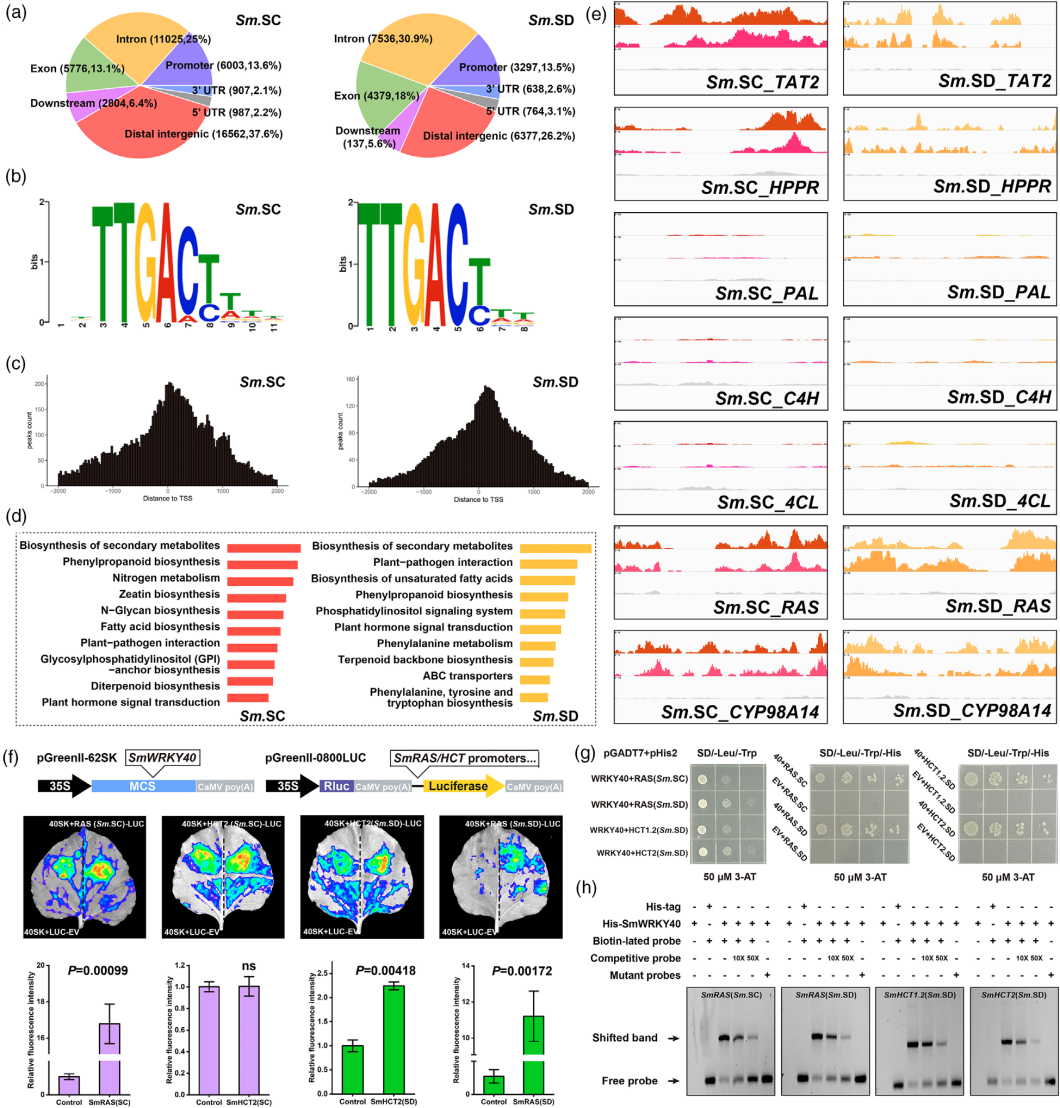
DAP‐seq identify SmWRKY40 diverged on the transcriptional regulation of rosmarinic acid and lignin biosynthesis pathway in different ecotypes of *S. miltiorrhiza*. (a) Distribution of candidate SmWRKY40‐binding regions across 2 sets of S. miltiorrhiza reference genomes (Sichuan and Shandong ecotypes) as determined by DAP‐seq. (b) Motif analysis using HMMER to identify core motifs enriched within the experimentally determined (by DAP‐seq) SmWRKY40‐binding regions. (c) Distribution of binding regions enriched at the transcription start site (TSS). (d) Functional enrichment of target genes bound to the promoter region by SmWRKY40. (e) Displayed the binding sites of SmWRKY40 in the promoter region of the genes involved in the tyrosine and phenylalanine metabolism pathways of *S. miltiorrhiza* in Sichuan (red peaks) and Shandong (yellow peaks). Grey peaks represent Input. (f–h) Protein‐DNA interactions were investigated through Daul‐LUC (f), Yeast one‐hybrid assay (g) and EMSA (h).

To further investigate the transcriptional regulatory function of *SmWRKY40*, we purified GFP‐tagged *SmWRKY40* protein and incubated it with genomic libraries from *Sm.SC* and *Sm.SD*. Using a DNA affinity purification sequencing (DAP‐seq) approach, we conducted high‐throughput screening to identify potential binding sites of *SmWRKY40*. In total, we identified 22 632 binding sites from the *Sm.SC* library and 9584 from the *Sm.SD* library (Table S14). Approximately 13.5% of these sites were located in promoter regions, 25% and 30% in intronic regions and 13.1% and 18% in exonic regions for *Sm.SC* and *Sm.SD*, respectively (Figure 4a). Binding motif analysis revealed a specific affinity of *SmWRKY40* for W‐box elements (TTGACT/C) present in both libraries (Figure 4b). The distribution patterns of these binding sites relative to transcription initiation sites were notably similar across the two ecotypes (Figure 4c).

Gene ontology analysis of the gene set highly enriched in promoter regions linked these regulatory interactions to distinct biological processes. In *Sm.SC*, we found a strong enrichment of genes related to phenylalanine/tyrosine metabolism, notably including two key tyrosine metabolism genes, *SmTAT2* and *SmHPPR* (Figure 4d). In contrast, *Sm.SD* showed a higher enrichment of genes associated with plant‐pathogen interactions, photosynthesis and hormone signalling pathways, suggesting that *SmWRKY40* target genes have diversified with ecotype differentiation (Figure 4d). Within the phenolic acid metabolism pathway, *SmWRKY40* consistently regulated both upstream tyrosine metabolism genes and downstream phenylalanine pathway genes in both ecotypes (Figure 4e). However, *SmWRKY40* did not bind to the *SmHCT* promoter in *Sm.SC*, whereas in *Sm.SD*, this regulatory interaction, conserved from a shared ancestor, was retained within the *CCR‐HCT* gene cluster (Figure S13a,b).

The physical interactions between *SmWRKY40* and the promoters of target genes were validated through dual‐luciferase (dual‐LUC) assays (Figure 4f), yeast one‐hybrid assays (Figure 4g) and electrophoretic mobility shift assays (EMSA) (Figures 4h and S14). These results collectively confirmed that *SmWRKY40* binding patterns for *SmTAT2*, *SmHPPR*, *SmRAS*, *SmCYP98A14* and *SmCCR* were consistent across ecotypes. However, the lack of *SmWRKY40* binding to the *SmHCT* promoter in *Sm.SC* corresponded with a marked reduction in the accumulation of S‐type lignin in the roots (Figure S13c).

### Distinct haplotypes of the RAS gene drive metabolic divergence in *Salvia miltiorrhiza* populations

We performed a comparative analysis of genomic data from *Sm.SC* and *Sm.SD*, complemented by population resequencing. This analysis revealed two distinct haplotypes of the *RAS* gene: the 146C‐haplotype in *Sm.SC* and the 146G‐haplotype in *Sm.SD* (Figure S15a,b). A single nucleotide variation between these haplotypes results in a critical amino acid change, substituting nonpolar glycine (Gly) with polar alanine (Ala), thereby causing the loss of a β‐turn in the protein's secondary structure. This structural alteration was further confirmed in a three‐dimensional protein model (Figure S15c). To assess the population distribution of these haplotypes, we designed cloning primers targeting the first exon containing the SNP. PCR amplification and Sanger sequencing enabled us to evaluate haplotype frequencies across populations (Figure S15d,e). Our findings indicated that populations with the 146C‐haplotype exhibited significantly higher levels of RA and SAB compared to those with the 146G‐haplotype (Figure S16a,b). Moreover, a positive correlation was observed between RA and SAB contents and root acyltransferase activity within these populations (Figure S16C,D).

**Figure 5:**
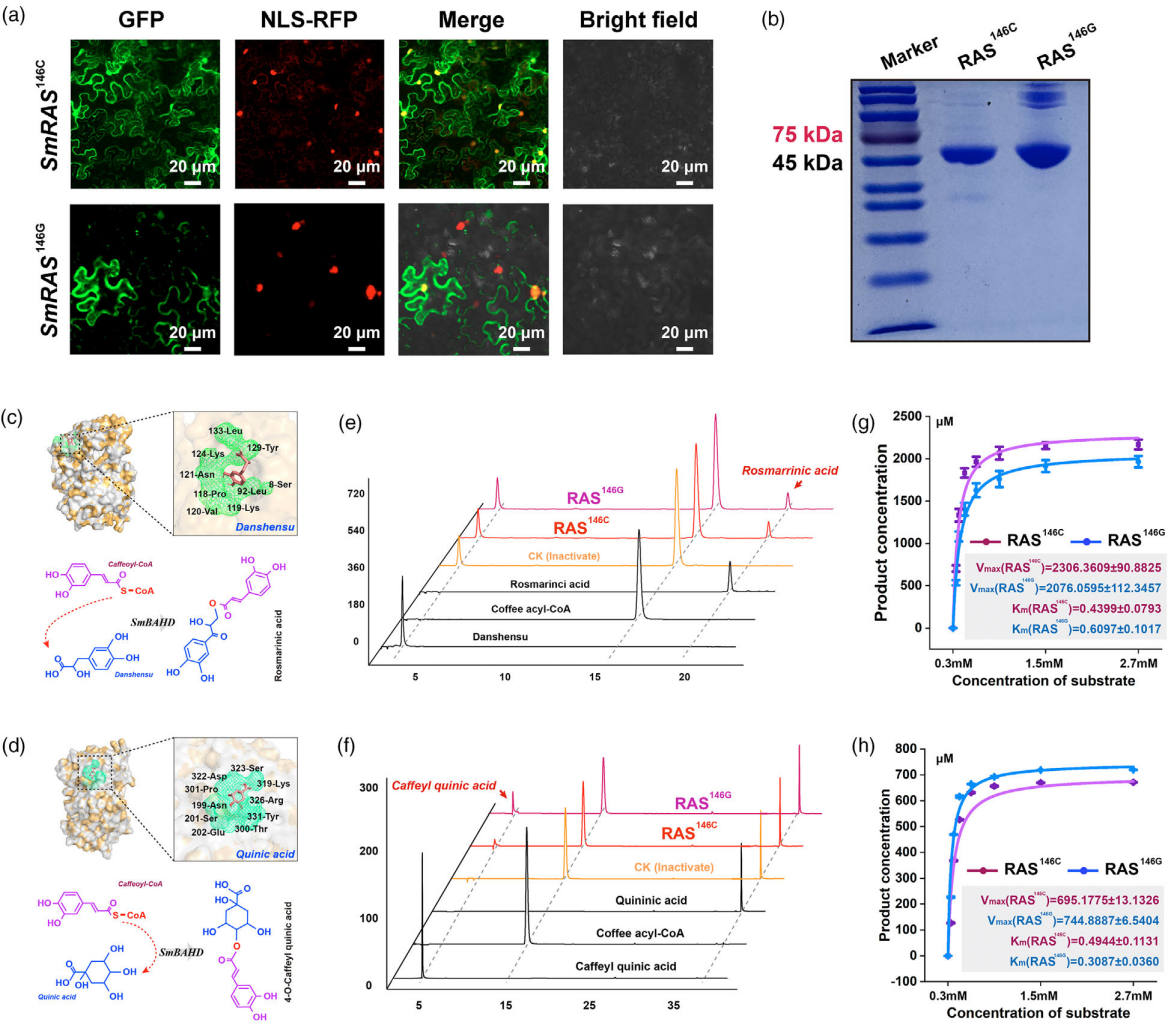
Comparison of the subcellular localization and kinetic characteristics of the RAS enzymes for the different haplotypes. (a) Subcellular localization of SmWRKY40. Green fluorescence (GFP) represents the RAS^146C^ and RAS^146G^ recombinant protein, and red fluorescence (RFP) represents the nuclear marker. The superimposed images show yellow fluorescence. (b) Purified pET28a‐RAS (146C and 146G) recombinant plasmid was introduced in the *E. coli* expression system, induced at 0.2 μM iptg and purified protein obtained through a Ni chromatography column. (c) Molecular docking simulations and enzymatic reaction mechanism of caffeoyl‐CoA and quinic acid with RAS proteins. (d) Molecular docking simulations and enzymatic reaction mechanism of caffeoyl‐CoA and danshensu with RAS proteins. (e) High‐performance liquid chromatography (HPLC) showing the formation of rosmarinci acid during *in vitro* enzyme assays with recombinant RAS^146C^ and RAS^146G^ using caffeoyl‐CoA and danshensu as substrates, compared to an authentic standard of corresponding compounds. (f) High‐performance liquid chromatography (HPLC) showing the formation of rosmarinci acid during *in vitro* enzyme assays with recombinant RAS^146C^ and RAS^146G^ using caffeoyl‐CoA and danshensu as substrates, compared to an authentic standard of corresponding compounds. (g, h) Michaelis–Menten curves of RAS catalysing the enzymatic reaction of caffeyl quinic acid (g) and rosmarinci acid (h) pathways.

We cloned the two haplotypes of the *RAS* gene from *Sm.SC* and *Sm.SD* cDNA. Subcellular localization experiments confirmed that the SNPs did not affect the localization of the RAS protein, which was observed in both the nucleus and cell membrane (Figure 5a). Both haplotype variants were expressed and purified using an *E. coli* expression system (Figure 5b), and their optimal enzymatic activity conditions were determined to be pH 8.0 at 25 °C (Figure S16e,f). Molecular docking simulations indicated that RAS retains both RA synthase activity and the ancestral acyltransferase activity typical of the BAHD acyltransferase family, suggesting potential catalytic activity for S‐type lignin precursors (Figure 5c,d). *In vitro* enzyme assays confirmed that RAS catalyses the polymerization of caffeoyl‐CoA and danshensu to form RA (Figure 5e) and can also use caffeoyl‐CoA as an acyl donor with quinic acid as an acyl acceptor to produce caffeoyl quinic acid (Figure 5f).

Kinetic analysis revealed differing substrate preferences: the 146C‐haplotype RAS showed a higher affinity for RA precursor substrates (Figure 5g), while the 146G‐haplotype RAS displayed a preference for caffeoyl quinic acid precursors (Figure 5h). These results highlight the role of SNPs as a key driver of metabolic diversity within *S. miltiorrhiza* populations.

### The effect of different haplotypes of *RAS* on the synthesis of rosmarinic acid and S‐type lignin precursor in *S. miltiorrhiza* hairy roots

**Figure 6:**
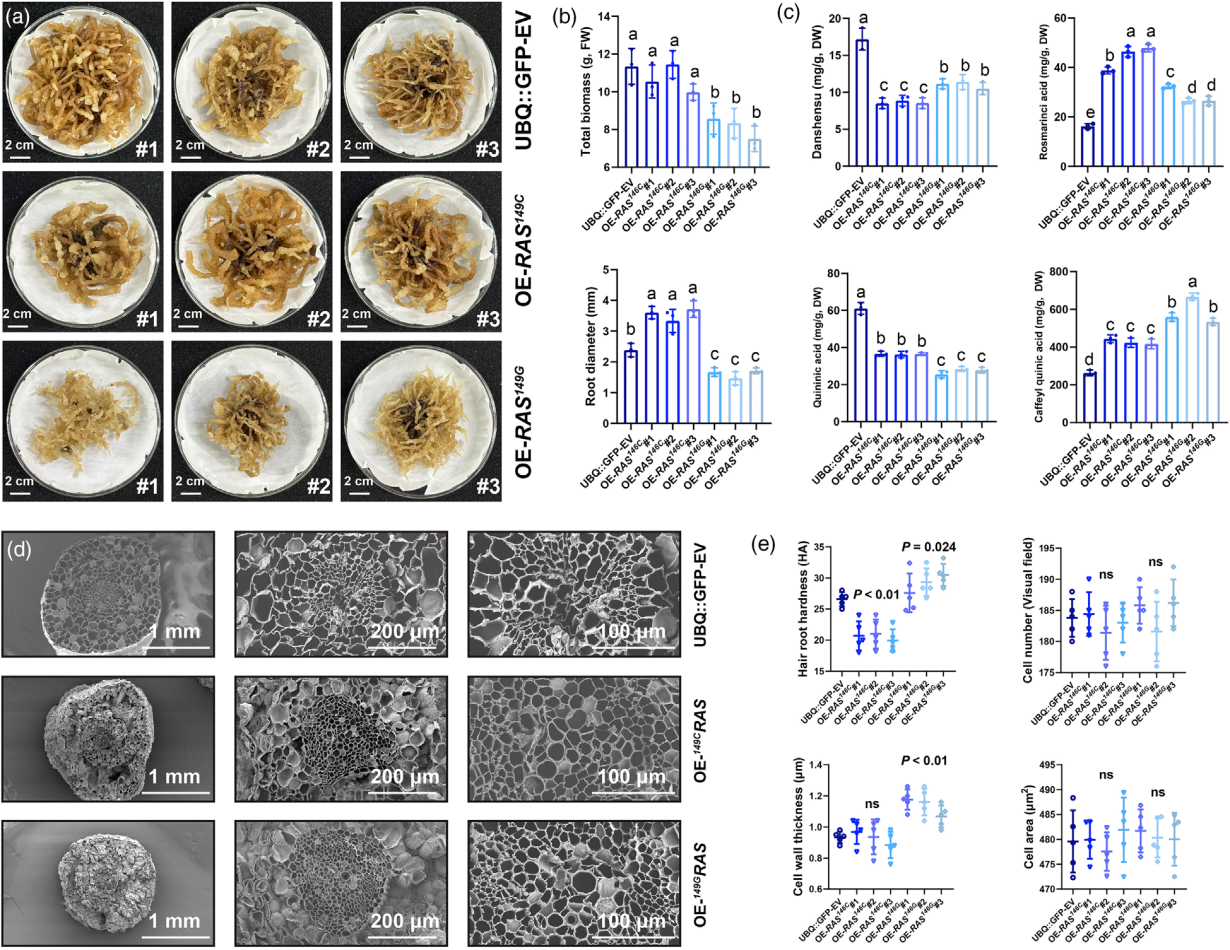
Phenotypic, compositional and histological comparison of two RAS haplotypes transgenic in hairy roots. (a) Two RAS haplotypes transgenic in hairy roots. (b) Phenotypic statistics of the transgenic hairy roots. (c) Comparison of substrate and products of rosmarinic acid and lignin pathway in transgenic hairy roots. (d) SEM imaging of the transgenic hairy roots. (e) Statistics on hardness, cell wall thickness, cell cross‐sectional area and cell number of transgenic hairy roots. All data show the arithmetic mean ± SD from 3 biological replicates. Different letters indicate significant differences at *P* < 0.05 (one‐way ANOVA, Tukey's posttest).

On the premise of fully characterizing the differences in enzyme kinetics *in vitro*, we further constructed transgenic lines of *SmRAS* ^146C^ and *SmRAS* ^146G^ through the hairy root transformation system (Figure 6a). Compared with the UBQ‐GFP lines, the growth and development of the OE‐*SmRAS* ^146C^ hairy root strain were significantly promoted, manifested by more significant increases in root length, root diameter and total biomass (Figure 6b). The growth and development promotion of OE‐*SmRAS* ^146G^ hairy roots is not significant, and their coarseness actually decreases (Figure 6b). The contents of danshensu, rosmarinic, quinic acid and caffeoylquinic acid in OE‐*SmRAS* ^146C^ and OE‐*SmRAS* ^146G^ hairy roots were specifically detected via HPLC system, representing the downstream pathways of danshensu and lignin, respectively (Figure 6c). It is worth noting that OE‐*SmRAS* ^146C^ hairy roots consume more danshensu and accumulate more rosmarinic acid, while OE‐*SmRAS* ^146G^ hairy roots consume more quinic acid and accumulate more caffeoylquinic acid (Figure 6c). The anatomical characterization of xylem development confirms that the cell wall of OE‐*SmRAS* ^146G^ hairy roots thickens, while the xylem of OE‐*SmRAS* ^146C^ hairy roots tends to aggregate towards the centre, which may be an intrinsic mechanism leading to the plasticity of root hardness and phenotypic diversity in *S. miltiorrhiza* (Figure 6d,e).

### SmRAS haplotypes differentially regulate lignin biosynthesis and root development in *athct2* Arabidopsis mutants

**Figure 7:**
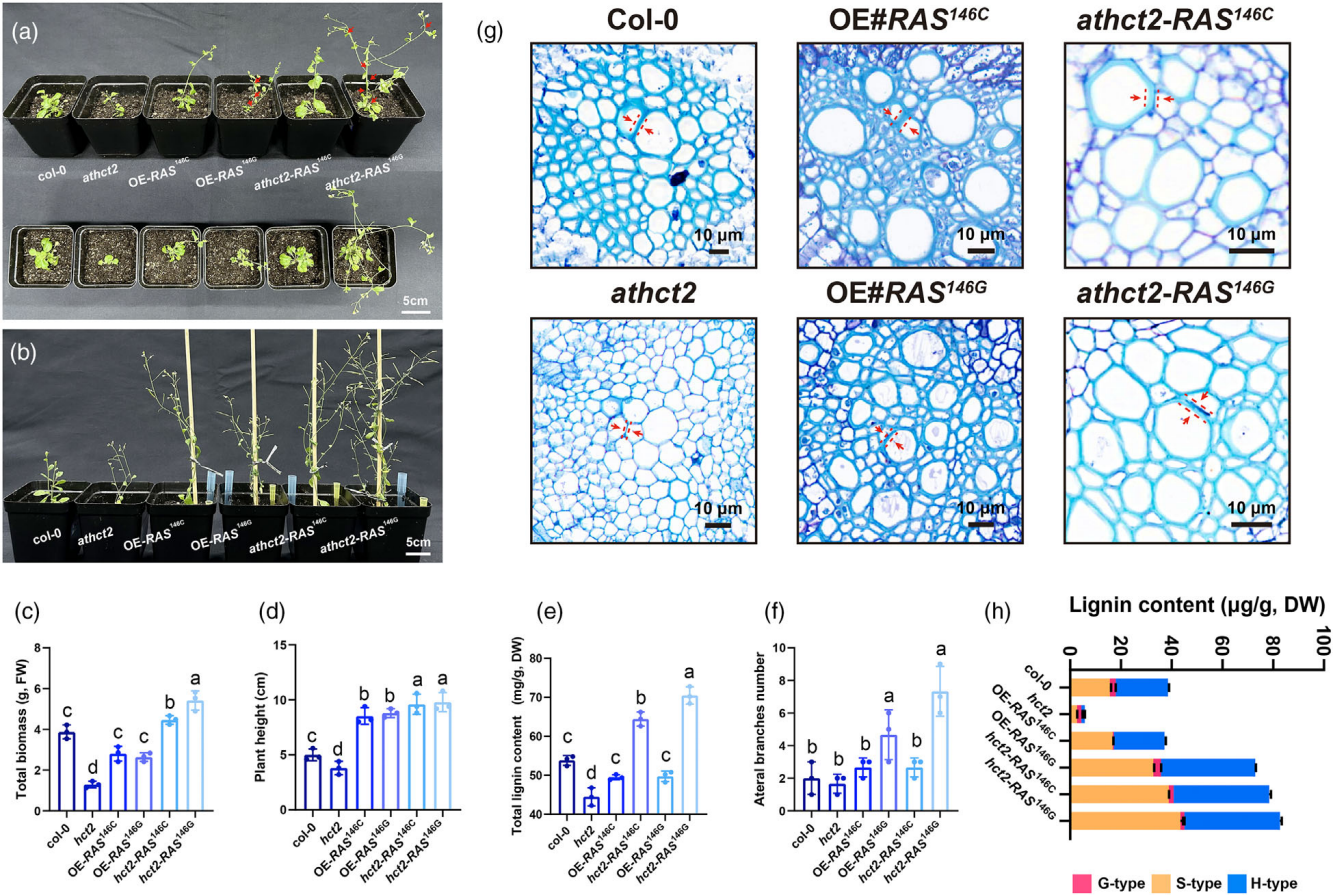
The replacement phenotypes of two haplotypes of *SmRAS* in Arabidopsis *athct2* mutants. (a, b) Phenotypic comparison of 6‐week and 10‐week seedling ages in Arabidopsis with different genotypes. (c–f) Statistics on total biomass, plant height, total lignin content in roots and number of lateral branches in aboveground parts of different genotypes of Arabidopsis plants at 10 weeks of seedling age. (g) Histochemical staining of roots in Arabidopsis plants with different genotypes. (h) Quantitative detection of three lignin monomer components in the roots of different genotypes of Arabidopsis plants using GC–MS. All data show the arithmetic mean ± SD from 3 biological replicates. Different letters indicate significant differences at *P* < 0.05 (one‐way ANOVA, Tukey's posttest).

Using homologous identification and multiple sequence alignment, we identified *AtHCT2* as a homologue of *SmRAS* (Figure S17a,b). We then generated *athct2* mutants (Figure S18a,b) and expressed different *SmRAS* haplotypes in both wild‐type *Col‐0* and *athct2* mutant *Arabidopsis* lines (Figures 7a,b and S18). While overexpression of *SmRAS* in the *Col‐0* background inhibited *Arabidopsis* growth and development, it alleviated growth deficits in the *athct2* mutants (Figure 7a,b). Notably, the OE‐*SmRAS* ^146G^ haplotype exhibited a stronger effect in promoting lateral branching and lignin accumulation in *athct2* mutants (Figure 7a,b).

Quantitative phenotype and lignin content analyses supported these findings (Figure 7c–f). Additionally, histochemical staining and quantitative assays of lignin monomers showed that *HCT2* positively regulates syringyl lignin content and cell wall thickening. The *athct2* mutant phenotypes were effectively rescued by *RAS* overexpression, with OE‐*RAS* ^146G^ demonstrating a more substantial compensatory effect than OE‐*RAS* ^146G^ (Figure 7g,h).

### *SmWRKY40* and *SmRAS* haplotypes modulate stress response by balancing antioxidant activity and reducing oxidative damage

**Figure 8:**
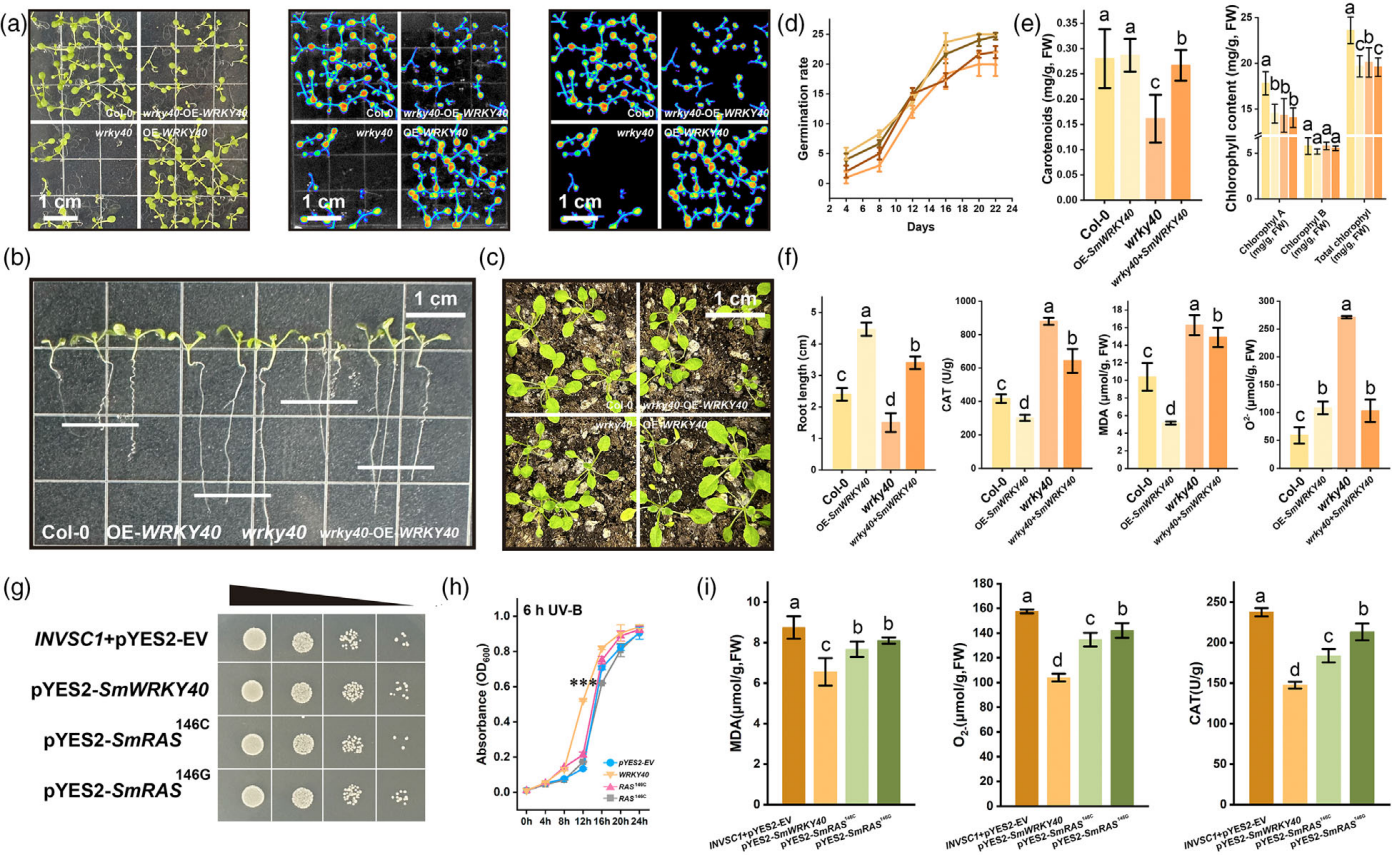
Heterologous expression validates the biological functions of *SmWRKY40* and *SmRASs* in response to UV‐B exposure. (a) Plate growth phenotype of wild‐type (col‐0), OE‐*SmWRKY40*, *atwrky40* mutant and *SmWRKY40* complemented *atwrky40* mutant *A. thaliana* under UV‐B exposure. The fluorescence intensity reflects the chlorophyll fluorescence in the plant leaves, and the stronger the fluorescence intensity the higher the plant's biological activity. (b) Root length of different genotypes of *Arabidopsis* under UV‐B exposure. (c) Phenotypes of different genotypes of *Arabidopsis* under UV‐B exposure. (d) Germination rate statistics of *Arabidopsis* under UV‐B exposure for different genotypes. (e) Carotenoid and chlorophyll content of different genotypes of *Arabidopsis* under UV‐B exposure. (f) Phenotypic statistics and antioxidant physiology assays of different genotypes of *Arabidopsis* under UV‐B exposure. (g) Growth status of yeast transferred with *pYES2* vector, *pYES2*‐*SmWRKY40*, *pYES2*‐*RAS* ^146C^ and *pYES2*‐*RAS* ^146G^ on plates under UV‐B exposure. (h) Growth curves of yeast with different genotypes under UV‐B exposure. (i) Antioxidant physiological detection of yeast with different genotypes under UV‐B exposure. All data show the arithmetic mean ± SD from the three biological replicates. Different letters indicate significant differences at *P* < 0.05 (one‐way ANOVA, Tukey's posttest).

To investigate the conserved biological functions of *SmWRKY40* in stress adaptation across species, we used heterologous expression and mutant complementation platforms. Our findings indicate that *SmWRKY40* enhances tolerance to UV‐B (Figure 8a–f) and cold stress (Figure S19a–c) while increasing sensitivity to Cu^2+^ stress (Figure S20a–c). Under UV‐B stress, *SmWRKY40* overexpression in *Arabidopsis* significantly improved seed germination rates (Figure 8a,b,d) and promoted root elongation in seedlings (Figure 8b). Conversely, the *atwrky40* mutant showed pronounced developmental defects under UV‐B stress, partially mitigated by reintroducing *SmWRKY40*. These effects were consistent in both controlled tablet and potted plant experiments (Figure 8c). Phenotypically and physiologically, the positive influence of *SmWRKY40* under UV‐B stress was evidenced by increased photosynthetic pigment accumulation (Figure 8e), reduced catalase (CAT) activity and lower levels of malondialdehyde (MDA) and superoxide anions (O_2_^−^) (Figure 7f).

We also generated transgenic yeast strains expressing *SmWRKY40* and the two *SmRAS* haplotypes (146C and 146G). In yeast, *SmWRKY40* overexpression enhanced growth under UV‐B and cold stress but intensified toxicity under Cu^2+^ stress, with these effects evident in both plate assays (Figures 8g, S19d and S20d) and liquid culture growth curves (Figures 8h, S19e and S20e). The 146C‐haplotype of *SmRAS* conferred slightly greater stress adaptation than the 146G‐haplotype, although both haplotypes showed a lesser effect on stress responses compared to transcription factor overexpression (Figures 8g,h, S19d,e and S20d,e). Consistent with the *Arabidopsis* results, *SmWRKY40* and *RAS* in yeast modulated stress responses primarily by balancing CAT activity and reducing MDA and O_2_^−^ accumulation (Figure 8i). These findings deepen our understanding of stress response regulation through the interplay between transcription factors and metabolic enzymes.

## Discussion

This study utilized a multi‐omics approach to investigate the genetic and environmental factors shaping the soft‐root phenotype and high phenolic acid content in *S. miltiorrhiza* (Danshen), an important TCM herb. By examining germplasm from multiple regions across China, we identified distinct environmental adaptations influencing phenotypic and biochemical traits, especially in the Sichuan ecotype (*Sm.SC*). Our analysis revealed that specific environmental stressors, notably UV‐B radiation and low temperatures, stimulate the biosynthesis of rosmarinic acid (RA) and salvianolic acid B (SAB). Furthermore, genetic profiling uncovered a phenolic acid metabolic gene cluster unique to *Sm.SC*, centred around *SmWRKY40*. This transcription factor appears to be a key regulator of phenolic acid and lignin synthesis, suggesting it has a critical role in determining root texture and phenolic content.

Our findings emphasize the dual roles of genetic and environmental factors in shaping *S. miltiorrhiza*'s key traits, such as RA and SAB accumulation, which are integral to its medicinal value (Shi *et al*., 2024; Xu *et al*., 2016). A basic consensus is that salvianolic acid levels vary between ecotypes in response to both genetic background and environmental conditions, such as UV exposure (Yin *et al*., 2020) and salt stress (Lv *et al*., 2024), which induce secondary metabolite pathways. Previous work focused more on the regulatory functions of key genes themselves during the secondary metabolic process, and environmental factors such as abiotic stress (Lv *et al*., 2024), hormone signalling (Li *et al*., 2025b) and light signalling (Yin *et al*., 2020) affect metabolite accumulation only by altering gene expression. An innovative point of this work lies in the bridge between environmental perturbations and genomic variation, a new perspective of genome evolution driven by environmental factors and how the dynamic balance of the two affects the quality of medicinal plants. We expand on these insights by detailing how environmental cues modulate phenolic acid metabolism in *S. miltiorrhiza*. The identification of the *SmWRKY40*‐regulated gene cluster adds a new layer of understanding, linking genetic regulation directly to environmental responsiveness in promoting essential metabolites.

Our study also revealed a positive correlation between UV‐B and cold stress and the accumulation of RA and SAB, paired with a decrease in lignin content under copper (Cu) stress, which is highly consistent with previous reports (Yin *et al*., 2020; Zhong *et al*., 2024). This pattern suggests a trade‐off in resource allocation within the root's biochemical pathways, with stress responses prioritizing phenolic acid over lignin production. This evidence also skillfully echoes the recently proposed biomechanical theory of medicinal plants, emphasizing the internal mechanism of mechanical structure affecting the quality of traditional herbs (Wang *et al*., 2025). These metabolic shifts are supported by histological observations: the distinct clustered and circular lignin arrangement in *Sm.SC* roots, as opposed to the radial distribution seen in other ecotypes, contribute to a softer root texture. This structural adaptation indicates that cellular organization significantly affects root biomechanics and that phenolic acid levels may be preserved through these adaptive cellular modifications.

Our findings further support the concept that genetic clusters serve as centralized regulators of metabolic adaptation in plants (Li *et al*., 2025a; Zhao *et al*., 2023). Specifically, the regulation of phenolic acid and lignin synthesis by the *SmWRKY40* cluster underscores the idea that adaptive mechanisms operate at both genomic and cellular levels. Moreover, our results corroborate the idea that secondary metabolites like RA and SAB, which are often associated with plant stress tolerance, are accumulated in response to specific environmental stressors. The function of *SmWRKY40* reinforces the role of transcriptional networks in facilitating adaptive growth strategies, where the modulation of phenolic acid levels is closely linked with root structural adjustments to optimize resilience and resource allocation.

Previous studies have shown that *AtWRKY40* can significantly alleviate retrograde signal‐induced mitochondrial stress and chloroplast damage in plants (Olivier *et al*., 2013), and play an irreplaceable role in plant adaptation to drought stress (Che *et al*., 2018). The heterologous expression and mutant complementation of *SmWRKY40* in *Arabidopsis* further confirmed its involvement in plant stress resistance and expanded its biological functions in cell wall development and secondary metabolism regulation. However, several limitations in this study warrant discussion. Although we identified environmental triggers and genetic regulators influencing phenolic acid content, the exact signalling pathways linking these external cues to *SmWRKY40* expression remain undefined. Future studies should focus on upstream signalling molecules and pathways, including hormone‐mediated responses, that may govern *SmWRKY40* regulation in response to environmental changes (Li *et al*., 2025a). Furthermore, field validation of our findings in broader environmental settings would provide a more comprehensive understanding of genotype‐environment interactions. The genetic variation observed in *SmWRKY40* between ecotypes also suggests a potential avenue for selective breeding to enhance phenolic content, which could have practical applications in *S. miltiorrhiza* cultivation.

In summary, this study provides valuable insights into the convergence of genetic and environmental factors that shape key traits in *S. miltiorrhiza*. By advancing our understanding of how these factors interact to influence phenolic acid levels and root texture, our findings open new avenues for optimizing the quality of traditional Chinese medicine through targeted breeding and environmental management.

## Methods

### Experimental model and subject details

Four different ecotypes of *S. miltiorrhiza* were used. The Sichuan ecotype (Chuandanshen No. 1, CDS‐1) came from Zhongjiang County, Deyang City, Sichuan Province. The Shandong ecotype (DSS3) was provided by the Shandong Academy of Agricultural Sciences. The Shaanxi ecotype (Shanhuang, shh) was first harvested in Shaanxi in 1999, provided by the Institute of Medicinal Plant Development of the Chinese Academy of Medical Sciences. The Henan ecotype was collected by us in Yichuan County, Nanyang City, Henan Province All materials were propagated through root segments and planted in the planting resource garden in Zhongjiang County, Sichuan Province. *A. thaliana* (col‐0) and tobacco (*N. benthamiana*) were preserved by the research team, and the mutants (*atwrky40* and *athct2*) were purchased from AraShare Biotechnology (Fuzhou, China). The environmental conditions in the plant greenhouse were 22 °C, 1500 lx, 16 h light/8 h dark. To generate *SmWRKY40* and *SmRAS* (149G and 149C), the ORF were separately cloned into pCAMBIA1300. The constructs were introduced into *A. thaliana* and hair root by *A. tumefaciens* mediated transformation.

### Determination of plant phenotype and chemical composition

Using a root scanner to analyse the root phenotype of *S. miltiorrhiza*, and measuring the root hardness using a Leeb hardness tester. Referring to the methods previously reported (Yao *et al*., 2022; Zhong *et al*., 2024), the content of tanshinones and salvianolic acid compounds in roots was analysed by high‐performance liquid chromatography, and the total lignin content in roots was determined by acetylation method.

### Decomposing analysis and histological staining

Plant tissue is embedded in paraffin or OCT, and layered specimens of plant tissue are obtained through vibration slicing or frozen slicing machines. Staining the tissue specimens with toluidine blue and safranin‐O‐fast green, and observing the microstructure of the tissue sections under a stereomicroscope. In addition, the microscopic structure of the root xylem was analysed using a scanning microscope imaging platform.

### Quantitative analysis of lignin monomer components by GC–MS

Accurately weigh 0.1 g of dry powder, add 2 mL of 4 mol/L NaOH solution, seal and hydrolyze at 95 °C for 24 h; Add 1.6 mL of 6 mol/L HCL after cooling; 13 000 r/min centrifuge for 5 min and then take 500 μL supernatant was extracted twice with 1 mL ethyl acetate. The organic phase was dried with nitrogen and dissolved in 1 mL methanol. After passing through a 0.45 μm filter membrane, it was used for GC–MS detection. The chromatographic conditions are set as follows: column box temperature of 70 °C, injection temperature of 250 °C and split ratio of 1:10; The mass spectrometry conditions are set as follows: ion source temperature of 200 °C, interface temperature of 250 °C, solvent delay of 3 min.

### Construction of a 3D model of the root, dynamic simulation of extrusion and bending deformation processes

An approximate reinforced concrete composite structure model was constructed using ABAQUS v.2022 cfinite element analysis tool (Dassault Systemes, Paris, France) (Lefèvre *et al*., 2024). Total of 17 high‐hardness strip structures (set to approximate the hardness of wood) and 1 low‐hardness material wrapped in the outer layer (set to approximate the hardness of rubber). Further simulation was conducted on the dynamic process of composite materials under different duct arrangements in response to bending and compression deformation, and the output results were plotted as pressure displacement curves. The highest point of the curve reflects the maximum stress of the composite material, and the slope of the curve reflects the viscoelasticity of the composite material.

### *De novo* sequencing, chromosome‐level assemble and comprehensive annotation of the genome of Sichuan ecotype *S. miltiorrhiza*

We extensively collect fresh tissues (including roots, stems, leaves and flowers) and immediately freeze them in liquid nitrogen for subsequent DNA and RNA isolation. Further construction of a DNA library exceeding 20 kb was carried out for single‐molecule sequencing, using PacBio Sequel platforms (Menlo Park, CA) to generate sequencing depth covering at least 100× of SMRT data. At the same time, a terminal Illumina strategy covering 60× and reading up to 350 base pairs was carried out, and paired libraries with insertion sizes of 5, 10 and 20 kb were constructed. Illumina sequencing was generated using the HiSeq 4000 platform (San Diego, CA). Next, we used FALCON v.0.3.0 (Chin *et al*., 2016) to perform the PacBio assembly following the recommended pipeline. Contigs generated by Illumina sequencing were polished using SMART Quiver v.2.3.9 (Chin *et al*., 2013) to obtain a preliminary assembly version. To eliminate possible genomic heterozygosity caused by cross‐pollination, we also used Purge Haplotigs v.2.26.0 (Roach *et al*., 2018) to optimize the assembly process. CEGMA v.2.5 (Parra *et al*., 2007) and BUSCO v5.2.2 (Simão *et al*., 2015) were used to evaluate the integrity of genome assembly.

To construct a Hi‐C library, the cell nucleus was fixed with paraformaldehyde, digested and labelled with biotin. Then cut the cross‐linked DNA using restriction endonucleases. For end repair, add biotin to label the oligonucleotide end. Connect adjacent chromatin DNA fragments using T4 DNA ligase. Finally, protease is added to digest T4 DNA ligase, allowing histones and DNA to be cross‐linked. After extracting genomic DNA, it is randomly broken into short fragments of 350 bp. Capture biotin‐labelled DNA molecules using affinity magnetic beads embedded with specific antibodies, followed by end repair, addition of poly A tail, adapter connection, PCR amplification and template purification. The constructed library was sequenced on the Illumina HiSeq PE150 platform. The analysis of Hi‐C data referred to previous reports (Pan *et al*., 2023).

Annotate duplicate components based on homologous alignment and *de novo* search (Price *et al*., 2005). Homology prediction is performed by comparing the Repbase database with genome assembly using RepeatMask (http://www.repeatmasker.org/). Extracting tandem repeat sequences from genome assembly using TRF (http://tandem.bu.edu/trf/trf.html). Using LTR_ FINDER (Price *et al*., 2005), RepeatScou and Repeat Modeller construct a database of repeating elements from scratch by predicting from scratch. All repeat sequences with a length greater than 100 bp and a gap ‘N’ <5% are used to construct the original transposable element (TE) library. The Repbase and TE libraries are processed by UCLUST (Edgar, 2010) to generate non‐redundant libraries, which are then handed over to RepeatMask for duplicate identification. Functional gene annotations mainly refer to the databases of NCBI non‐redundant protein database (Machanick and Bailey, 2011), Blast2GO (Conesa and Götz, 2008), KEGG (Ogata *et al*., 1999), InterPro (Hunter *et al*., 2009) and KOG (Koonin *et al*., 2004).

### Genomic evolution analysis

The release and integration of a large‐scale plant pan‐genome database (plantGIR, http://plantgir.cn/) have provided the possibility for comparative genome analysis (Liu *et al*., 2024a). We collected reference genomes from five other representative species of the Lamiaceae family: *Sm.SX* (Pan *et al*., 2023), *Sm.SD* (Song *et al*., 2020), *Salvia bowleyana* (Zheng *et al*., 2021), *Scutellaria baicalensis* (Wang *et al*., 2022) and *Salvia europaea* (Xu *et al*., 2020). Next, six sets of orthologous single‐copy gene sets of genomes were obtained through Orthofinder v2.5.4 (Emms and Kelly, 2019), and phylogenetic trees of six species were constructed based on this, reflecting the genetic distance between genomes through the topological structure. In addition, CAFE v.5.2.1 (Mendes and Vanderpool, 2021) was used to perform expansion/contraction analysis on gene families in 6 sets of genomes, and the differentiation time of the *S. miltiorrhiza* genome was estimated and corrected based on the known species divergence years in the Timetree (http://www.timetree.org/) (Kumar *et al*., 2022) and Paleobiodb (https://www.paleobiodb.org/) (Benton *et al*., 2015) database. Genomic collinearity analysis is achieved through MCScanX (Wang *et al*., 2012).

### Population variation analysis based on resequencing

A total of 30 representative individuals with a gradient distribution pattern of phenolic acid content were collected (10 high phenolic acid accumulations, 15 medium phenolic acid accumulations and 5 low phenolic acid accumulations). Leaf DNA was extracted and used to construct an Illumina paid end library (500 bp insert size). High‐quality reads with an average sequencing depth exceeding 10× generated for subsequent analysis. BWA‐MEM v.0.7.17 (Li and Durbin, 2009) was used to map filtered high‐quality reads to the reference genome of *S. miltiorrhiza*, while SAMtools v.1.17 (Li *et al*., 2009) was used to read segment replies, sequence sort and remove redundancy. Identify SNPs under the optimal parameters of GATK HaplotypeCaller v.4.1.4.1 (McKenna *et al*., 2010) and annotate SNPs to corresponding loci in the reference genome via ANNOVAR v.2019.10.24 (Wang *et al*., 2010). Based on PopGenome v.2020 (Pfeifer *et al*., 2014), genetic differentiation (*F*st) between *Sm.SC* and *Sm.SD* was detected within a window size of 200 kb and a step size range of 10 kb. The window with the top 5% highest *F*st ranking was set as the interval most affected by domestication selection, and further research was conducted on the candidate genes included.

### Metabolomics analysis

Accurately weigh 50 mg of freeze‐dried sample powder, add 1 mL of 80% methanol solution pre‐cooled at −20 °C, extract by vortex oscillation and centrifuge at 12,000 rpm for 5 min, and pass the supernatant through 0.22 μM membrane. The prepared extract was used for UPLC‐MS/MS detection. Ultra‐performance liquid chromatography (UPLC) uses the Agilent SB‐C18 column, ultrapure water and acetonitrile solution as the mobile phase, controlling the flow rate at 0.35 mL/min, column temperature at 40 °C and injection volume of 4 μL. The tandem mass (MS/MS) spectrometry uses an electric spray ion source, with a set temperature of 550 °C and an ion spray voltage of 5, 500 V (positive ion)/‐4, 500 V (negative ion). The MRM mode is selected for QQQ scanning. The raw data is analysed qualitatively and quantitatively based on the MWDB (Metaware database) metabolite database.

### Transcriptomic analysis

Take out the root sample of *S. miltiorrhiza* from Sichuan and Shandong and quickly freeze them in liquid nitrogen for 5 min, and then freeze the sample in a refrigerator at −80 °C for future experiments. Total RNA was extracted from each sample using the Tiangen RNA Pure kit for plants (Tiangen, Beijing, China). The cDNA libraries were constructed according to the manufacturer's instructions of the NEB Next Ultra RNA Library Prep Kit for Illumina (New England Biolabs, Ipswich). Total cDNA libraries were sequenced on the Illumina HiSeq 2500 platform (Illumina, Inc., San Diego). RNA‐seq was performed by Metware Biotechnology Co., Ltd. (Wuhan, China).

### Regulatory network inference

We employed an integrated‐omics model to depict the potential phenolic acid regulatory network in RNA‐seq and metabolic profiles. The detailed calculation pipeline was based on our previous report (Liu *et al*., 2024b,c). Specifically, we imported the normalized gene expression and metabolite accumulation matrices into the R package imsbInfer on GitHub to generate potential regulatory relationships. Based on Pearson correlation calculation (with a screening threshold of *P* < 0.01, and |cor| > 0.9), combined with known upstream and downstream relationships (especially the topological structure centred on transcription factors), Cytoscape v.3.7.2. was used to visualize the biological network involved in phenolic acid metabolism regulation. The size of nodes reflects the network path, while the colour and width of edges reflect the cor and *P* values.

### cDNA cloning and vector construction

Using the total RNA of *S. miltiorrhiza* as a template, cDNA libraries were prepared by reverse transcriptase. After adding cloned primers with enzyme cleavage sites, the ORF of the target gene was amplified by PCR. UBQ10‐*WRKY40*‐GFP and UBQ10‐*RAS*‐GFP vectors were constructed for hairy root induction and transgenic *Arabidopsis* preparation. The protein expression tool used in the enzymatic experiments for RAS is a pET28a vector with a 6 × His tag fused to the N‐terminus, recombinant plasmid transfer into *E. coli* DH5α After coating on LB solid containing 50 mg/L kanamycin. The detailed cloning primers and restriction site information are as follows: F‐*WRKY40*‐*Kpn* I: 5′‐**TAGGTACC**ATGGAGTTCACAAGCCT‐3′, R‐*WRKY40*‐*BamH* I: 5′‐**AAGGATCC**GCATTTGTCCGTGT‐3′. F‐*RAS*‐*Kpn* I: **TAGGTACC**ATGAAGATCGATATCACAGA, R‐*RAS*‐*BamH* I: **AAGGATCC**AATATCATAAAACAACTTCT. The multiple cloning site (MCS) of the pCAMBIA1305 vector was selected in the segment between downstream of the UBQ10 promoter and upstream of the GFP‐tag, and the MCS of the pET28a vector was located in the segment between the T7 promoter and the HA termination signal.

### Subcellular localization experiment

Import the target gene vector fused with GFP into *Agrobacterium* GV3101 and culture in liquid YEB medium until OD_600_ = 0.6. Add equal volume half‐strength MS liquid medium with 200 μM acetosyringone (AS) and 0.5 mM MgCl_2_, mix and annotate *Agrobacterium* containing target genes and organelle markers onto the back of mature tobacco leaves, incubate in dark for 1 day and light for 1 day. After that, place the leaf tissue under a confocal laser microscope (Olympus, FV1000, Japan) for imaging.

### Hairy root transgenic and gene editing system

Referring to the previous method (Zhong *et al*., 2024), the plant expression vector and the CRISPR‐Cas9 vector fused with the target gene gRNA were introduced into *Agrobacterium* C58C1. Cut leaves from sterile seedlings of *S. miltiorrhiza* as explants, and use C58C1 to infect the scratched leaves. The infected leaves were first placed on half‐strength MS medium and incubated in the dark for 2 days. After washing with sterile water three times, they were transferred to a half‐strength MS solid medium containing 0.5 mg/L IBA and 600 mg/L carboxybenzyl penicillin. After the hairy roots grow to 5 cm, transfer to a liquid 6,7‐V medium containing 0.5 mg/L IBA and 600 mg/L carboxybenzyl penicillin for suspension culture. When the hairy roots develop to a volume exceeding 2/3 of the tissue culture bottle, they are removed for subsequent research.

### Mutant identification and genetic transformation of Arabidopsis

The Arabidopsis mutants used in this study were prepared through T‐DNA insertion, and the genomic DNA of the tested plants was amplified using a universal T‐DNA backbone primer BP and a target gene‐specific end primer LP/RP in different combinations. The positive homozygous mutants were screened by agarose gel electrophoresis according to the band size. Transgenic Arabidopsis was obtained by infecting inflorescences with *Agrobacterium* GV3101. Positive plants were screened on a half‐strength MS medium with 25 mg/L Hyg, and genomic DNA was extracted and target genes were amplified to identify the positive transformed plants.

### DNA affinity purification sequencing (DAP‐seq)

For the DAP‐seq input library, genomic DNA was extracted from *Sm.SC* and *Sm.SD* leaves. The SmWRKY40 protein is produced using an *in vitro* transcription/translation system (Promeg Biotechnology Co., Ltd. Madison). DAP‐seq was based on the published protocol with slight modifications (Bartlett *et al*., 2017), DNA fragments captured by immunoprecipitation will be subjected to 20 cycles of linear PCR amplification and purified using AMPure XP magnetic beads (Beckman Coulter, Inc. Brea). NGS sequencing was performed using the Illumina platform, and Bioinformatics analysis was done after refinement based on the published protocols (Yu *et al*., 2015). Two biological replicates and one Input were set as controls in the DAP‐seq experiment.

### Dual‐Luciferase reporter assay, yeast one hybrid, transcriptional activation activity and EMSA manual

We constructed the pGreenII‐62SK‐*WRKY40* vector and the pGreenII‐0800 LUC vector inserted into the *RAS* and *HCT* promoters to verify the interaction in the tobacco expression system. The tobacco expression system and the specific test methods are as described above. The interaction of the transcription factor with the promoter was verified by comparison of fluorescence intensity.

The ORF of *SmWRKY40* was constructed into the multiple cloning sites (MCS) of the pGADT7 vector, and the promoter region 1 kb upstream of the RAS and HCT genes was constructed into the MCS of pHIS2. The combined plasmids were co‐transformed into *S. cerevisiae* (Y187), and positive transformants were screened in SD‐Trp/‐Leu plates and interactions in SD‐Trp/‐Leu/‐His plates. In addition, we constructed the ORF of *SmWRKY40* onto the pGBKT7 vector, transferred it into yeast Y187 and coated it on SD/‐Trp/‐His+x‐α‐gal medium, the transcriptional activation activity of transcription factors was determined through blue and white spot screening.

Construct the ORF of *SmWRKY40* into the prokaryotic expression vector pET‐28a, and introduce it into *E. coli* BL21, induce it with 0.1 mM IPTG at 16 °C for 20 h and obtain the recombinant protein through the Ni‐IDA purification column. A biotin‐labelled probe was synthesized for the DNA sequence rich in W‐box elements on the promoter of the target gene. The probe was incubated with SmWRKY40 protein under suitable conditions, and then SDS‐PAGE and autoradiography were used to determine whether the protein interacted with DNA.

### *In vitro* enzyme kinetics experiment

Recombinant proteins for RAS^146C^ and RAS^146G^, and homologues from sweet orange were produced in *E. coli*. Referring to the previous method (Zhong *et al*., 2024), the relevant coding sequences were cloned into the pET‐28a expression vector in‐frame and downstream of the sequence of the 6 × His tag using a Clon Express MultiS One Step Cloning Kit (Vazyme Biotech Co., Ltd, Nanjing, China). Transform the recombinant vector into *E. coli* BL21 (DE3) for heterologous protein production. Select a single colony and culture it in LB liquid medium containing kanamycin until OD_600_ = 0.6–0.8, then add isopropyl thiol at 16 °C, isopropylthio‐β‐galactoside (IPTG, final concentration 0.1 mM) was induced for 20 h. Harvest cells (6000 × ***g***. 10 min at 4 °C) and crush them with an ultrasonic crusher. Centrifugation (14,000 × ***g***. 15 min, 4 °C) to remove cell debris. Purify the target protein using Ni‐IDA pre‐installed gravity column and confirm it by SDS‐PAGE electrophoresis.

For acyltransferase, in a total volume of 100 μL *in vitro* enzymatic assay was performed under the condition (Meyermans *et al*., 2000; Zhou *et al*., 2024), 150 μM Acyl donor with 50 μM acyl receptor, 5 mM MgCl_2_ and 500 ng purified protein (buffer containing 100 mM Tris–HCl buffer and 0.1% glycerol, pH 8.0). After incubating at 25 °C for 2 h, add 10 μL cold methanol to stop reacting. Then the reaction mixture through 0.2 μM filter filtration, was used for subsequent LC–MS analysis. Each reaction undergoes three independent experimental replicates and three technical replicates. In this work, caffeoyl‐CoA was used as an acyl donor, while danshensu and quinic acid were used as acyl receptors. All HPLC‐grade purity chemicals are purchased from Shanghai Yuanye Bio‐Technology Co., Ltd. (Shanghai, China).

### Stress response and physiological detection

Plant stress experiments were conducted using SmWRKY40 transgenic *Arabidopsis* and mutant complement lines, specifically by adding 100 μM CuSO_4_ to half‐strength MS solid culture medium or nutrient soil to simulate Cu^2+^ toxicity (process for 2 weeks), culturing plants in a 12 °C environment (process for 2 weeks) to simulate cold stress. Plants are exposed to a shelf 60 cm away from UV‐B lamps (6 h/day, for 2 weeks) to simulate high doses of UV‐B stress. The experimental groups are compared with Arabidopsis cultivated under normal nutrition, light and temperature conditions. The experimental methods for yeast are different. The ORF of SmWRKY40 and RAS were constructed into the polyclonal site of the pYES2 expression vector, and the recombinant vector was introduced into the stress‐sensitive yeast strain INVSC1. Positive transformants were screened on SD medium with Ura deficiency. Corresponding stress treatments were added during the subsequent solid plate culture and liquid culture processes. Referring to previous reports (Yu *et al*., 2023), the content of carotenoids, chlorophyll, MDA, O_2_^−^ and CAT enzyme activity in organisms under stress was measured using a kit produced by Nanjing Jiancheng Biotechnology Co., Ltd (Nanjing, China).

### Real‐time quantitative PCR (RT‐qPCR) detection

The primer sequences with measured genes were designed through the Primer 3 (https://primer3.ut.ee/). The RT‐qPCR conditions consisted of pre‐denaturation at 95 °C for 3 min, followed by 40 cycles of denaturation at 95 °C for 5 s and annealing and extension at 60 °C for 30 s. The relative expression of genes was calculated with the 2^−ΔΔct^ method (Livak and Schmittgen, 2001), and the *SmACTIN* gene was selected as the internal reference gene. All experiments were set up with 3 biological replicates and 3 technical replicates.

### Quantification and statistical analysis

All data in this study were analysed using the Origin v.2020 and SPSS statistics program by two‐tailed Student's *t*‐test (*P < 0.05 and **P < 0.01), and the means were compared by the least significant difference (LSD) test at the 0.05 level of significance.

## Author contributions

Haomiao Yu: Designed this study, carried out genetics and physiological experiments, integrated‐omics data analysis and manuscript writing. Jinqiu Liao and Yuanyuan Jiang: Germplasm resources collection, phenotypic and biochemical analyses and experimental data analysis. Mingzhi Zhong and Shan Tao: Construction of cloning vectors, establishment of a prokaryotic expression system, protein induction and purification and enzyme kinetic analysis. Songyue Chai, Long Wang and Li Lin: Genome, transcriptomic and metabolomics analyses. Ruiwu Yang, Xuexue Deng and Yunsong Zhang: Genetic and physiological analysis. Xiang Pu: Designed and directed biochemical experiments on protein induction, isolation and purification and enzyme kinetics. Moyang Liu: Designed the multi‐omics framework for the whole study, guided the bioinformatics analysis, provided a high‐performance computing platform and reviewed and revised the manuscript. Li Zhang: Led the whole study, provided the experimental platform and funding support.

## Conflict of interest

The authors declare no competing interests.

## Supporting information


**Appendix S1** Supplementary table S1‐S14.

**Figure S1** Meteorological map of environmental meteorological factors in different *S. miltiorrhiza* producing areas.
**Figure S2** Phenotype and composition detection of *S. miltiorrhiza* root under UV‐B exposure, cold stress and Cu^2+^ stress.
**Figure S3** 3D modeling and stress simulation of the effect of different xylem arrangement on mechanical strength of roots.
**Figure S4** Comparative metabolome analysis of *Sm*.SC and *Sm*.SD mature roots.
**Figure S5** Statistics of whole genome sequencing and chromosome mount information.
**Figure S6** Comparative transcriptomic analysis of *Sm*.SC and *Sm*.SD mature roots.
**Figure S7** Transcription factor‐metabolite association analysis of phenolic acid metabolic pathways.
**Figure S8** Cross‐species evolutionary analysis of the *WRKY40*‐*CCR*‐*HCT* metabolic cluster.
**Figure S9** Subcellular localization and expression pattern of SmWRKY40.
**Figure S10** Preparation flow of transgenic/gene‐edited hairy roots.
**Figure S11** qPCR analysis of phenolic acid metabolism pathway genes in hairy roots and hair root.
**Figure S12** Identification of *Arabidopsis* mutants and transgenic plants.
**Figure S13** SmWRKY40 binding peaks on promoters of other pathway genes.
**Figure S14** Comparison of the binding of WRKY40 transcription factors to the promoters of the *RAS* and *HCT2* genes.
**Figure S15** Haplotype comparison of rosmarinic acid synthase (RAS) from *Sm.SC* and *Sm.SD*.
**Figure S16** Association analysis of RA and SAB content with haplotypes and acyltransferase activity in population samples, and groping of reaction conditions of rosmarinic acid synthase (RAS) from *Sm.SC* and *Sm.SD*.
**Figure S17** Phylogeny and multiple sequence alignment of *SmRAS* and homologous sequences.
**Figure S18** Identification of *Arabidopsis* mutants and transgenic plants.
**Figure S19** Heterologous expression validates the biological functions of *SmWRKY40* and *SmRASs* in response to cold stress.
**Figure S20** Heterologous expression validates the biological functions of *SmWRKY40* and *SmRASs* in response to Cu^2+^ stress.

**Table S1** Basic information on germplasm resources of *S. miltiorrhiza* with different ecotypes.
**Table S2** Investigation of the meteorological environment in different *S. miltiorrhiza* production regions.
**Table S3** Comparative metabolomic analysis of *Sm.SC* and *Sm.SD*.
**Table S4** Statistics of the whole‐genome sequencing data based on the PacBio platform.
**Table S5** Hi‐C assisted assembly data statistics.
**Table S6** Genomic orthologous identification of *S. miltiorrhiza* and its related species.
**Table S7** Comparative transcriptomics analysis of *Sm.SC* and *Sm.SD*.
**Table S8** Comparative analysis of key enzyme genes and metabolites in the phenolic acid metabolic pathway in *Sm.SC* and *Sm.SD*.
**Table S9** Gene‐metabolite regulatory network of phenolic acid metabolism pathway constructed based on integrative omics analysis.
**Table S10** Data statistics for population resequencing of *Sm.SC* and *Sm.SD*.
**Table S11** Single nucleotide polymorphism (SNP) statistics on HIC_ASM_6 in *Sm.SC* and *Sm.SD* population resequencing.
**Table S12** Statistics of large segment variation (Indel) located on HIC_ASM_6 in *Sm.SC* and *Sm.SD* population resequencing.
**Table S13** Population differentiation (*F*st) statistics of *Sm.SC* and *Sm.SD* on HIC_ASM_6.
**Table S14** Statistics of the peaks of interaction with SmWRKY40 in phenolic acid metabolism clusters of *Sm.SC* and *Sm.SD* based on DAP‐seq.

## Data Availability

The genome sequencing, RNA‐seq and DAP‐seq data performed in this study have been deposited in the NCBI (https://www.ncbi.nlm.nih.gov/bioproject/, PRJNA862689, SAMN29989663), National Genomics Data Center (https://ngdc.cncb.ac.cn/, PRJCA010426, PRJCA023817) and can be accessed via the corresponding author if necessary.

## References

[pbi70048-bib-0001] Bartlett, A. , O'Malley, R. , Huang, S. , Galli, M. , Nery, J. , Gallavotti, A. and Ecker, J. (2017) Mapping genome‐wide transcription‐factor binding sites using DAP‐seq. Nat. Protoc. 12, 1659–1672. 28726847 10.1038/nprot.2017.055 PMC5576341

[pbi70048-bib-0002] Benton, M. , Donoghue, P. , Friedman, R. , Vinther, T. , Asher, R. , Friedman, M. and Vinther, J. (2015) Constraints on the timescale of animal evolutionary history. Palaeontol. Electron. 18, 1–16.

[pbi70048-bib-0003] Cao, R. , Lv, B. , Shao, S. , Zhao, Y. , Yang, M. , Zuo, A. , Wei, J. *et al*. (2024) The SmMYC2‐SmMYB36 complex is involved in methyl jasmonate‐mediated tanshinones biosynthesis in *Salvia miltiorrhiza* . Plant J. 119, 746–761. 38733631 10.1111/tpj.16793

[pbi70048-bib-0004] Che, Y. , Sun, Y. , Lu, S. , Zhao, F. , Hou, L. and Liu, X. (2018) AtWRKY40 functions in drought stress response in *Arabidopsis thaliana* . Plant Physiol J. 54, 456–464.

[pbi70048-bib-0005] Chen, J. , Wang, Y. , Di, P. , Wu, Y. , Qiu, S. , Lv, Z. , Qiao, Y. *et al*. (2023) Phenotyping of *Salvia miltiorrhiza* roots reveals associations between root traits and bioactive components. Plant Phenom. 5, 98. 10.34133/plantphenomics.0098 PMC10545446 37791248

[pbi70048-bib-0006] Chin, C. , Alexander, D. , Marks, P. , Klammer, A. , Drake, J. , Heiner, C. , Clum, A. *et al*. (2013) Nonhybrid, finished microbial genome assemblies from long‐read SMRT sequencing data. Nat. Methods, 10, 563–569. 23644548 10.1038/nmeth.2474

[pbi70048-bib-0007] Chin, C.S. , Peluso, P. and Sedlazeck, F.J. (2016) Phased diploid genome assembly with single‐molecule real‐time sequencing. Nat. Methods, 13, 1050–1054. 27749838 10.1038/nmeth.4035 PMC5503144

[pbi70048-bib-0008] Conesa, A. and Götz, S. (2008) Blast2GO: a comprehensive suite for functional analysis in plant genomics. Int. J. Plant Genomics, 208, 619832. 10.1155/2008/619832 PMC2375974 18483572

[pbi70048-bib-0009] Deng, C. , Shi, M. , Fu, R. , Zhang, Y. , Wang, Q. , Zhou, Y. , Wang, Y. *et al*. (2020) An ABA‐responsive SmbZIP1 is involved in modulating biosynthesis of phenolic acids and tanshinones in *Salvia miltiorrhiza* . J. Exp. Bot. 71, 1–15. 32589719 10.1093/jxb/eraa295

[pbi70048-bib-0010] Edgar, R.C. (2010) Search and clustering orders of magnitude faster than BLAST. Bioinformatics, 26, 2460–2461. 20709691 10.1093/bioinformatics/btq461

[pbi70048-bib-0011] Emms, D.M. and Kelly, S. (2019) OrthoFinder: phylogenetic orthology inference for comparative genomics. Genome Biol. 20, 238. 31727128 10.1186/s13059-019-1832-y PMC6857279

[pbi70048-bib-0012] Fan, P. , Wu, L. , Wang, Q. , Wang, Y. , Luo, H. , Song, J. , Yang, M. *et al*. (2023) Physiological and molecular mechanisms of medicinal plants in response to cadmium stress: current status and future perspective. J. Hazard. Mater. 450, 131008. 36842201 10.1016/j.jhazmat.2023.131008

[pbi70048-bib-0013] Guo, L. , Yao, H. , Chen, W. , Wang, X. , Ye, P. , Xu, Z. , Zhang, S. *et al*. (2022) Natural products of medicinal plants: biosynthesis and bioengineering in post‐genomic era. Hortic. Res. 9, uhac223. 36479585 10.1093/hr/uhac223 PMC9720450

[pbi70048-bib-0014] Huang, Q. , Sun, M. , Yuan, T. , Wang, Y. , Shi, M. , Lu, S. , Tang, B. *et al*. (2018) The AP2/ERF transcription factor SmERF1L1 regulates the biosynthesis of tanshinones and phenolic acids in *Salvia miltiorrhiza* . Food Chem. 274, 368–375. 30372953 10.1016/j.foodchem.2018.08.119

[pbi70048-bib-0015] Hunter, S. , Apweiler, R. , Attwood, T. , Bairoch, A. , Bateman, A. , Binns, D. , Bork, P. *et al*. (2009) InterPro: the integrative protein signature database. Nucleic Acids Res. 37, D211–D215. 18940856 10.1093/nar/gkn785 PMC2686546

[pbi70048-bib-0016] Jia, E. , Li, H. , He, F. , Xu, X. , Wei, J. , Shao, G. , Liu, J. *et al*. (2024) Metabolic engineering of artificially modified transcription factor SmMYB36‐VP16 for high‐level production of tanshinones and phenolic acids. Metab. Eng. 86, 29–40. 39181435 10.1016/j.ymben.2024.08.004

[pbi70048-bib-0017] Koonin, E. , Fedorova, N. , Jackson, J. , Jacobs, A. , Krylov, D. , Makarova, K. , Mazumder, R. *et al*. (2004) A comprehensive evolutionary classification of proteins encoded in complete eukaryotic genomes. Genome Biol. 5, R7. 14759257 10.1186/gb-2004-5-2-r7 PMC395751

[pbi70048-bib-0018] Kruse, L. , Weigle, A. , Irfan, M. , Martínez‐Gómez, J. , Chobirko, J. , Schaffer, J. , Bennett, A. *et al*. (2022) Orthology‐based analysis helps map evolutionary diversification and predict substrate class use of BAHD acyltransferases. Plant J. 111, 1453–1468. 35816116 10.1111/tpj.15902

[pbi70048-bib-0019] Kruse, L. , Fehr, B. , Chobirko, J. and Moghe, G. (2023) Phylogenomic analyses across land plants reveals motifs and coexpression patterns useful for functional prediction in the BAHD acyltransferase family. Front. Plant Sci. 14, 1067613. 36844084 10.3389/fpls.2023.1067613 PMC9950517

[pbi70048-bib-0020] Kumar, S. , Suleski, M. and Craig, J.M. (2022) TimeTree 5: an expanded resource for species divergence times. Mol. Biol. Evol. 39, msac174. 35932227 10.1093/molbev/msac174 PMC9400175

[pbi70048-bib-0021] Lefèvre, V. , Sozio, F. and Lopez‐Pamies, O. (2024) Abaqus implementation of a large family of finite viscoelasticity models. Finite Elem. Anal. Des. 232, 104114.

[pbi70048-bib-0022] Li, H. and Durbin, R. (2009) Fast and accurate short read alignment with Burrows‐Wheeler transform. Bioinformatics, 25, 1754–1760. 19451168 10.1093/bioinformatics/btp324 PMC2705234

[pbi70048-bib-0023] Li, H. , Handsaker, B. , Wysoker, A. , Fennell, T. , Ruan, J. , Homer, N. , Marth, G. *et al*. (2009) The sequence alignment/map format and SAMtools. Bioinformatics, 25, 2078–2079. 19505943 10.1093/bioinformatics/btp352 PMC2723002

[pbi70048-bib-0024] Li, X. , Park, S. , Jin, F. , Deng, Y. , Yang, J. , Chang, J. , Kim, D. *et al*. (2018) Tanshinone IIA suppresses FcεRI‐mediated mast cell signaling and anaphylaxis by activation of the Sirt1/LKB1/AMPK pathway. Biochem. Pharmacol. 152, 362–372. 29674003 10.1016/j.bcp.2018.04.015

[pbi70048-bib-0025] Li, Y. , Kong, D. , Fu, Y. , Sussman, M. and Wu, H. (2020a) The effect of developmental and environmental factors on secondary metabolites in medicinal plants. Plant Physiol. Biochem. 148, 80–89. 31951944 10.1016/j.plaphy.2020.01.006

[pbi70048-bib-0026] Li, Z. , Zou, J. , Cao, D. and Ma, X. (2020b) Pharmacological basis of tanshinone and new insights into tanshinone as a multitarget natural product for multifaceted diseases. Biomed. Pharmacother. 130, 110599. 33236719 10.1016/j.biopha.2020.110599

[pbi70048-bib-0027] Li, Q. , Fang, X. , Zhao, Y. , Cao, R. , Dong, J. and Ma, P. (2023) The SmMYB36‐SmERF6/SmERF115 module regulates the biosynthesis of tanshinones and phenolic acids in *Salvia miltiorrhiza* hairy roots. Hortic. Res. 10, uhac238. 36643739 10.1093/hr/uhac238 PMC9832864

[pbi70048-bib-0028] Li, M. , Shao, Y. , Pan, B. , Liu, C. and Tan, H. (2025a) Regulation of important natural products biosynthesis by WRKY transcription factors in plants. J. Adv. Res. 1, 1–17.10.1016/j.jare.2025.01.00939761870

[pbi70048-bib-0029] Li, Q. , Wang, X. , Wang, J. , Su, Y. , Guo, Y. , Yang, J. , Liu, J. *et al*. (2025b) SmCSN5 is a synergist in the transcription factor SmMYB36 mediated tanshinones and phenolic acids biosynthesis in *Salvia miltiorrhiza* . Hortic. Res. 1, uhaf005. 10.1093/hr/uhaf005 PMC11896976 40078719

[pbi70048-bib-0030] Liang, H. , Kong, Y. , Chen, W. , Wang, X. , Jia, Z. , Dai, Y. and Yang, X. (2020) The quality of wild *Salvia miltiorrhiza* from Dao Di area in China and its correlation with soil parameters and climate factors. Phytochem. Anal. 32, 318–325. 32761717 10.1002/pca.2978

[pbi70048-bib-0032] Liu, S. , Wang, Y. , Shi, M. , Maoz, I. , Gao, X. , Sun, M. , Yuan, T. *et al*. (2022) SmbHLH60 and SmMYC2 antagonistically regulate phenolic acids and anthocyanins biosynthesis in *Salvia miltiorrhiza* . J. Adv. Res. 42, 205–219. 36513414 10.1016/j.jare.2022.02.005 PMC9788942

[pbi70048-bib-0033] Liu, Z. , Zhang, C. , He, J. , Li, C. , Fu, Y. , Zhou, Y. , Cao, R. *et al*. (2024a) plantGIR: a genomic database of plants. Hortic. Res. 11, uhae342. 39712867 10.1093/hr/uhae342 PMC11661351

[pbi70048-bib-0034] Liu, M. , Yang, M. , Liang, H. , Luo, B. , Deng, J. , Cao, L. , Zheng, D. *et al*. (2024b) Polyploidy drives autophagy to participate in plant‐specific functions. iMeta, 3, e252. 39742296 10.1002/imt2.252 PMC11683458

[pbi70048-bib-0035] Liu, M. , Yu, J. , Yang, M. , Cao, L. and Chen, C. (2024c) Adaptive evolution of chloroplast division mechanisms during plant terrestrialization. Cell Rep. 43, 113950. 38489264 10.1016/j.celrep.2024.113950

[pbi70048-bib-0036] Livak, K.J. and Schmittgen, T.D. (2001) Analysis of relative gene expression data using real‐time quantitative PCR and the 2(‐Delta Delta C(T)) method. Methods, 25, 402–408. 11846609 10.1006/meth.2001.1262

[pbi70048-bib-0037] Lv, B. , Deng, H. , Wei, J. , Feng, Q. , Liu, B. , Zuo, A. , Bai, Y. *et al*. (2024) SmJAZs‐SmbHLH37/SmERF73‐SmSAP4 module mediates jasmonic acid signaling to balance biosynthesis of medicinal metabolites and salt tolerance in *Salvia miltiorrhiza* . New Phytol. 244, 1450–1466. 39262232 10.1111/nph.20110

[pbi70048-bib-0038] Ma, R. , Wenxuan, H. , Hu, Q. , Tian, G. , An, J. , Fang, T. , Liu, J. *et al*. (2024) Tandemly duplicated MYB genes are functionally diverged in the regulation of anthocyanin biosynthesis in soybean. Plant Physiol. 194, 2549–2563. 38235827 10.1093/plphys/kiae019

[pbi70048-bib-0039] Machanick, P. and Bailey, T.L. (2011) MEME‐ChIP: motif analysis of large DNA datasets. Bioinformatics, 27, 1696–1697. 21486936 10.1093/bioinformatics/btr189 PMC3106185

[pbi70048-bib-0040] McKenna, A. , Hanna, M. , Banks, E. , Sivachenko, A. , Cibulskis, K. , Kernytsky, A. , Garimella, K. *et al*. (2010) The Genome Analysis Toolkit: a MapReduce framework for analyzing next‐generation DNA sequencing data. Genome Res. 20, 1297–1303. 20644199 10.1101/gr.107524.110 PMC2928508

[pbi70048-bib-0041] Mendes, F.K. and Vanderpool, D. (2021) CAFE 5 models variation in evolutionary rates among gene families. Bioinformatics, 36, 5516–5518. 33325502 10.1093/bioinformatics/btaa1022

[pbi70048-bib-0042] Meyermans, H. , Morreel, K. , Lapierre, C. , Pollet, B. , De Bruyn, A. , Busson, R. , Herdewijn, P. *et al*. (2000) Modifications in lignin and accumulation of phenolic glucosides in poplar xylem upon down‐regulation of caffeoyl‐coenzyme A O‐methyltransferase, an enzyme involved in lignin biosynthesis. J. Biol. Chem. 275, 36899–36909. 10934215 10.1074/jbc.M006915200

[pbi70048-bib-0043] Morreel, K. , Ralph, J. , Kim, H. , Lu, F. , Goeminne, G. , Ralph, S. , Messens, E. *et al*. (2004) Profiling of oligolignols reveals monolignol coupling conditions in lignifying poplar xylem. Plant Physiol. 136, 3537–3549. 15516504 10.1104/pp.104.049304 PMC527153

[pbi70048-bib-0044] Ogata, H. , Goto, S. , Sato, K. , Fujibuchi, W. , Bono, H. and Kanehisa, M. (1999) KEGG: Kyoto encyclopedia of genes and genomes. Nucleic Acids Res. 27, 29–34. 9847135 10.1093/nar/27.1.29 PMC148090

[pbi70048-bib-0045] Olivier, V. , Botao, Z. , Simon, L. , Reena, N. and James, W. (2013) AtWRKY40 and AtWRKY63 modulate the expression of stress‐responsive nuclear genes encoding mitochondrial and chloroplast proteins. Plant Physiol. 162, 254–271. 23509177 10.1104/pp.113.215996 PMC3641207

[pbi70048-bib-0046] Pan, X. , Chang, Y. , Li, C. , Qiu, X. , Cui, X. , Meng, F. , Zhang, S. *et al*. (2023) Chromosome‐level genome assembly of *Salvia miltiorrhiza* with orange roots uncovers the role of Sm2OGD3 in catalyzing 15,16‐dehydrogenation of tanshinones. Hortic. Res. 10, uhad069. 37293533 10.1093/hr/uhad069 PMC10244880

[pbi70048-bib-0047] Parra, G. , Bradnam, K. and Korf, I. (2007) CEGMA: a pipeline to accurately annotate core genes in eukaryotic genomes. Bioinformatics, 23, 1061–1067. 17332020 10.1093/bioinformatics/btm071

[pbi70048-bib-0080] Pfeifer, B. , Wittelsbürger, U. , Onsins, S. , Lercher, M. (2014). Popgenome:an efficient swiss army knife for population genomic analyses in R.MolBiol Evol.31, 1929‐1936. 10.1093/molbev/msu136 PMC4069620 24739305

[pbi70048-bib-0048] Price, A.L. , Jones, N.C. and Pevzner, P.A. (2005) De novo identification of repeat families in large genomes. Bioinformatics, 21, i351–i358. 15961478 10.1093/bioinformatics/bti1018

[pbi70048-bib-0049] Qin, L. , Hu, Y. , Wang, J. , Wang, X. , Zhao, R. , Shan, H. , Li, K. *et al*. (2021) Insights into angiosperm evolution, floral development and chemical biosynthesis from the *Aristolochia fimbriata* genome. Nat. Plants, 7, 1239–1253. 34475528 10.1038/s41477-021-00990-2 PMC8445822

[pbi70048-bib-0050] Roach, M.J. , Schmidt, S.A. and Borneman, A.R. (2018) Purge Haplotigs: allelic contig reassignment for third‐gen diploid genome assemblies. BMC Bioinformatics, 19, 460. 30497373 10.1186/s12859-018-2485-7 PMC6267036

[pbi70048-bib-0051] Saha, B. , Nayak, J. , Srivastava, R. , Samal, S. , Kumar, D. , Chanwala, J. , Dey, N. *et al*. (2023) Unraveling the involvement of WRKY TFs in regulating plant disease defense signaling. Planta, 259, 7. 38012461 10.1007/s00425-023-04269-y

[pbi70048-bib-0052] Saini, M. , Capalash, N. , Varghese, E. , Kaur, C. and Singh, S. (2019) Quantitative metabolomics approach reveals dynamics of primary metabolites in ‘Kinnow’ mandarin (*C. nobilis* × *C. deliciosa*) during advanced stages of fruit maturation under contrasting growing climates. J. Hortic. Sci. Biotechnol. 95, 1–7.

[pbi70048-bib-0053] Shi, M.‐J. , Dong, B.‐S. , Yang, W.‐N. , Su, S.‐B. and Zhang, H. (2019) Preventive and therapeutic role of Tanshinone IIA in hepatology. Biomed. Pharmacother. 112, 108676. 30797157 10.1016/j.biopha.2019.108676

[pbi70048-bib-0054] Shi, M. , Zhang, S. , Zheng, Z. , Itay, M. , Zhang, L. and Kai, G. (2024) Molecular regulation of the key specialized metabolism pathways in medicinal plants. J. Integr. Plant Biol. 66, 510–531. 38441295 10.1111/jipb.13634

[pbi70048-bib-0055] Shuangqian, S. , Peng, M. , Fang, H. , Wang, Z. , Shen, Z. , Jing, X. , Zhang, M. *et al*. (2021) An oryza specific hydroxycinnamoyl tyramine gene cluster contributes to enhanced disease resistance. Sci. Bull. 66, 2369–2380. 10.1016/j.scib.2021.03.015 36654123

[pbi70048-bib-0056] Simão, F.A. , Waterhouse, R.M. , Ioannidis, P. , Kriventseva, E.V. and Zdobnov, E.M. (2015) BUSCO: assessing genome assembly and annotation completeness with single‐copy orthologs. Bioinformatics, 31, 3210–3212. 26059717 10.1093/bioinformatics/btv351

[pbi70048-bib-0057] Song, Z. , Lin, C. , Xing, P. , Fen, Y. , Jin, H. , Zhou, C. , Gu, Y.Q. *et al*. (2020) A high‐quality reference genome sequence of *Salvia miltiorrhiza* provides insights into tanshinone synthesis in its red rhizomes. Plant Genome, 13, e20041. 33217202 10.1002/tpg2.20041 PMC12807052

[pbi70048-bib-0058] Wang, K. , Li, M. and Hakonarson, H. (2010) ANNOVAR: functional annotation of genetic variants from high‐throughput sequencing data. Nucleic Acids Res. 38, e164. 20601685 10.1093/nar/gkq603 PMC2938201

[pbi70048-bib-0059] Wang, Y. , Tang, H. , Debarry, J.D. , Tan, X. , Li, J. , Wang, X. , Lee, T.H. *et al*. (2012) MCScanX: a toolkit for detection and evolutionary analysis of gene synteny and collinearity. Nucleic Acids Res. 40, e49. 22217600 10.1093/nar/gkr1293 PMC3326336

[pbi70048-bib-0060] Wang, L. , Lee, M. , Sun, F. , Song, Z. , Yang, Z. and Yue, G.H. (2022) A chromosome‐level genome assembly of chia provides insights into high omega‐3 content and coat color variation of its seeds. Plant Commun. 3, 100326. 35605203 10.1016/j.xplc.2022.100326 PMC9284293

[pbi70048-bib-0061] Wang, S. , Shuangqian, S. , Wang, C. , Wang, X. , Chenkun, Y. , Shen, Z. , Zhang, R. *et al*. (2023) A metabolomics study in citrus provides insight into bioactive phenylpropanoid metabolism. Hortic. Res. 11, uhad267. 38304332 10.1093/hr/uhad267 PMC10831325

[pbi70048-bib-0062] Wang, Z. , Ye, X. , Huang, L. and Yuan, Y. (2025) Modulation of morphogenesis and metabolism by plant cell biomechanics: from model plants to traditional herbs. Hortic. Res. 1, uhaf011. 10.1093/hr/uhaf011 PMC11908831 40093376

[pbi70048-bib-0063] Xiao, Z. , Liu, W. , Mu, Y.‐p. , Zhang, H. , Wang, X. , Zhao, C. , Chen, J. *et al*. (2020) Pharmacological effects of salvianolic acid B against oxidative damage. Front. Pharmacol. 11, 532373. 10.3389/fphar.2020.572373 PMC7741185 33343348

[pbi70048-bib-0064] Xu, H. , Song, J. , Luo, H. , Zhang, Y. , Li, Q. , Zhu, Y. , Xu, J. *et al*. (2016) Analysis of the genome sequence of the medicinal plant *Salvia miltiorrhiza* . Mol. Plant, 9, 949–952. 27018390 10.1016/j.molp.2016.03.010 PMC5517341

[pbi70048-bib-0065] Xu, Z. , Gao, R. , Pu, X. , Xu, R. , Wang, J. , Zheng, S. , Zeng, Y. *et al*. (2020) Comparative genome analysis of scutellaria baicalensis and Scutellaria barbata reveals the evolution of active flavonoid biosynthesis. Genomics Proteomics Bioinformatics, 18, 230–240. 33157301 10.1016/j.gpb.2020.06.002 PMC7801248

[pbi70048-bib-0066] Yajun, W. , Peng, H. , Shen, Y. , Zhao, R. and Huang, L. (2013) The profiling of bioactive ingredients of differently aged *Salvia miltiorrhiza* roots. Microsc. Res. Tech. 76, 947–954. 23839871 10.1002/jemt.22253

[pbi70048-bib-0067] Yan, F. (2016) Effects of salvianolic acid B on growth inhibition and apoptosis induction of ovarian cancer SKOV3. Eur. J. Gynaecol. Oncol. 37, 653–656. 29787004

[pbi70048-bib-0068] Yao, S.C. , Jiang, Y.Y. , Ni, S. , Wang, L. , Feng, J. , Yang, R.W. , Yang, L.X. *et al*. (2022) Development of a highly efficient virus‐free regeneration system of *Salvia miltiorrhiza* from Sichuan using apical meristem as explants. Plant Methods, 18, 50. 35436933 10.1186/s13007-022-00872-4 PMC9014595

[pbi70048-bib-0069] Yin, X. , Hui, F. , Chen, Y. , Lan, Z. , Wei, S. , Fan, Y. , Zhou, W. *et al*. (2020) Integrative omic and transgenic analyses reveal the positive effect of ultraviolet‐B irradiation on salvianolic acid biosynthesis through up‐regulation of SmNAC1. Plant J. 104, 781–799. 32772407 10.1111/tpj.14952

[pbi70048-bib-0070] Yu, G. , Wang, L.G. and He, Q.Y. (2015) ChIPseeker: an R/Bioconductor package for ChIP peak annotation, comparison and visualization. Bioinformatics, 31, 2382–2383. 25765347 10.1093/bioinformatics/btv145

[pbi70048-bib-0071] Yu, H. , Zhang, L. , Yang, R. , Jiang, Y. , Liao, J. , Chai, S. , Deng, X. *et al*. (2023) Integrated multiomics and synergistic functional network revealed the mechanism in the tolerance of different ecotypes of *Salvia miltiorrhiza* bge. to doxycycline pollution. Environ. Sci. Technol. 57, 9603–9614. 37342920 10.1021/acs.est.3c02908

[pbi70048-bib-0072] Zhang, F. , Chenkun, Y. , Guo, H. , Yufei, L. , Shuangqian, S. , Zhou, Q. , Li, C. *et al*. (2024a) Dissecting the genetic basis of UV‐B responsive metabolites in rice. Genome Biol. 25, 234. 39210441 10.1186/s13059-024-03372-x PMC11360312

[pbi70048-bib-0073] Zhang, L. , Tao, S. , Zhang, Y. , Yang, Y. , Peng, F. , Liao, H. , Mao, C. *et al*. (2024b) Study on the effect of compound cultivation on the growth feature and active ingredients content of *Salvia miltiorrhiza* . Front. Plant Sci. 14, 1–12. 10.3389/fpls.2023.1238896 PMC10854009 38343765

[pbi70048-bib-0074] Zhao, Y. , Liu, G. , Yang, F. , Liang, Y. , Gao, Q. , Xiang, C. , Li, X. *et al*. (2023) Multilayered regulation of secondary metabolism in medicinal plants. Mol. Hortic. 3, 11. 37789448 10.1186/s43897-023-00059-y PMC10514987

[pbi70048-bib-0075] Zheng, X. , Chen, D. , Chen, B. , Liang, L. , Huang, Z. , Fan, W. , Chen, J. *et al*. (2021) Insights into salvianolic acid B biosynthesis from chromosome‐scale assembly of the *Salvia bowleyana* genome. J. Integr. Plant Biol. 63, 1309–1323. 33634943 10.1111/jipb.13085

[pbi70048-bib-0076] Zhong, M. , Zhang, L. , Yu, H. , Liao, J. , Jiang, Y. , Chai, S. , Yang, R. *et al*. (2024) Identification and characterization of a novel tyrosine aminotransferase gene (SmTAT3‐2) promotes the biosynthesis of phenolic acids in *Salvia miltiorrhiza* Bunge. Int. J. Biol. Macromol. 254, 127858. 37924917 10.1016/j.ijbiomac.2023.127858

[pbi70048-bib-0077] Zhou, D. , Tian, T. , Shu, Q. , Zhao, X.Y. , Ma, W. , Zhang, J.H. , Fan, L.X. *et al*. (2018) Effect of salvianolic acid B on angiogenesis of myocardial ischemia in rats. Chin. Tradit. Herb. Drug, 49, 5166–5169.

[pbi70048-bib-0078] Zhou, W. , Shi, M. , Deng, C. , Lu, S. , Huang, F. , Wang, Y. and Kai, G. (2021) The methyl jasmonate‐responsive transcription factor SmMYB1 promotes phenolic acid biosynthesis in *Salvia miltiorrhiza* . Hortic. Res. 8, 1–10. 33384411 10.1038/s41438-020-00443-5 PMC7775463

[pbi70048-bib-0079] Zhou, Z. , Feng, J. , Huo, J. , Qiu, S. , Zhang, P. , Wang, Y. , Li, Q. *et al*. (2024) Versatile CYP98A enzymes catalyse meta‐hydroxylation reveals diversity of salvianolic acids biosynthesis. Plant Biotechnol. J. 22, 1536–1548. 38226779 10.1111/pbi.14284 PMC11123398

