# Supplementary material for: Ecotype‐specific phenolic acid accumulation and root softness in Salvia miltiorrhiza are driven by environmental and genetic factors
Source: Plant Biotechnol J. 2025 Mar 19;23(6):2224–41. doi: 10.1111/pbi.70048 (PMC12120906; doi:10.1111/pbi.70048)
Supplement: Supplementary file 1 — Appendix S1 Supplementary table S1‐S14. [file PBI-23-2224-s003.docx]

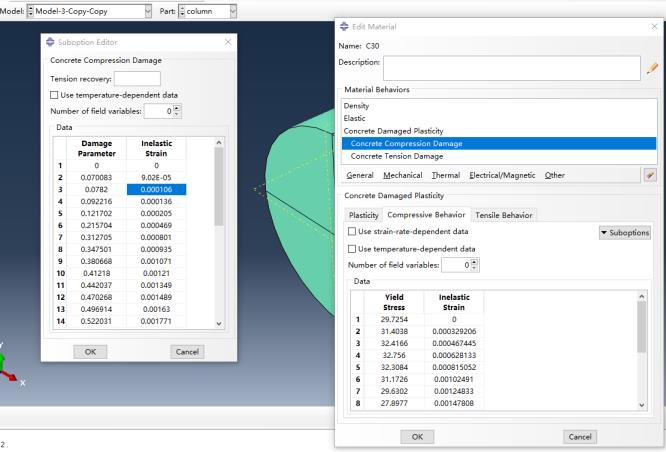
**The stress-strain curve of composite materials exhibits a nonlinear relationship during extrusion, and non convergence results occur when using static and general parameters to simulate the compression process.**

Error：


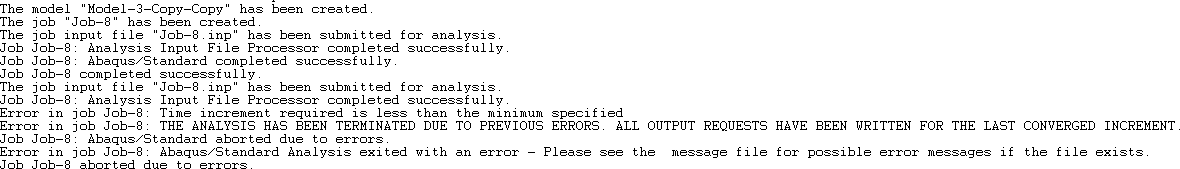


**The bending stress cracking strain curve of composite materials shows a linear relationship, and it is normal to simulate the tensile process using static and general parameters.**


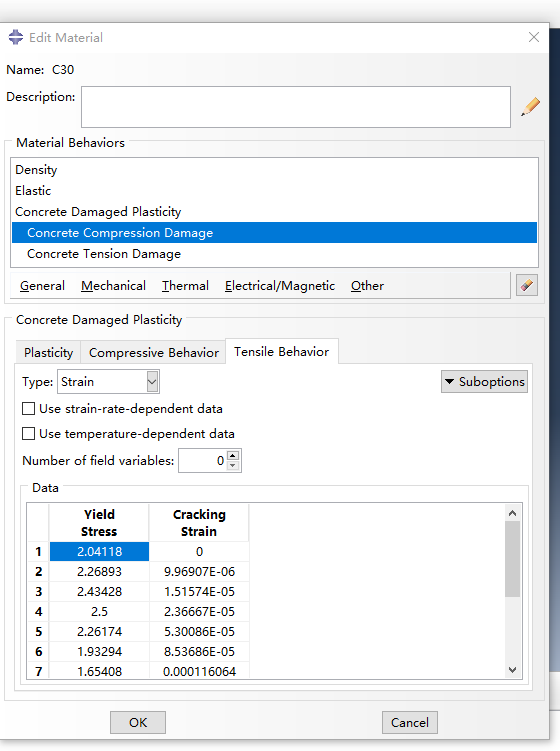

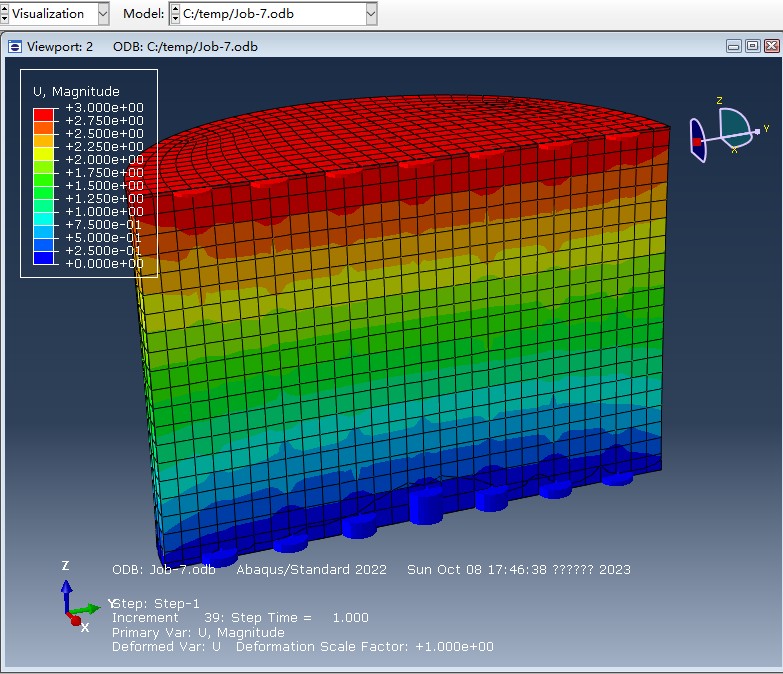

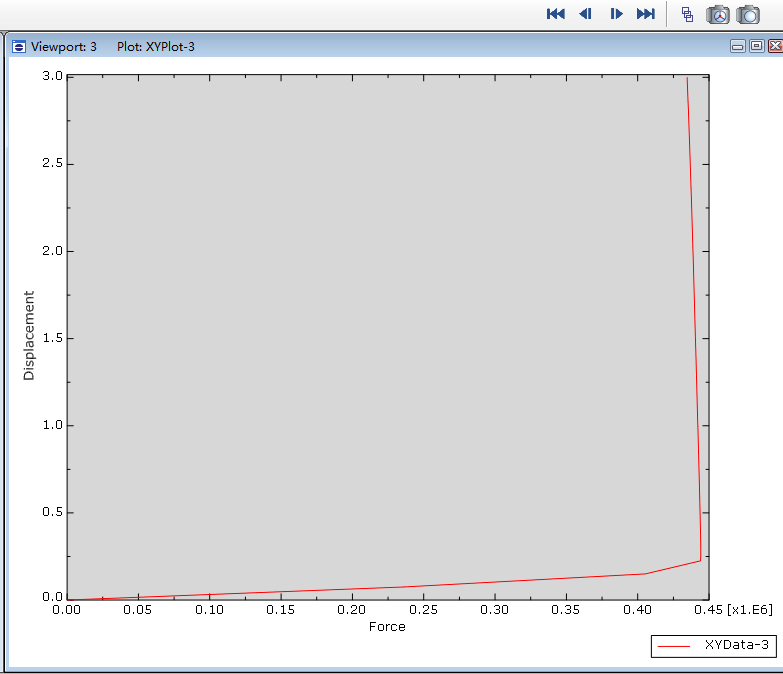


Modeling methods and steps for extrusion deformation process

Stress application setting：Squeeze deformation and bending deformation

1.
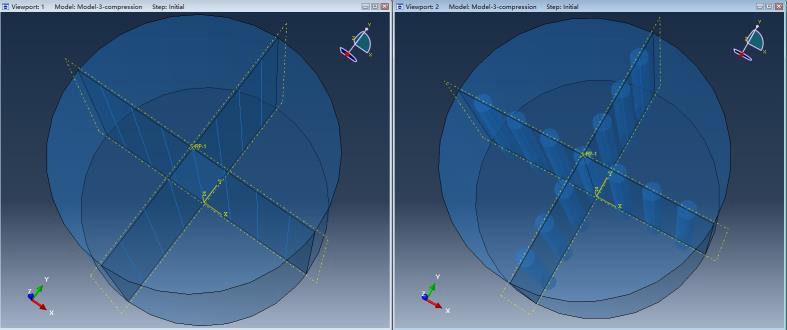
**xylem：Vertical arrangement**


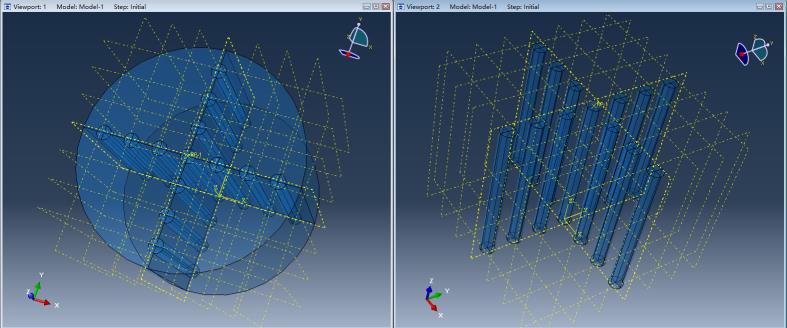


1. **Filler material: flexible (rubber-**


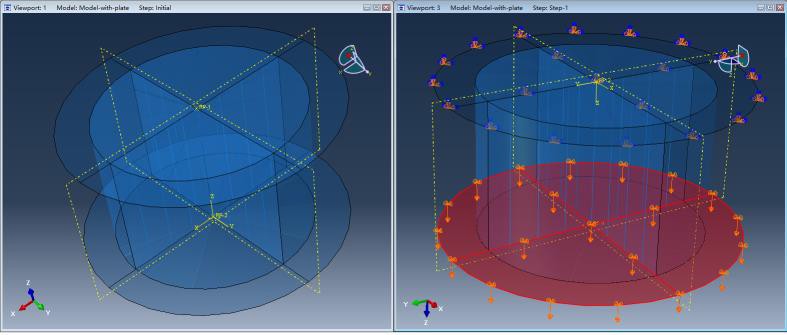


**like)**

1. **Composite material: rigid and high**

**hardness bars inserted into flexible fillers**

1. **Interface bonding method：Embedded region**


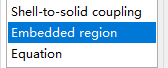


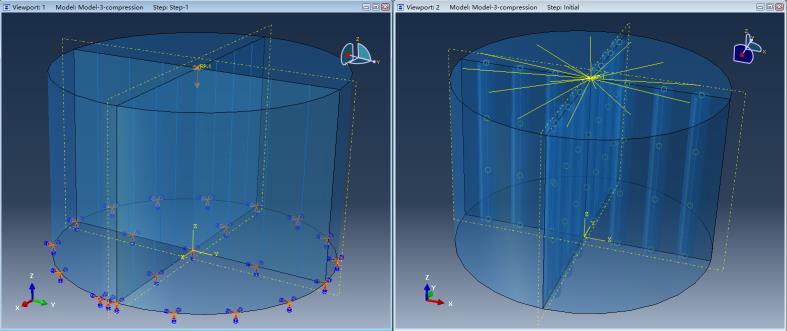


**Stress #1**

**Stress #2**

1. **Tow different directions of stress application**

**Stress application #1**: Fix the bottom plane and apply uniformly distributed downward displacement pressure to the upper plane

**Stress application #2:** Fix the upper and bottom planes and apply bending deformation from the side.


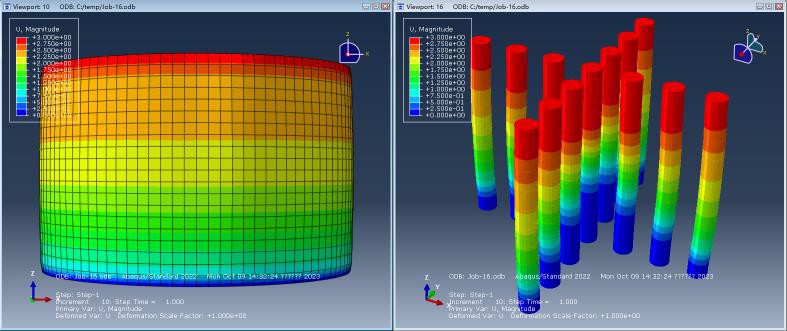

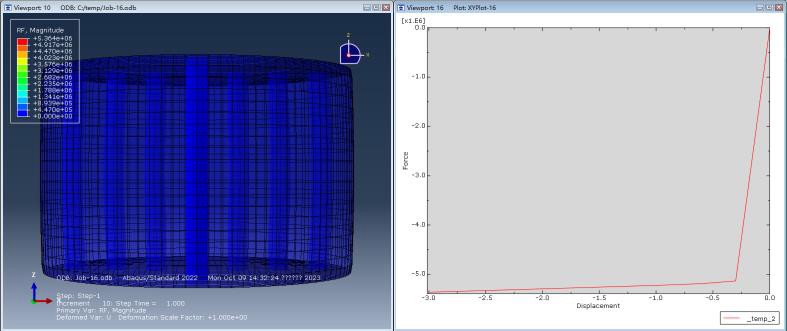
 Compression process: comparison of three modeling methods Displacement cloud map Stress-strain curve

**#1**


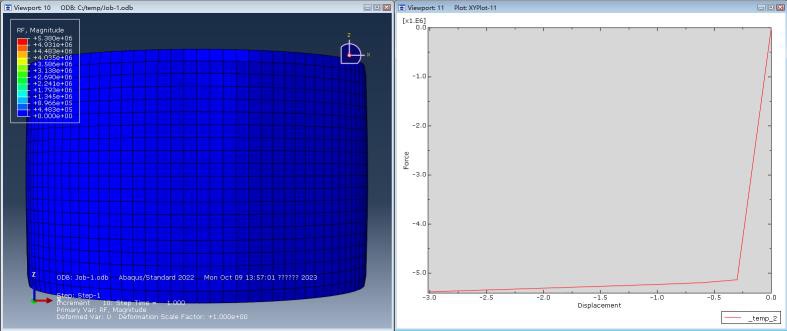
**#2**


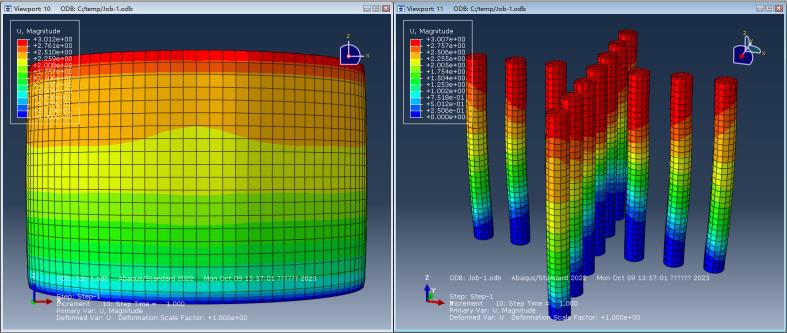


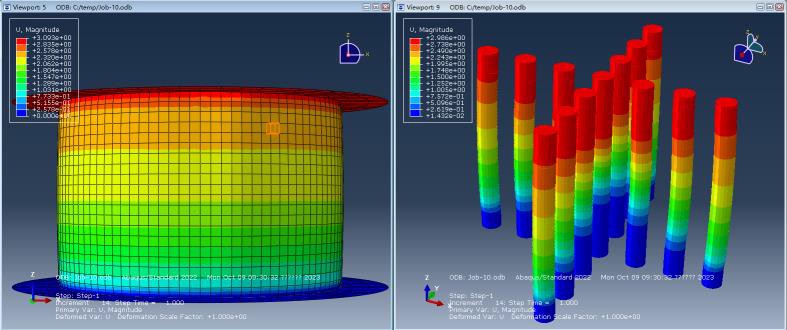
**#3**


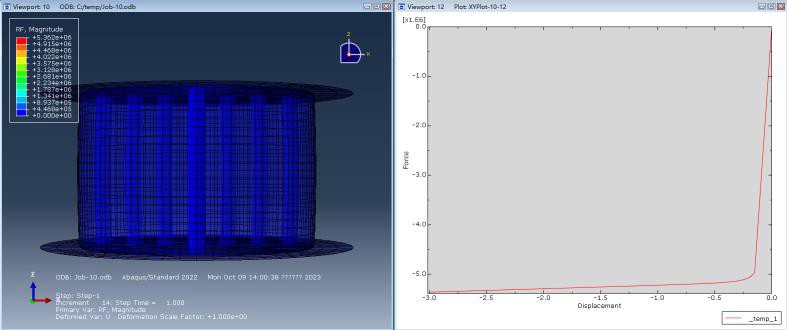


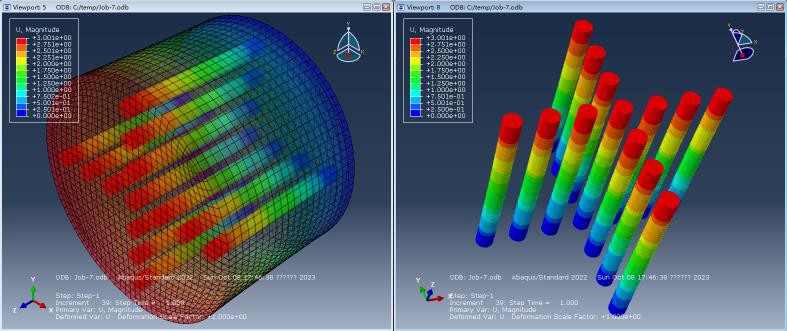

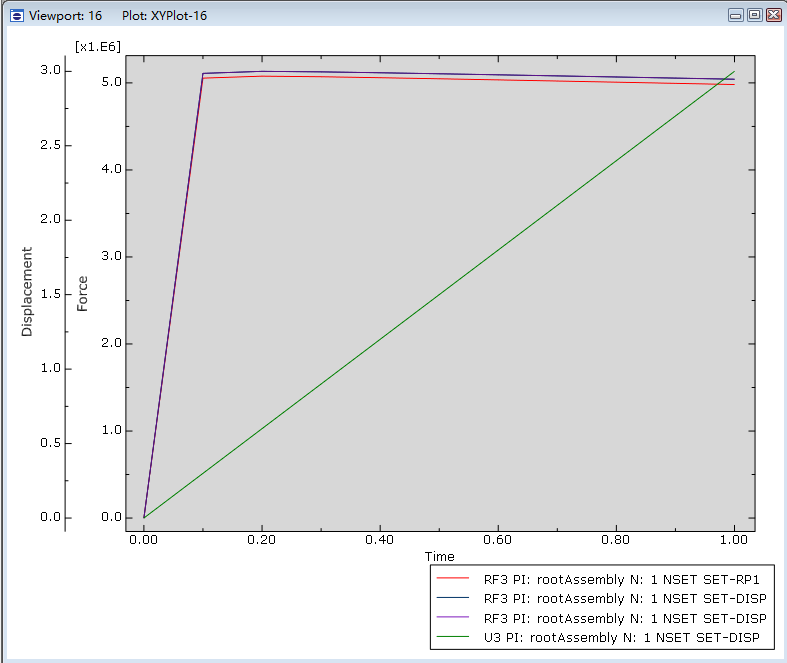
 Stretch/bend process: comparison of three modeling methods Displacement cloud map

**#1**


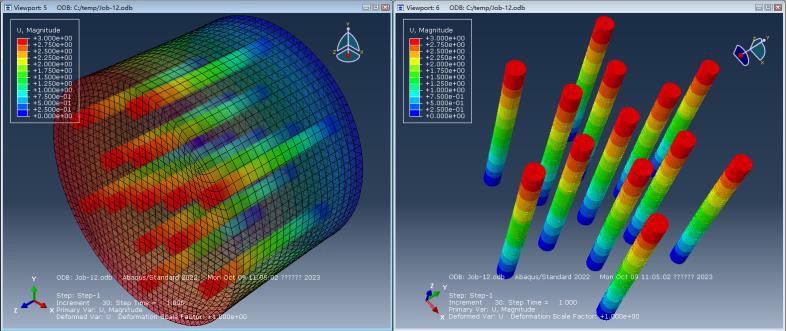
**#2**


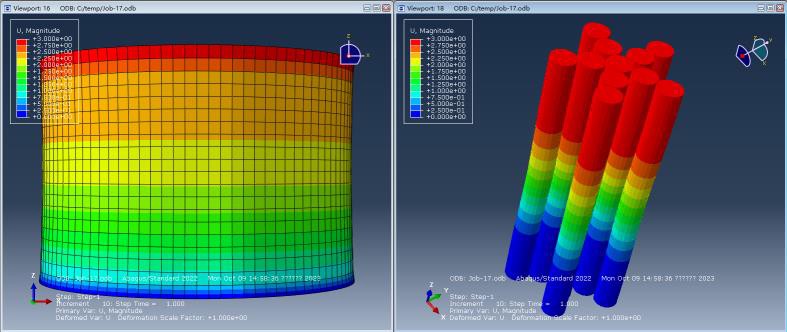
**Purple line:** Stress-time curve **#1**

**#3 Blue line:** Stress-time curve **#2**

**Red line:** Stress-time curve **#3**

**Green Line:** Summary of displacement-time curve

# 3D modeling of bending deformation


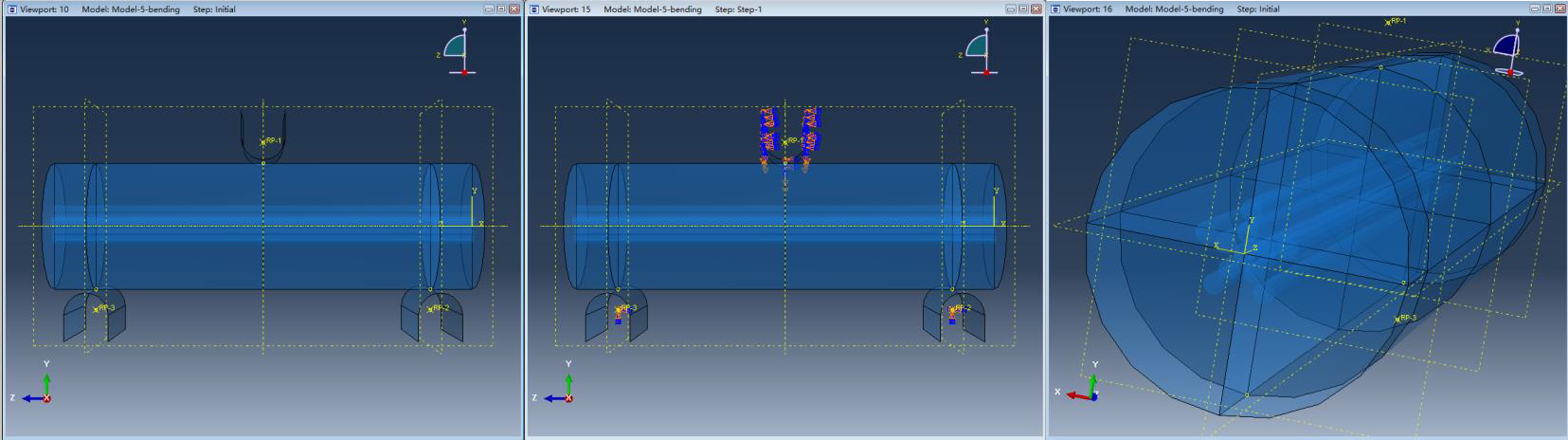


**Stress application method:** Fix the bottom pressure head and apply a uniformly distributed downward displacement to the upper pressure head


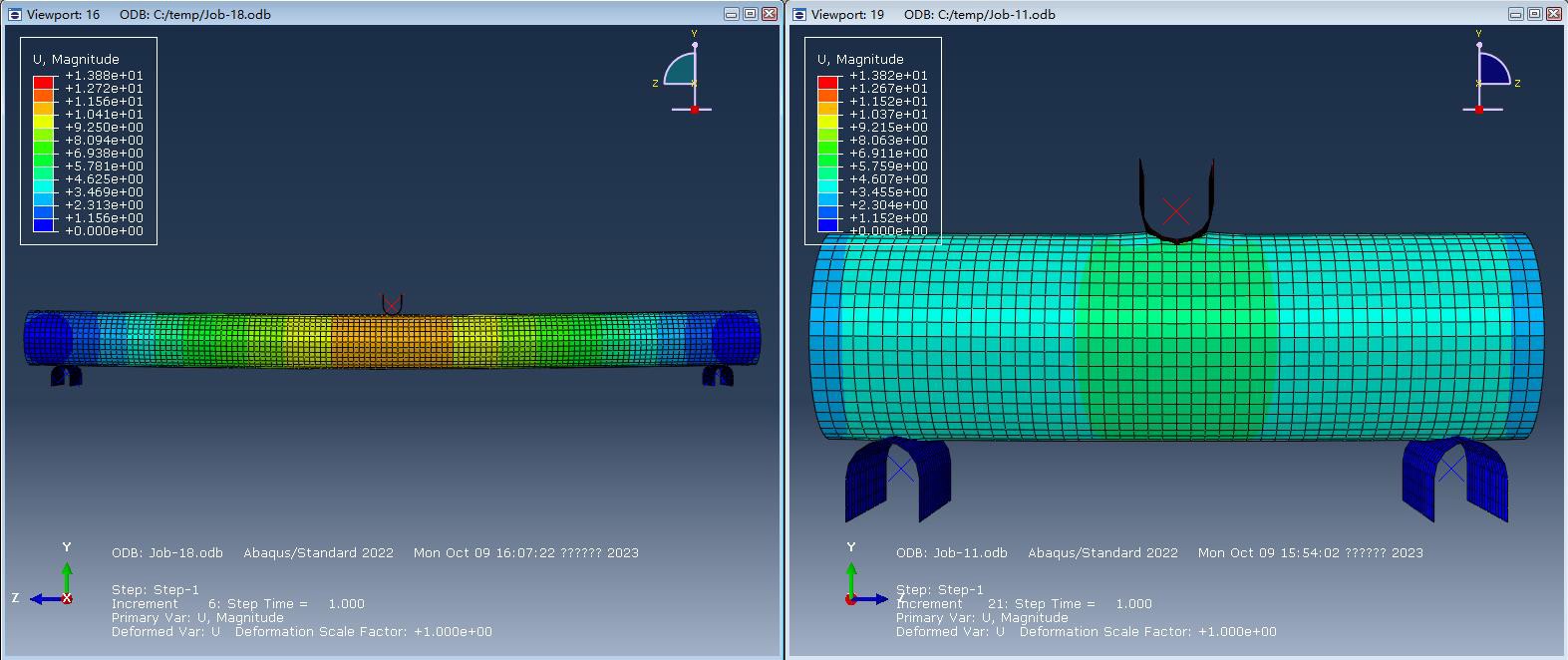


**Define deformation state:** When the displacement reaches 2mm, bending deformation occurs,

and when the displacement reaches 50mm, the indenter is embedded in the composite material.

# Model optimization and stress loading


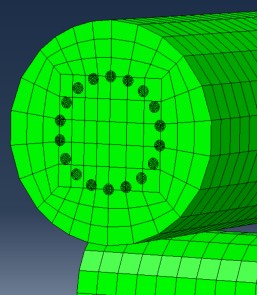

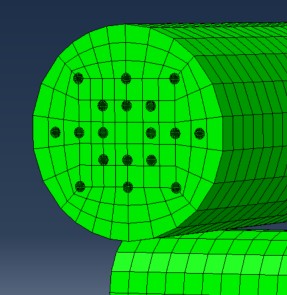


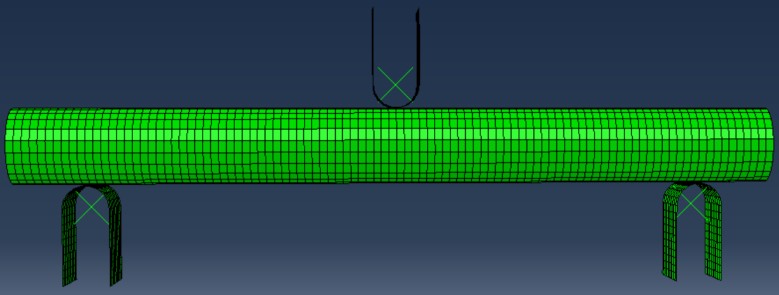


**Model:** Total length 100 mm, outer diameter of substrate 10 mm, inner reinforcement diameter 0.5 mm

**Material:** All are wood, exhibiting anisotropy; The yield stress of the matrix material CLT is 30 MPa, and the yield stress of the inner reinforcement material CLT-1 is 3 MPa

**Boundary condition:** Three point bending simulation, fixed at both ends, applying a 5 mm displacement boundary condition on the top pressure head of the model

**Model 1:** Arranged in a circular pattern, 18 inner bars are evenly arranged on the circumference, with a radius of 5 mm

**Model 2:** Linear arrangement with a spacing of

1.25 mm between adjacent inner bars

**Comparison of 5 loading directions Material parameter settings**


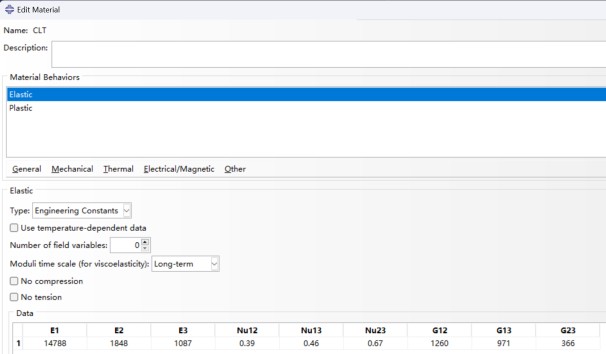

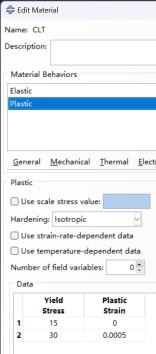

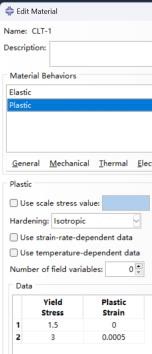

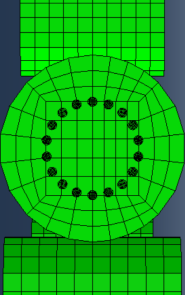

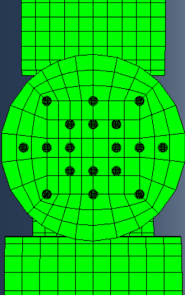

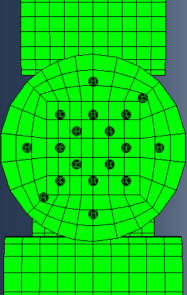

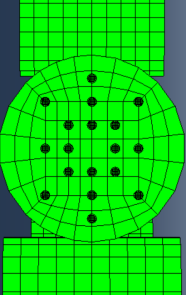

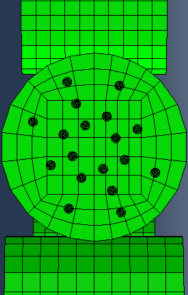


**Model-1**

**Model-2 Model-2-R1 Model-2-R2**

**Model-2-R3**

# Virtual simulation of plastic strain M1-R1

# Dynamic display #


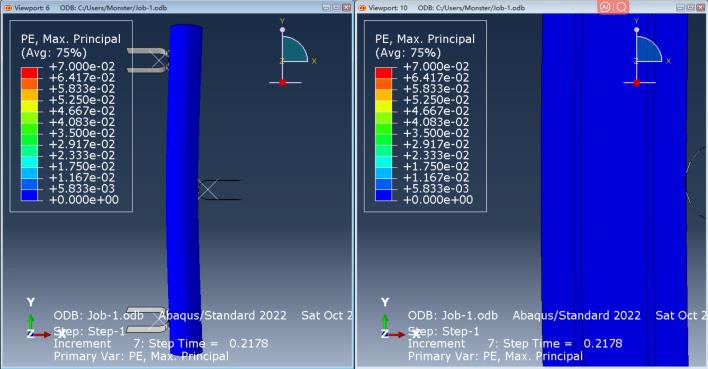

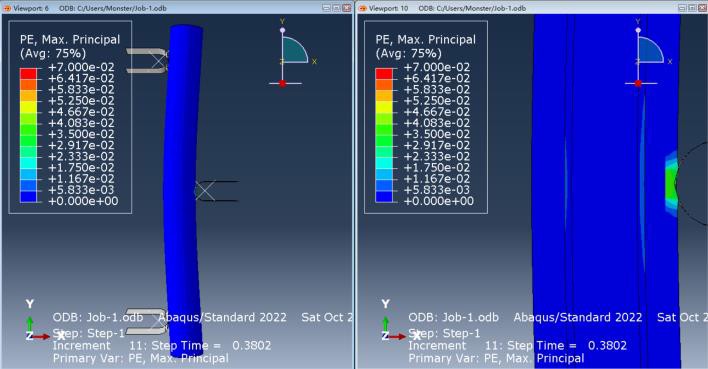


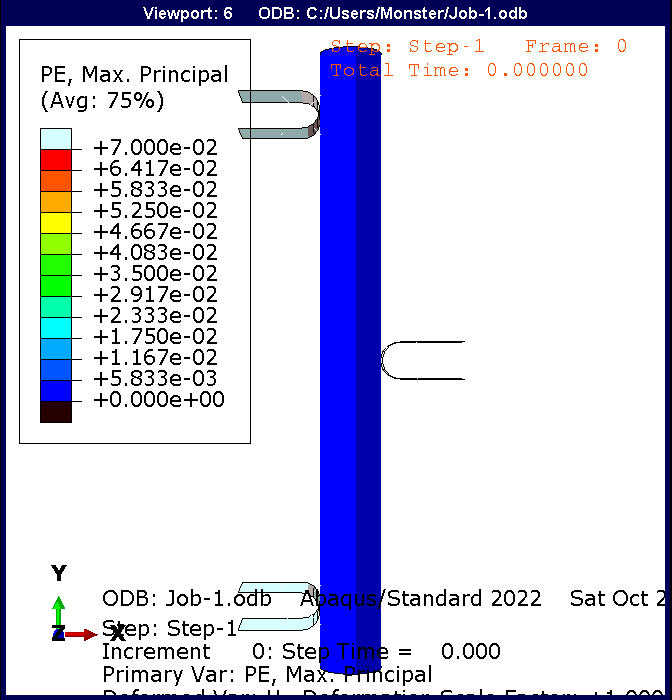
Displacement: 1.1 mm Displacement: 2.0 mm


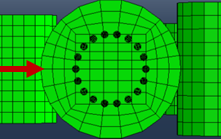

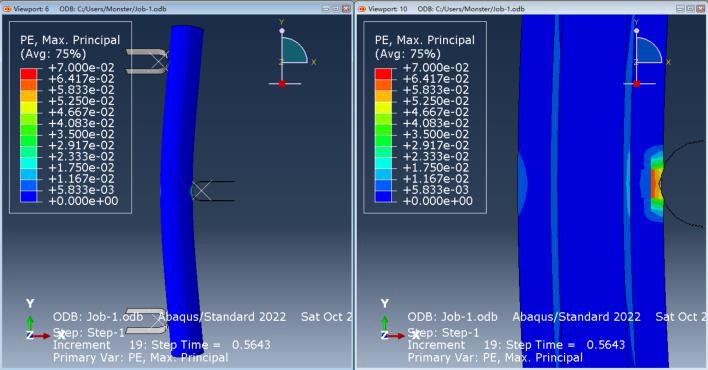

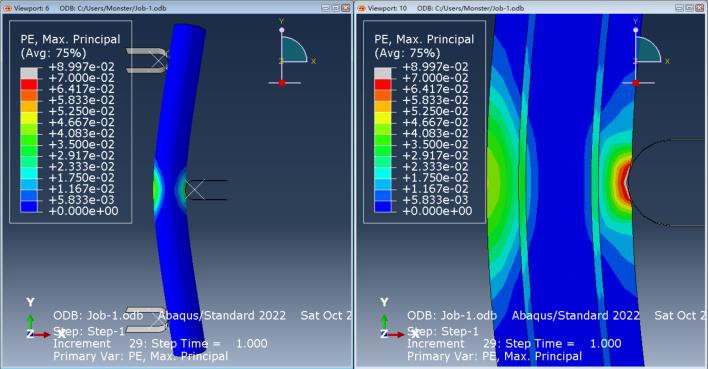


M1-R1 Displacement: 2.9 mm Displacement: 5.0 mm

# Virtual simulation of plastic strain M2-R1

# Dynamic display #


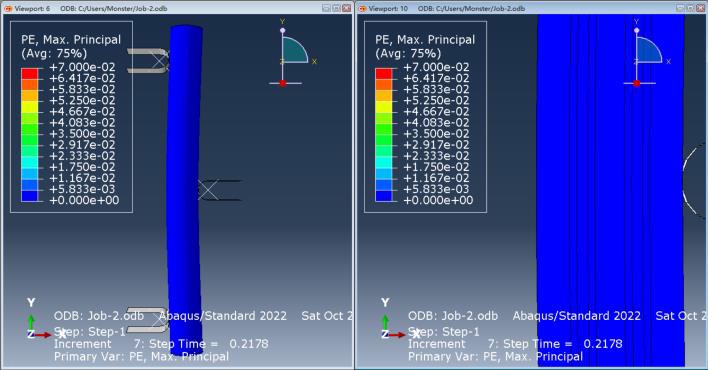

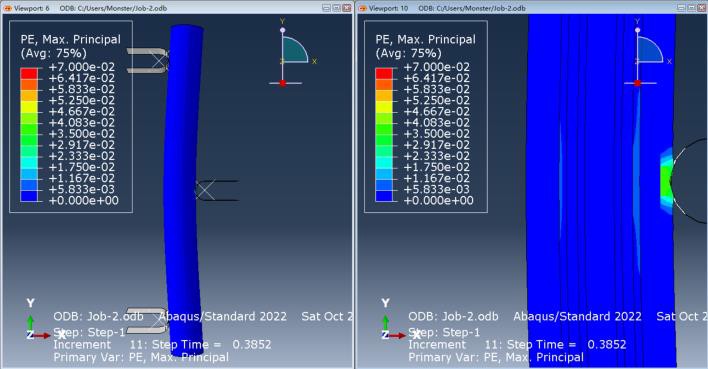


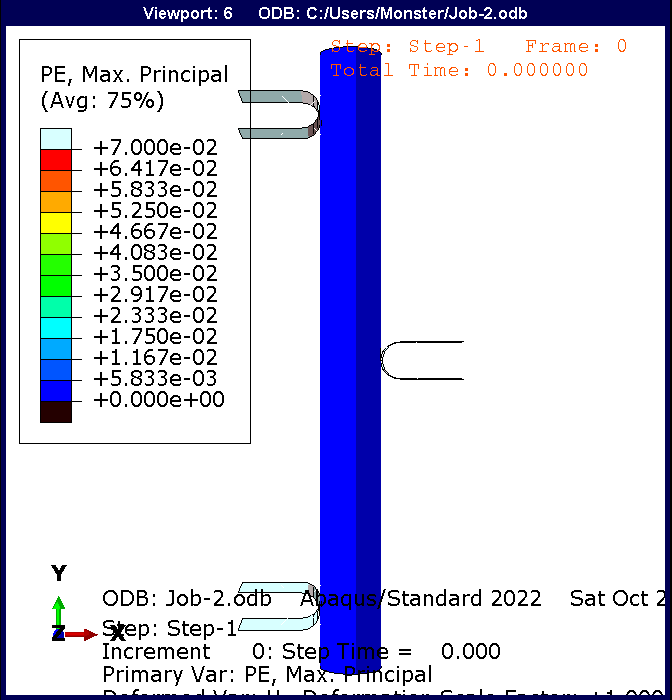
Displacement: 1.1 mm Displacement: 2.0 mm


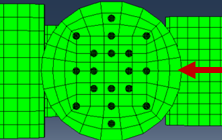


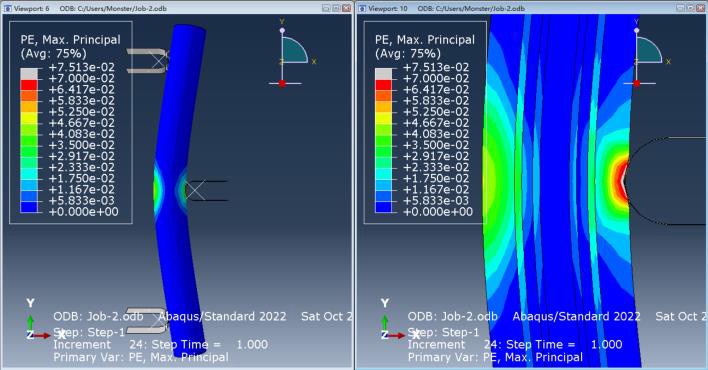

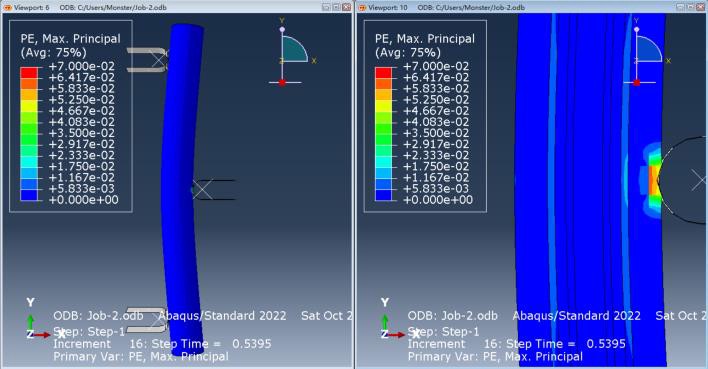


M2-R1 Displacement: 2.8 mm Displacement: 5.0 mm

# Virtual simulation of plastic strain M2-R2

# Dynamic display #


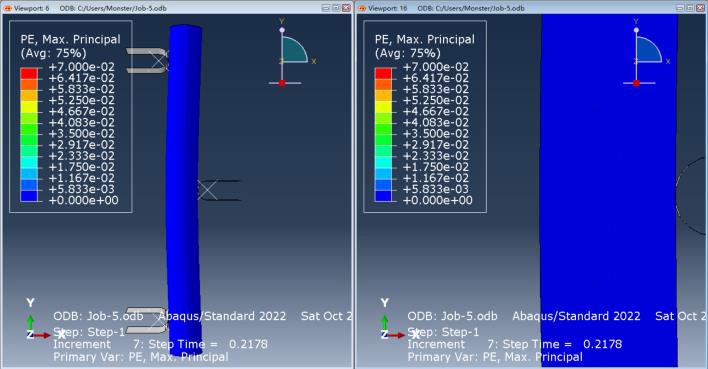

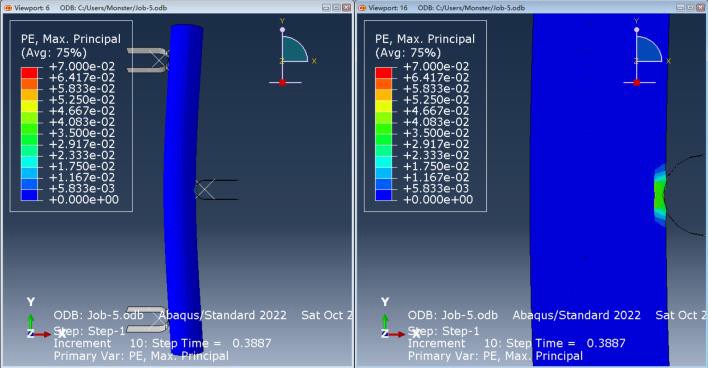


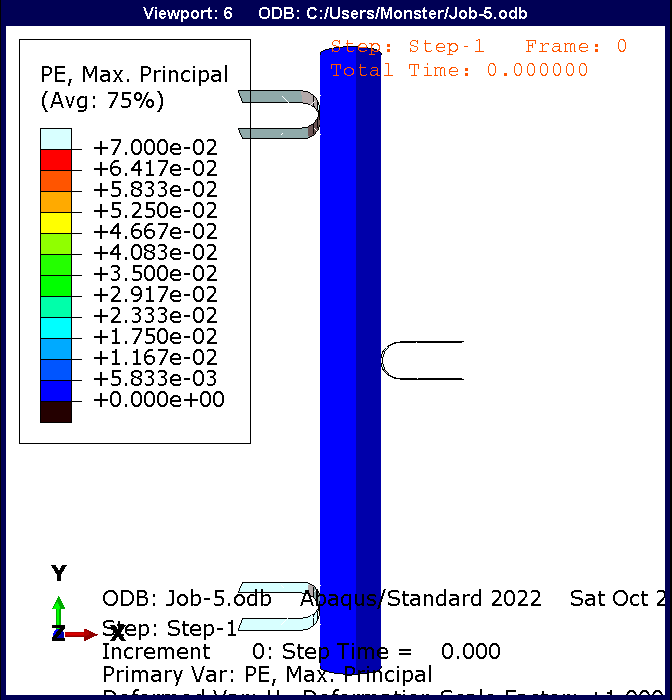
Displacement: 1.1 mm Displacement: 2.0 mm


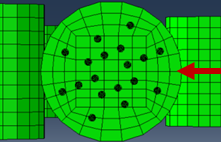


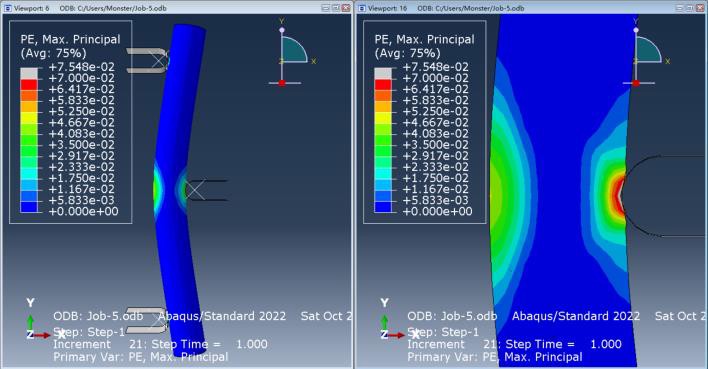

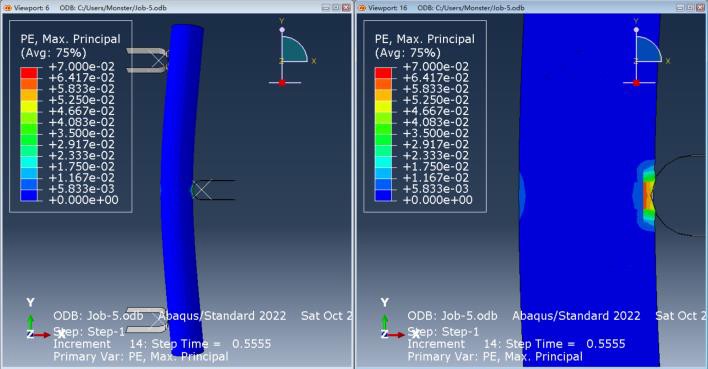


M2-R2 Displacement: 2.9 mm Displacement: 5.0 mm

# Virtual simulation of plastic strain M2-R3

# Dynamic display #


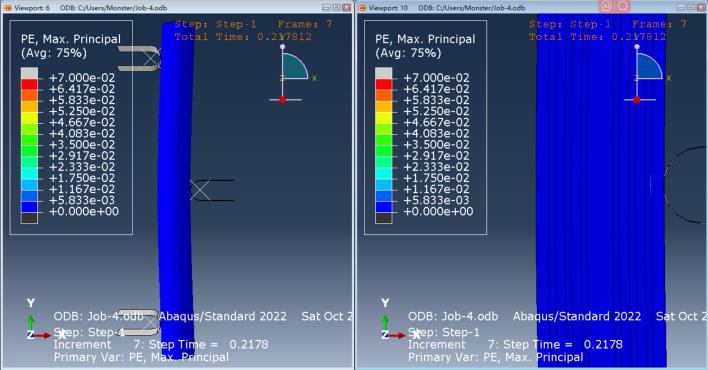

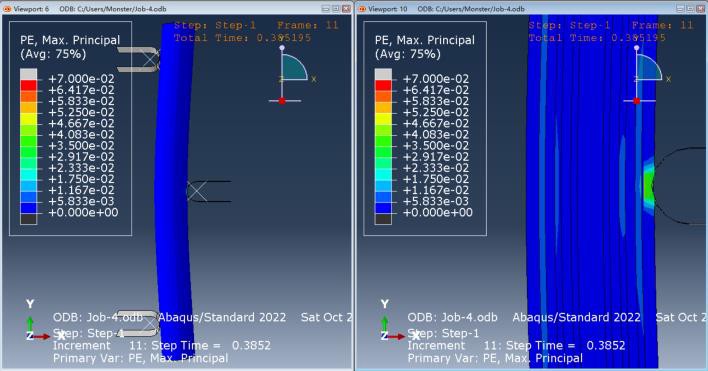


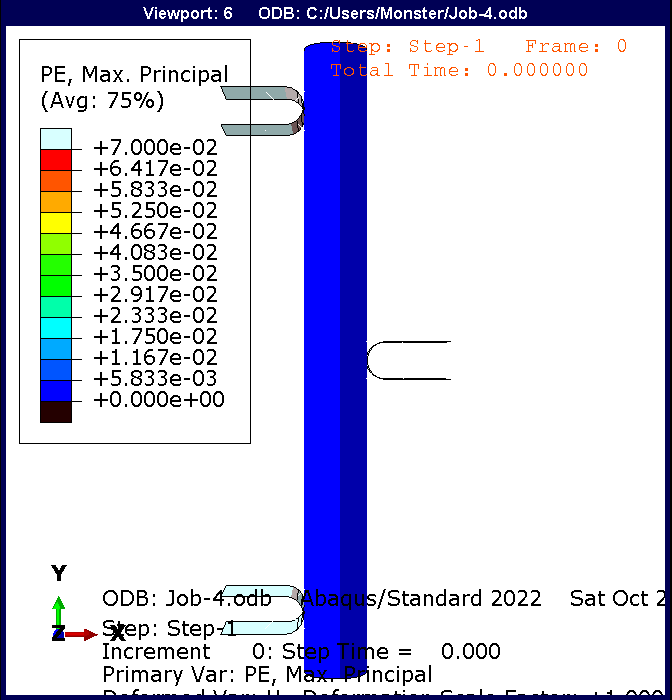
Displacement: 1.1 mm Displacement: 2.0 mm


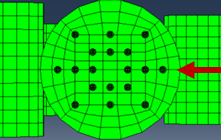

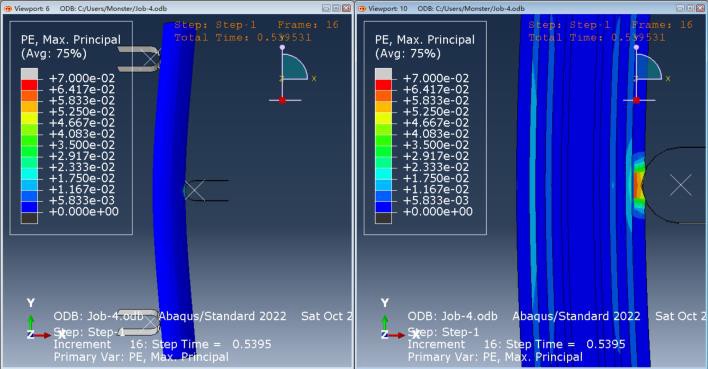

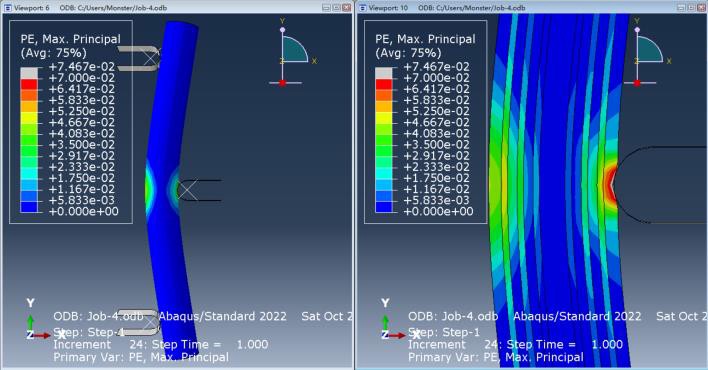


M2-R3 Displacement: 2.8 mm Displacement: 5.0 mm

**Analysis of stress-strain curve during bending deformation process**


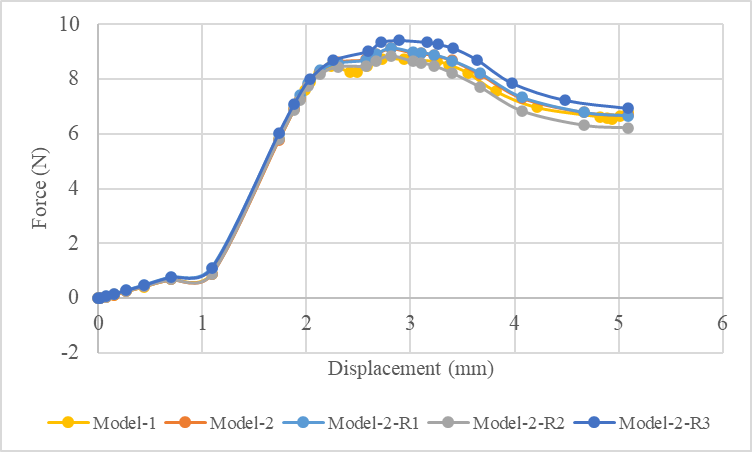


**Analysis of stress-strain curve during extrusion deformation process**


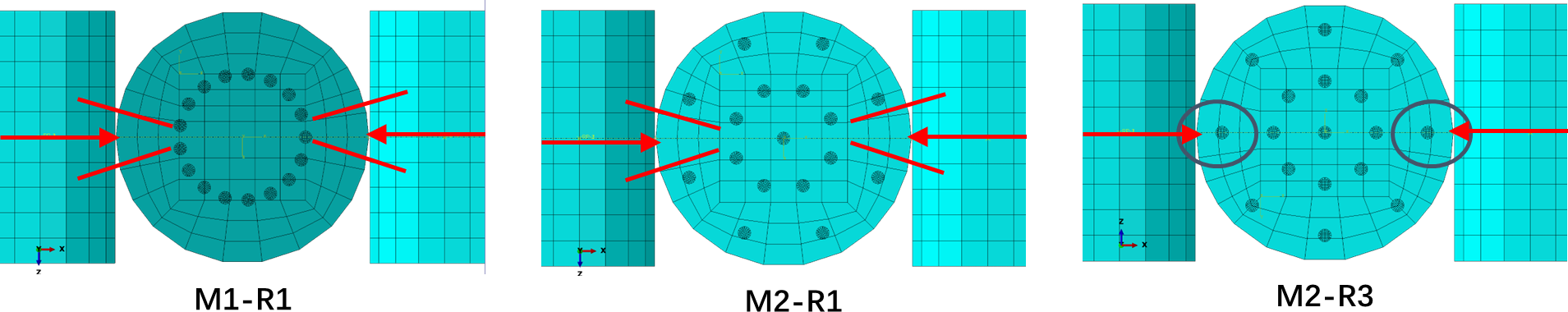


# Dynamic display #


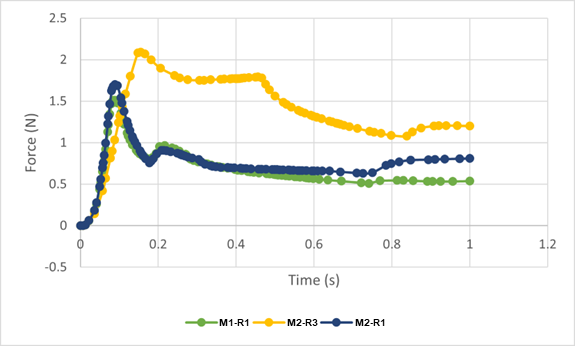


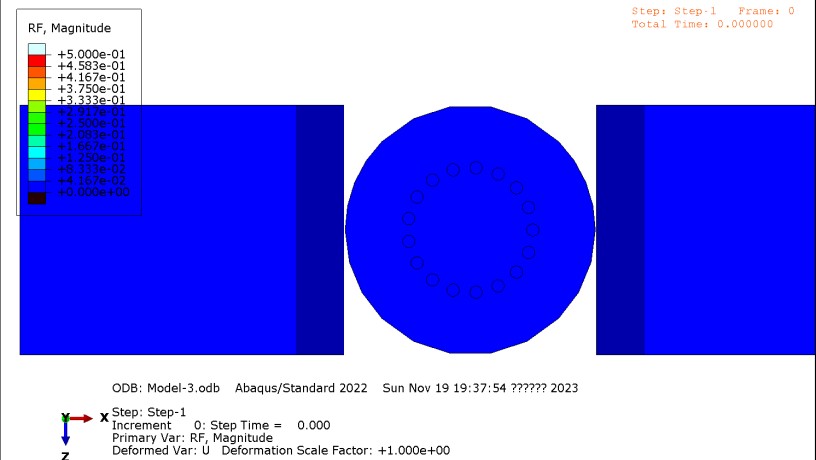


**M1-R1**


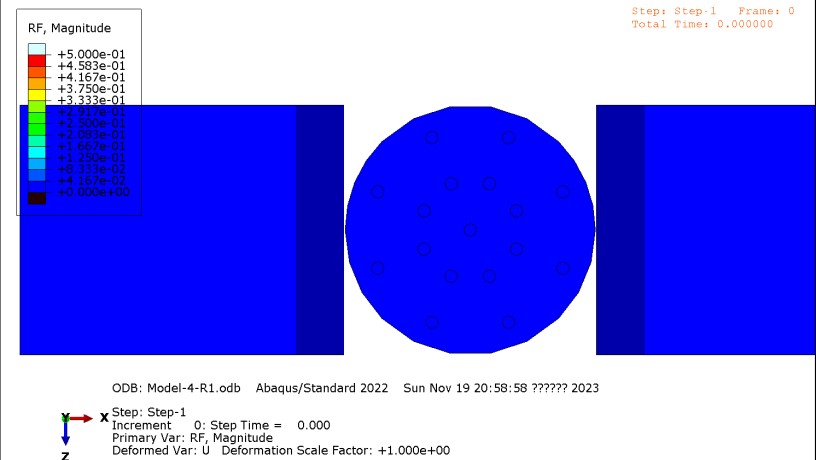


**M2-R1**


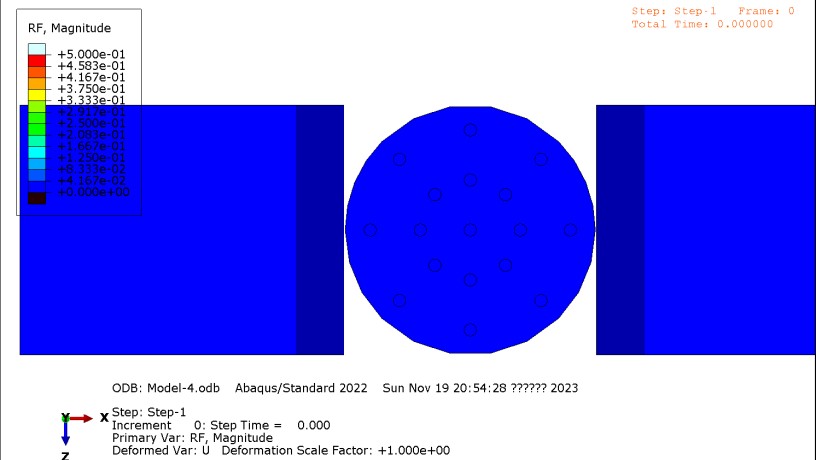


**M2-R3**
