# Supplementary material for: Ecotype‐specific phenolic acid accumulation and root softness in Salvia miltiorrhiza are driven by environmental and genetic factors
Source: Plant Biotechnol J. 2025 Mar 19;23(6):2224–41. doi: 10.1111/pbi.70048 (PMC12120906; doi:10.1111/pbi.70048)
Supplement: Supplementary file 2 — Figure S1 Meteorological map of environmental meteorological factors in different S. miltiorrhiza producing areas. Figure S2 Phenotype and composition detection of S. miltiorrhiza root under UV‐B exposure, cold stress and Cu2+ stress. Figure S3 3D modeling and stress simulation of the effect of different xylem arrangement on mechanical strength of roots. Figure S4 Comparative metabolome analysis of Sm.SC and Sm.SD mature roots. Figure S5 Statistics of whole genome sequencing and chromosome mount information. Figure S6 Comparative transcriptomic analysis of Sm.SC and Sm.SD mature roots. Figure S7 Transcription factor‐metabolite association analysis of phenolic acid metabolic pathways. Figure S8 Cross‐species evolutionary analysis of the WRKY40‐CCR‐HCT metabolic cluster. Figure S9 Subcellular localization and expression pattern of SmWRKY40. Figure S10 Preparation flow of transgenic/gene‐edited hairy roots. Figure S11 qPCR analysis of phenolic acid metabolism pathway genes in hairy roots and hair root. Figure S12 Identification of Arabidopsis mutants and transgenic plants. Figure S13 SmWRKY40 binding peaks on promoters of other pathway genes. Figure S14 Comparison of the binding of WRKY40 transcription factors to the promoters of the RAS and HCT2 genes. Figure S15 Haplotype comparison of rosmarinic acid synthase (RAS) from Sm.SC and Sm.SD. Figure S16 Association analysis of RA and SAB content with haplotypes and acyltransferase activity in population samples, and groping of reaction conditions of rosmarinic acid synthase (RAS) from Sm.SC and Sm.SD. Figure S17 Phylogeny and multiple sequence alignment of SmRAS and homologous sequences. Figure S18 Identification of Arabidopsis mutants and transgenic plants. Figure S19 Heterologous expression validates the biological functions of SmWRKY40 and SmRASs in response to cold stress. Figure S20 Heterologous expression validates the biological functions of SmWRKY40 and SmRASs in response to Cu2+ stress. [file PBI-23-2224-s002.docx]

**Supplemental information**


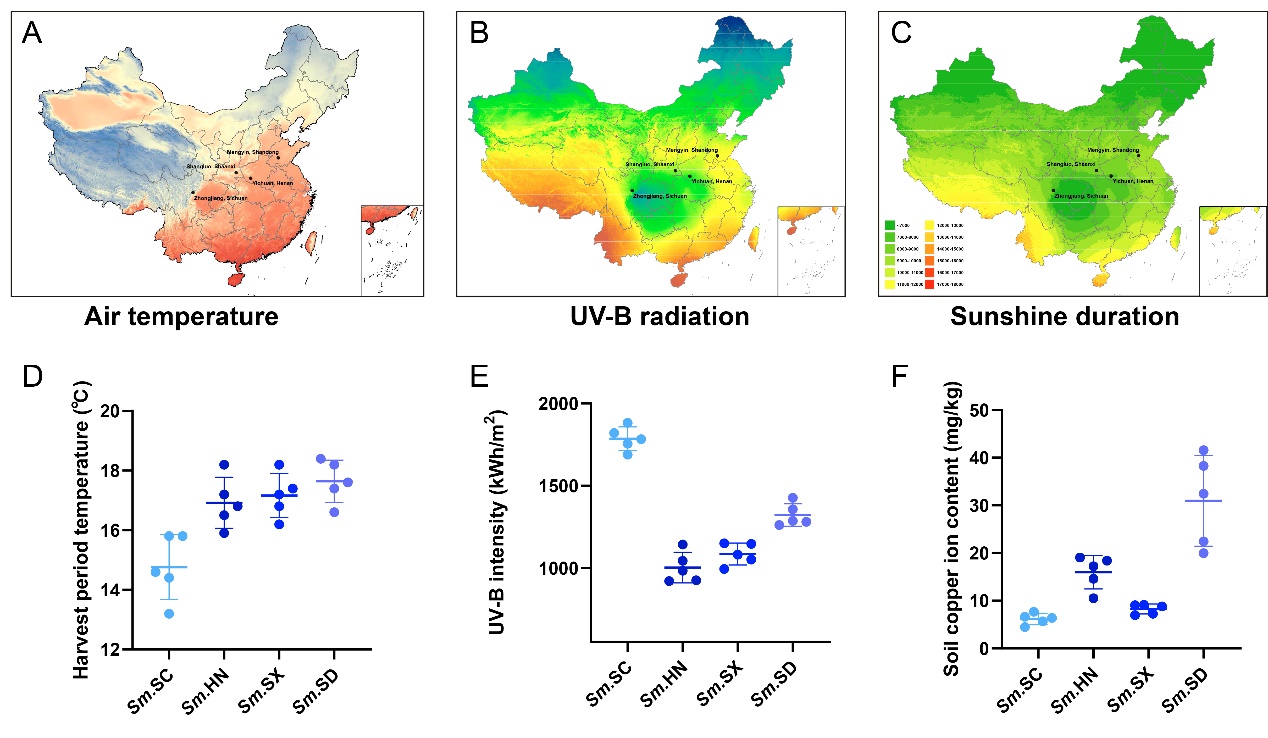


**Fig S1.** Meteorological map of environmental meteorological factors in different *S. miltiorrhiza* producing areas. **A.** Average daily temperature at root expansion stage. **B.** Annual UV-B radiation dose. **C.** Annual sunshine radiation intensity. Data were collected from the National Comprehensive Meteorological Information Sharing Platform (CIMISS, 2023). **D-F.** Total of 5 sampling sites were randomly selected in the 4 production areas to monitor the average daily temperature (D), UV-B radiation intensity (E), and soil Cu^2+^ content (F) during the harvest period.


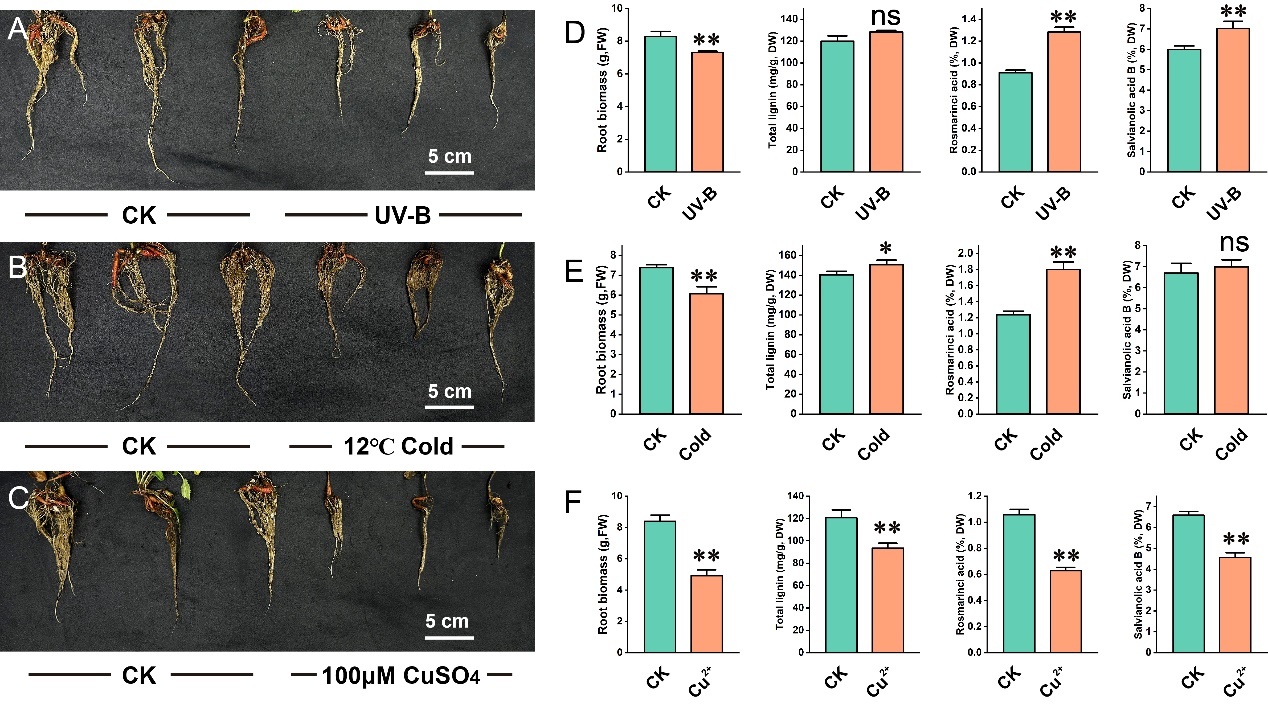


**Fig S2.** Phenotype and composition detection of *S. miltiorrhiza* root under UV-B exposure, cold stress and Cu^2+^ stress. **A-C.** Phenotypes of *S. miltiorrhiza* root under UV-B exposure, cold stress and Cu^2+^ stress. **D-F.** Rosmarinic acid, salvianolic acid B and total lignin content of *S. miltiorrhiza* root under UV-B exposure, cold stress and Cu^2+^ stress. Data are means ± *SD*, n=3.


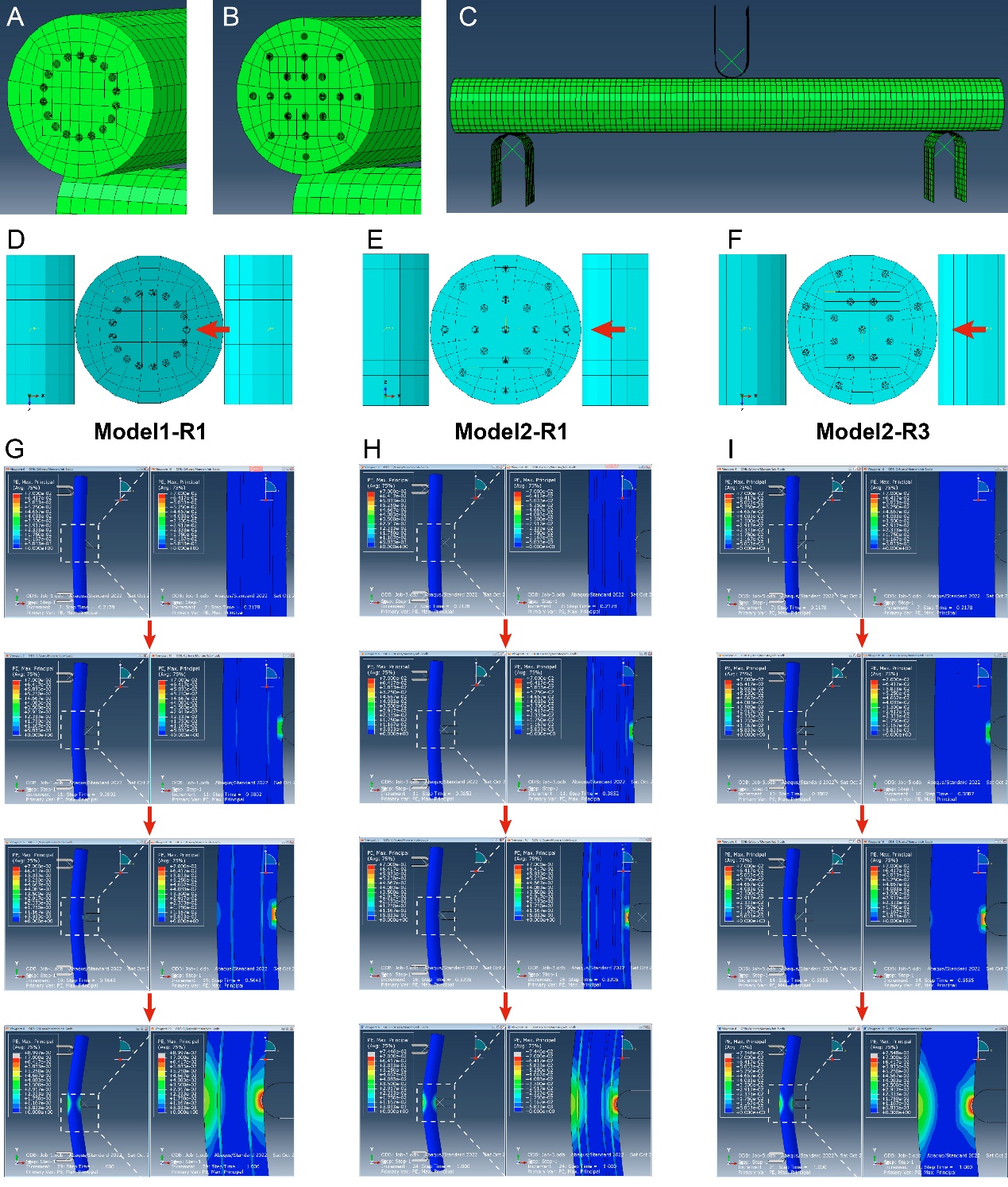


**Fig S3.** 3D modeling and stress simulation of the effect of different xylem arrangement on mechanical strength of roots. **A-C.** Schematic of 3D modeling and deformation simulation. Controlling the same amount of xylem (high-strength, stringy structures located in the center) only changes the way the xylem is arranged inside the fleshy components (low-hardness materials). **D**, **G.** The dynamics of the ring-arranged xylem during extrusion deformation is simulated. **E**, **H.** The kinetic process of the radially aligned xylem during opposing extrusion deformation is simulated. **F**, **I.** Simed the dynamics of the radially aligned xylem during adjacent extrusion deformation.


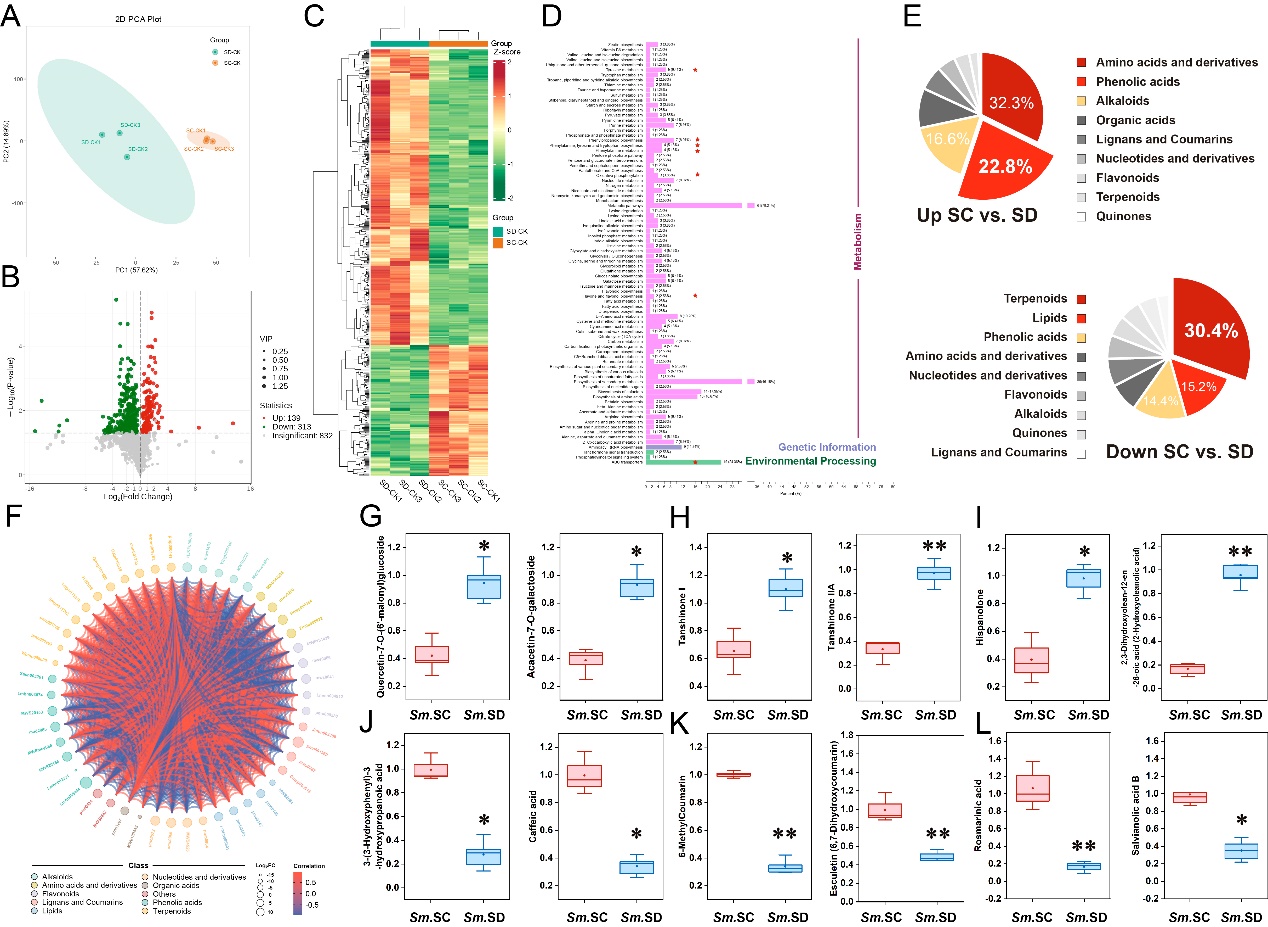


**Fig S4.** Comparative metabolome analysis of *Sm.*SC and *Sm.*SD mature roots. **A.** Principal component analysis (PCA). **B.** Volcano plots of the differential metabolite. **C.**

Heat map of the differential metabolites. **D.** Enrichment analysis of keg metabolic pathway for differential metabolites. **E.** Pie chart of the content distribution of the differential metabolites. **F.** Network plot of the correlation analysis of the differential metabolites. **G-I.** Differences in the content of representative flavonoids, tanshinone and phenolic acids in *Sm.*SC and *Sm.*SD. All data show the arithmetic mean ± *SD* from 3 biological replicates. Different letters indicate significant differences at *P* < 0.05 (one-way ANOVA, Tukey’s posttest).


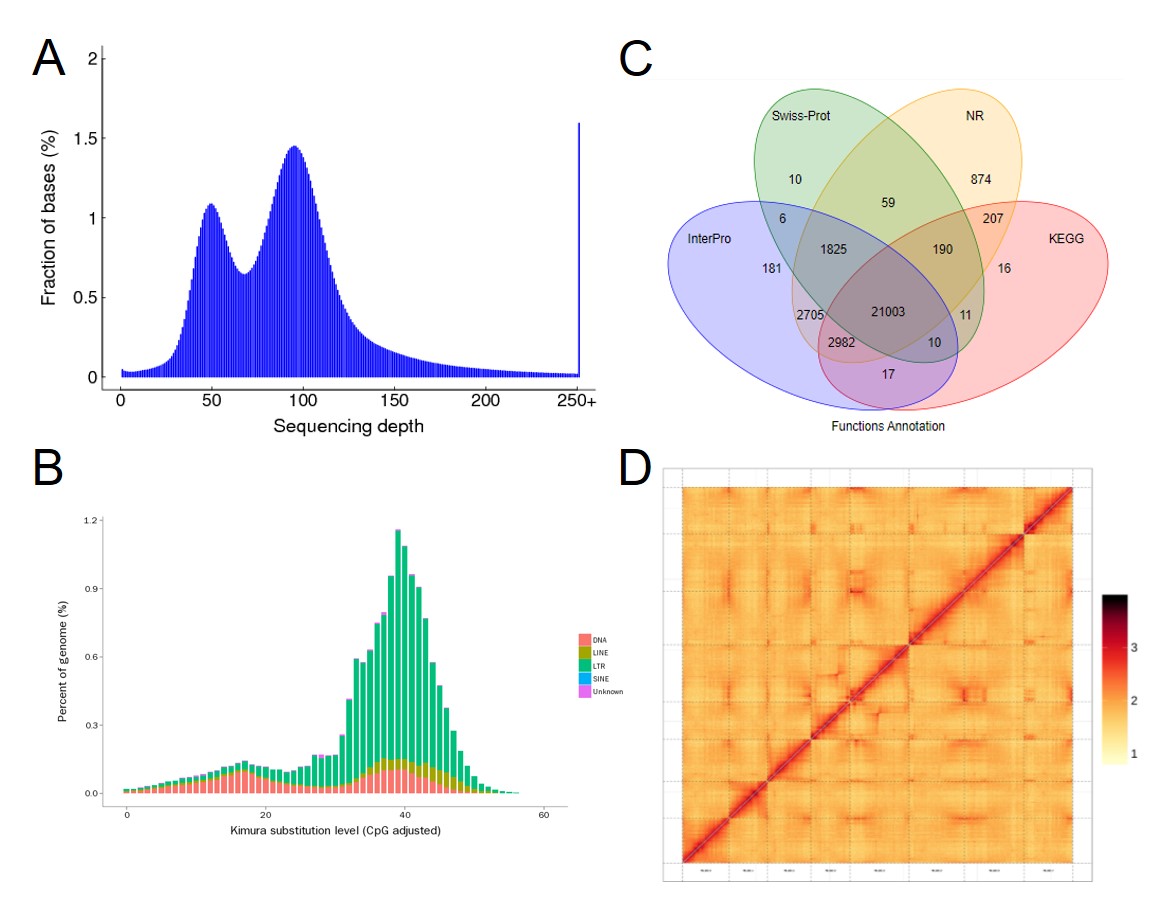


**Fig S5.** Statistics of whole genome sequencing and chromosome mount information. **A.** Sequencing depth statistics. **B.** Venn diagram of gene structure annotation. **C.** Gene element distribution statistics. **D.** Interaction heat map of Hi-C assisted assembly.


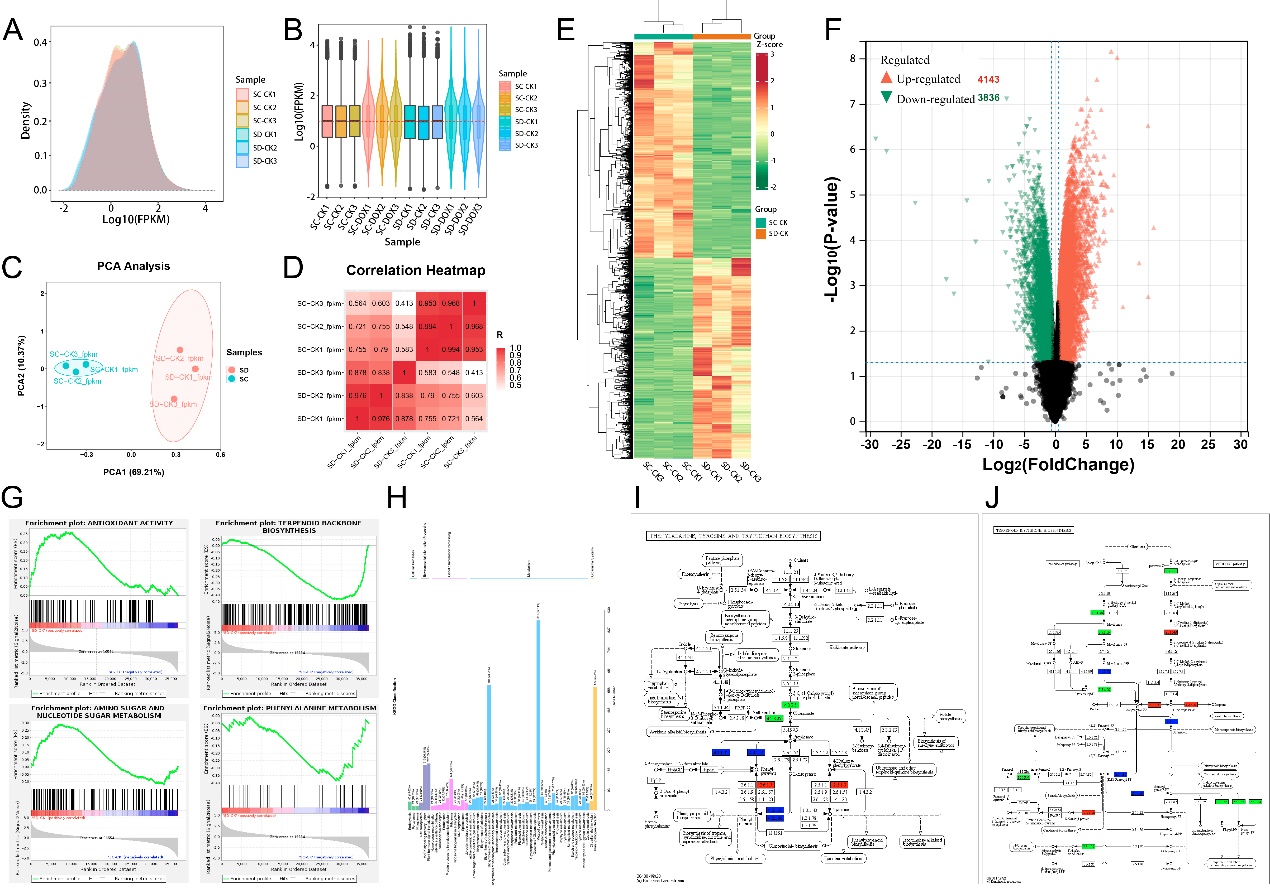


**Fig S6.** Comparative transcriptomic analysis of *Sm.*SC and *Sm.*SD mature roots. **A.** Transcript expression abundance statistics. **B.** Normalization analysis of expression levels. **C.** Principal component analysis (PCA) between samples. **D.** Correlation analysis between samples. **E.** Cluster heatmap of differentially expressed genes. **F.** Dynamic volcano map of differentially expressed genes. **G.** GSEA enrichment analysis of differential metabolic pathways. **H.** Enrichment analysis of KEGG pathway for differentially expressed genes. **I-J.** Mapping differentially expressed genes to specific steps in phenolic acid and terpenoid metabolic pathways.


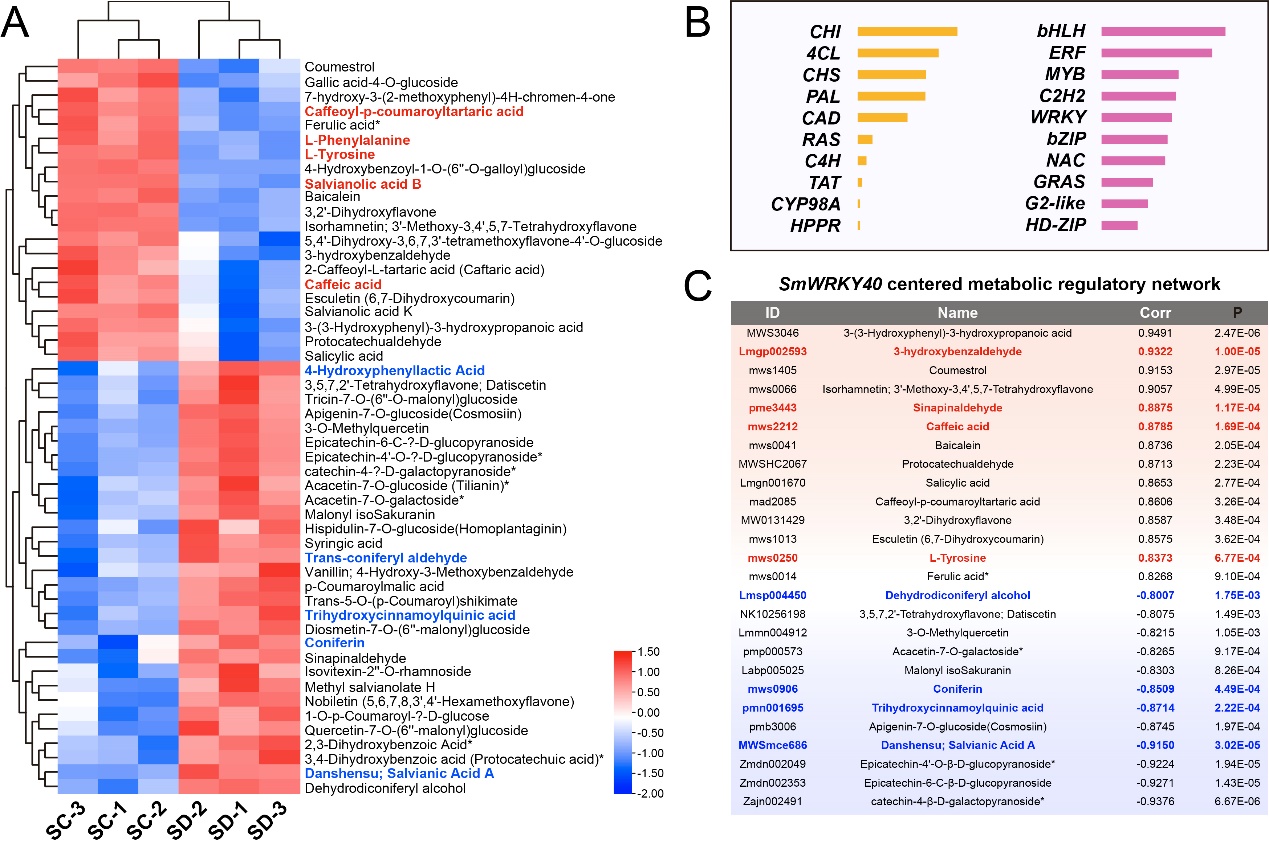


**Fig S7.** Transcription factor-metabolite association analysis of phenolic acid metabolic pathways. **A.** Heat map of differential metabolites (DEM) in the phenylalanine and tyrosine pathways. **B.** Statistics of transcription factors and key enzymes of metabolic pathways in the TFs-DEM regulatory network. **C.** Metabolic pathway compounds forming a regulatory network with *SmWRKY40*.


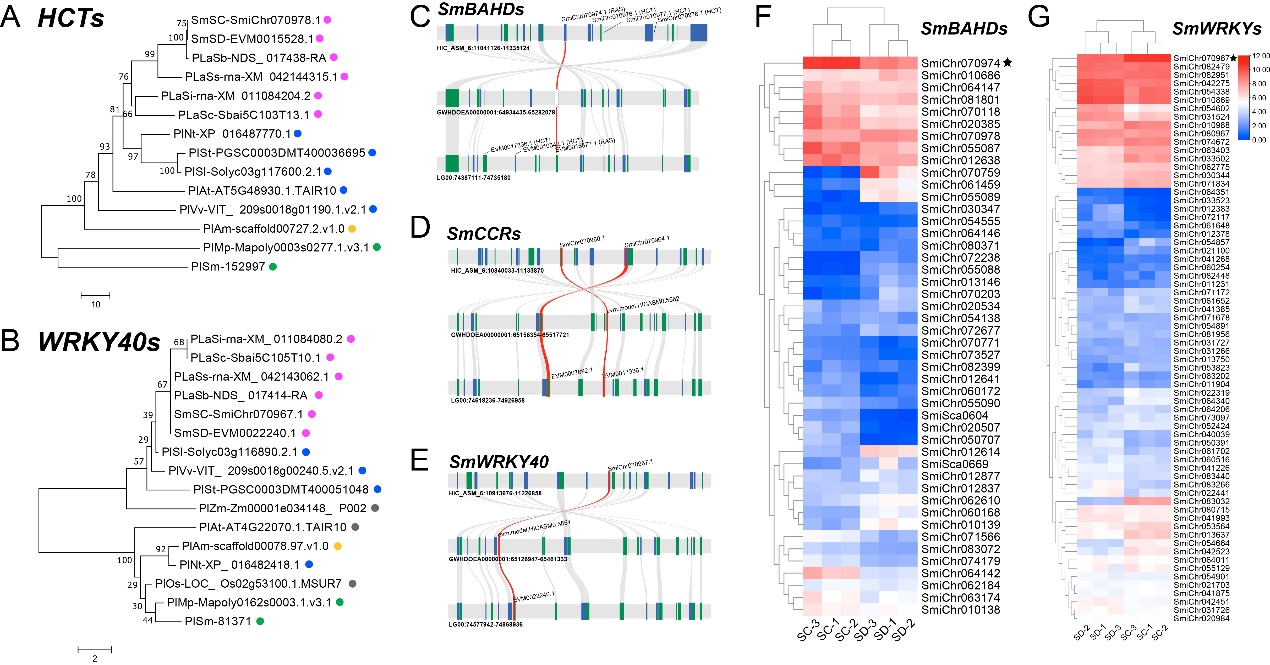


**Fig S8.** Cross-species evolutionary analysis of the *WRKY40*-*CCR*-*HCT* metabolic cluster. **A.** Phylogenetic tree of Shikimate/Quinate Hydroxycinnamoyl-transferase (*HCT*) genes in algae (deletions), moss, monocots, and dicotyledons. **B.** Phylogenetic tree of *WRKY40* genes in algae (deletions), moss, monocots, and dicotyledons. **C-E.** Chromosomal collinearity of *HCT*, *CCR*, and *WRKY40* in *Sm.*SC, *Sm.*SD and *Sm.*SX. **F-G.** Heatmap of the differential expression of *BAHD* genes and *WRKY* TFs in *Sm.*SC and *Sm.*SD.


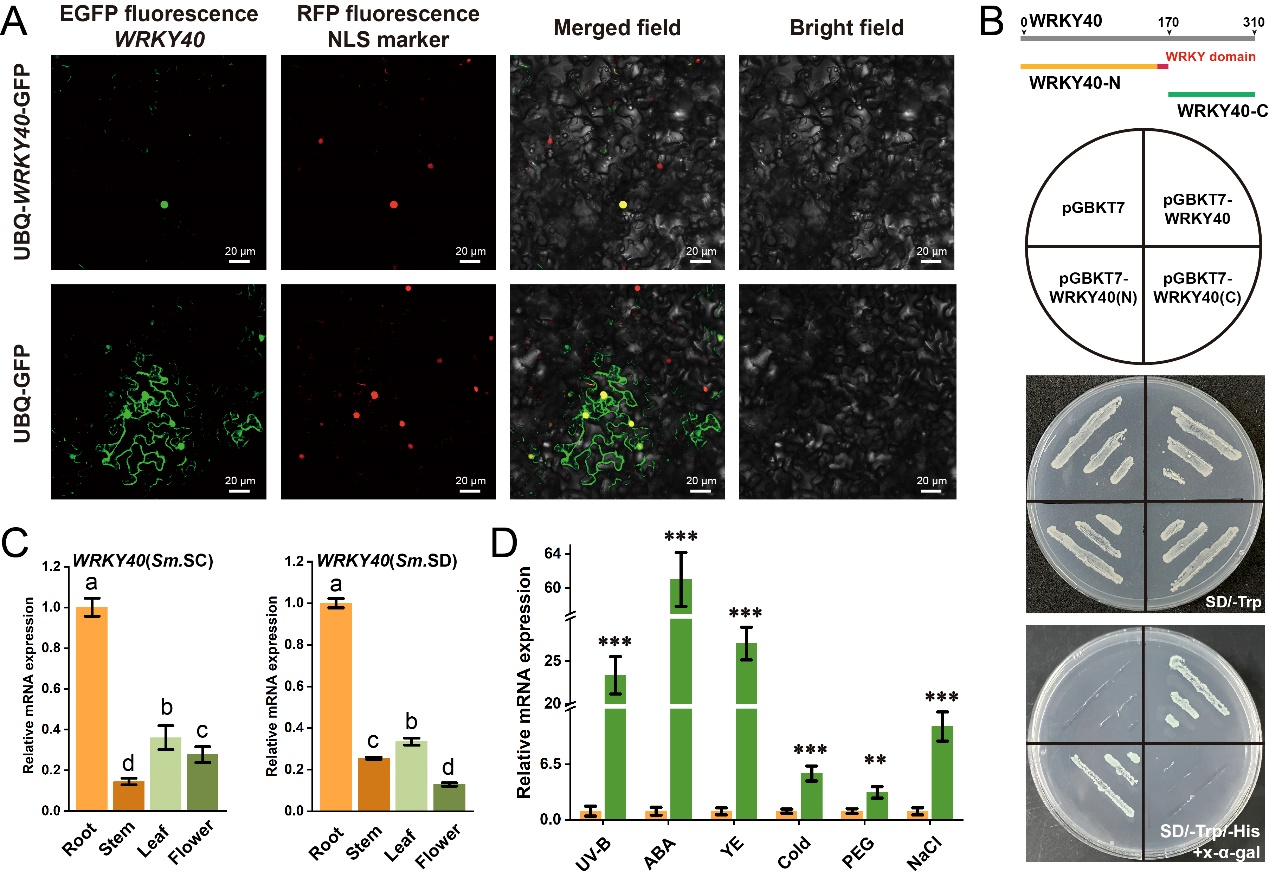


**Fig S9.** Subcellular localization and expression pattern of SmWRKY40. **A.** Subcellular localization of SmWRKY40. Green fluorescence (GFP) represents the SmWRKY40 recombinant protein, and red fluorescence (RFP) represents the nuclear marker. The superimposed images show yellow fluorescence. **B.** Analysis of the transcription activation activity of SmWRKY40. The full-length, N-terminus (1 aa-170 aa) and C-terminus (171 aa-310 aa) of SmWRKY40 were constructed on the pGBKT7 vector, transferred into Y187 yeast and inoculated in SD/-Trp/-His/ + x-α-Gal. Blue colonies demonstrated transcriptional activation activity. **C.** Relative expression of SmWRKY40 in the roots, stems, leaves, and flowers of *Sm.*SC and *Sm.*SD. **D.** Expression levels of *SmWRKY40* under different environmental stresses and elicitors. All data show the arithmetic mean ± *SD* from 3 biological replicates. Different letters indicate significant differences at *P* < 0.05 (one-way ANOVA, Tukey’s posttest).


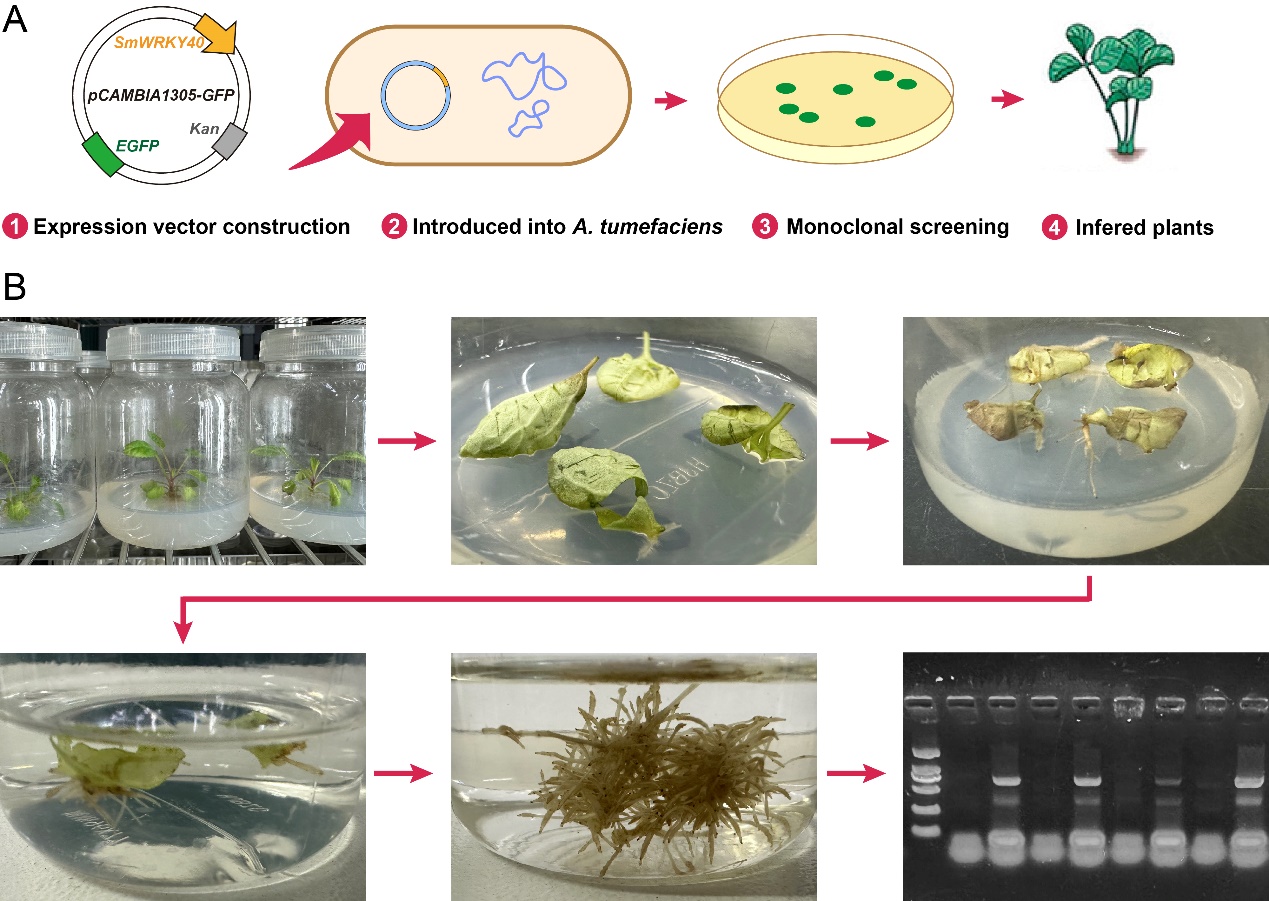


**Fig S10.** Preparation flow of transgenic/gene-edited hairy roots. **A.** Construction of overexpression/gene editing vectors, transformation of *Agrobacterium*, callus infection and refining of transgenic plants. **B.** Bacterial-free seedling preparation, *Agrobacterium* infection, co-culture, solid rooting, liquid suspension culture, and PCR identification.


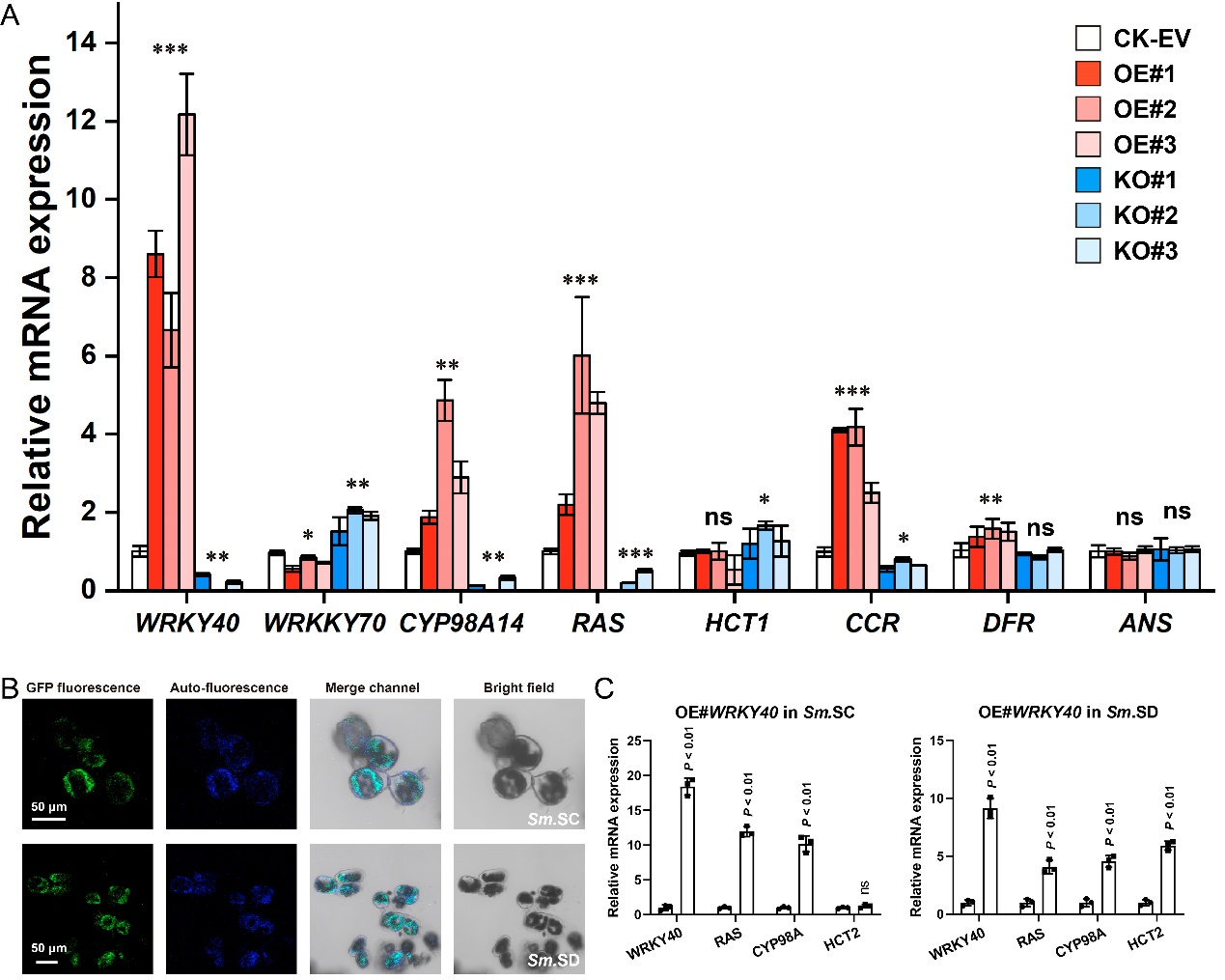


**Fig S11.** qPCR analysis of phenolic acid metabolism pathway genes in hairy roots and hair root. **A.** qPCR analysis of phenolic acid metabolism pathway genes in hairy roots overexpressing and knocking down *WRKY40* gene. **B.** The UBQ-GFP vector was introduced into *S. miltiorrhiza* protoplasts, and the feasibility of protoplast transformation was verified by laser confocal imaging. **C.** The *WRKY40* gene was transiently expressed in the *Sm.*SC and *Sm.*SD protoplasts, followed by qpcr of the phenolic acid metabolism pathway genes among them.


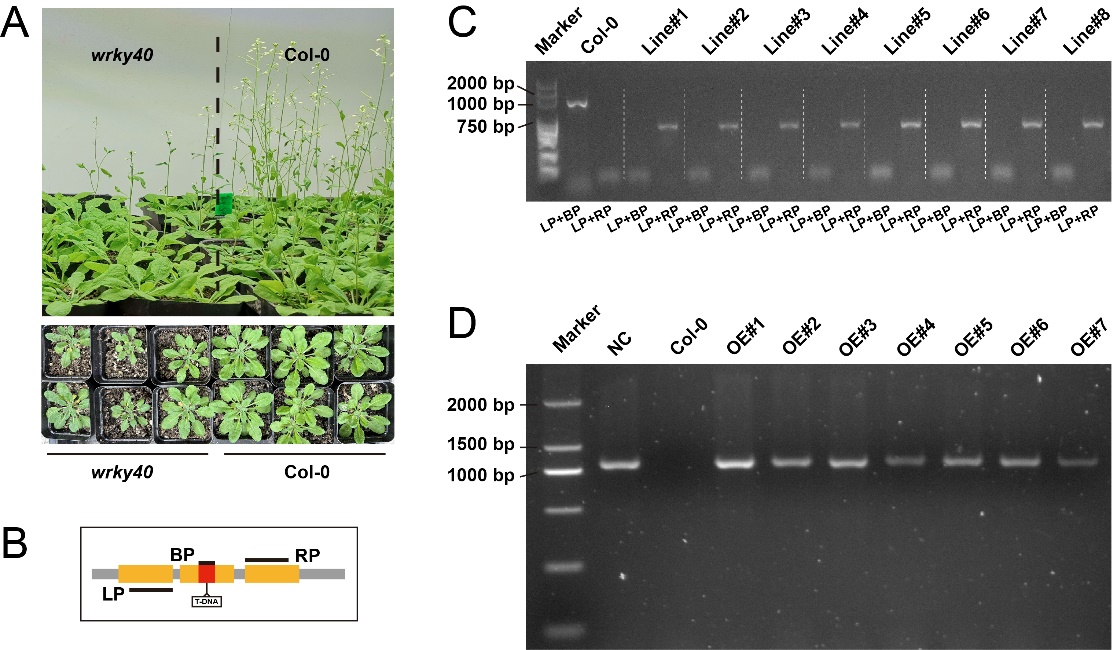


**Fig S12.** Identification of *Arabidopsis* mutants and transgenic plants. **A.** Phenotype comparison of *atwrky40* mutant and col-0 wild type. **B.** A Schematic representation of the structure of the T-DNA insertion mutants. **C.** Triple-primer method PCR identification of T-DNA insertion mutants. **D.** PCR Identification of *SmWRKY40* overexpressing *Arabidopsis*.


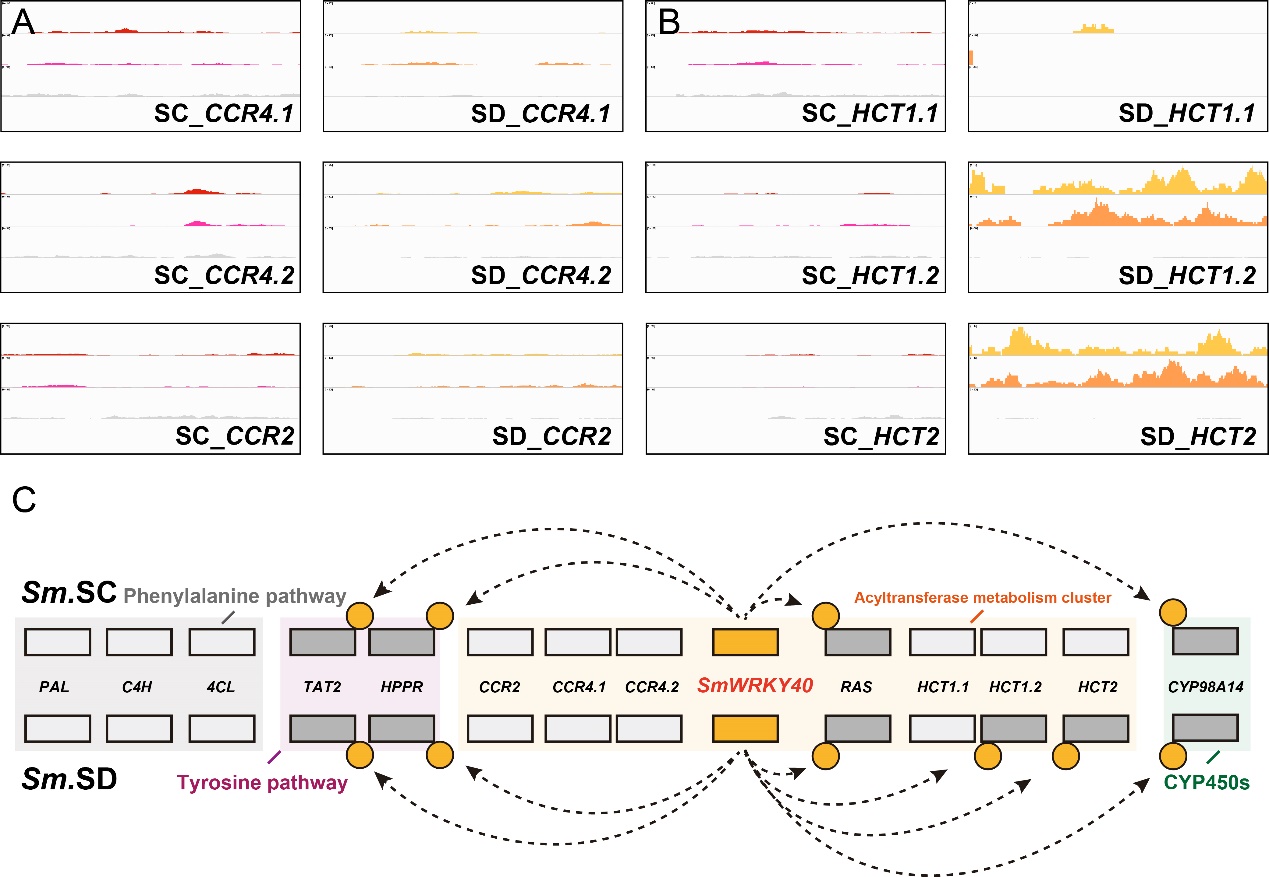


**Fig S13.** SmWRKY40 binding peaks on promoters of other pathway genes. **A.** SmWRKY40 binding peaks on promoters of *CCR4.1*, *CCR4.2*, *CCR2*, *HCT1.1*, *HCT1.2*, and *HCT2*. Red peaks represent the *Sm.*SC promoters, yellow peaks represent the *Sm.*SD promoters, and gray peaks represent Input. **B.** Overview map of SmWRKY40 binding patterns to the promoters of phenolic acid metabolism pathway genes of *Sm*.SD and *Sm*.SD.


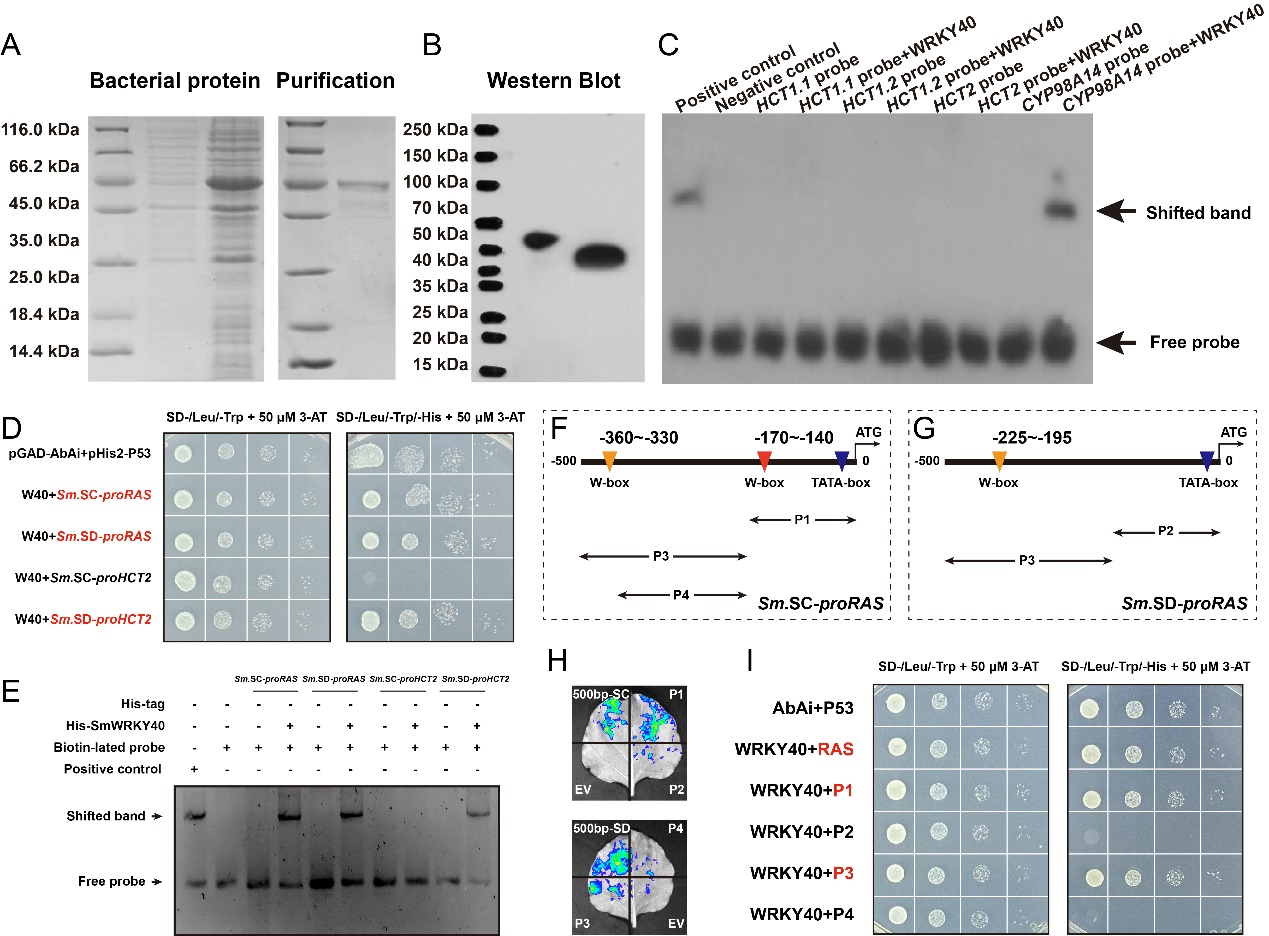


**Fig S14.** Comparison of the binding of WRKY40 transcription factors to the promoters of the *RAS* and *HCT2* genes. **A.** Protein induction and affinity purification. **B.** Western Blot. **C.** Protein binding regions of other gene promoter in Electrophoretic Mobility Shift Assays (EMSA). **D.** We cloned the full-length promoters of the *RAS* and *HCT2* genes of Sm.SC and Sm.SD (2000 bp upstream of ATG) into the pHis2 vector and co transfected them with the pGADT7-*WRKY40* recombinant plasmid into Y187 yeast. Cultivate in nutrient deficient medium to verify the interaction between transcription factors and DNA. **E.** We labeled the full-length promoters of *RAS* and *HCT2* genes in *Sm.*SC and *Sm.*SD, and incubated them with WRKY40-6×His recombinant protein for Electrophoretic Mobility Shift Assay. **F-G.** We predicted the distribution of W-box elements in the *HCT2* gene promoter regions of *Sm.*SC and *Sm.*SD. **H-I.** Daul LUC and Y1H experiments with truncated promoter.


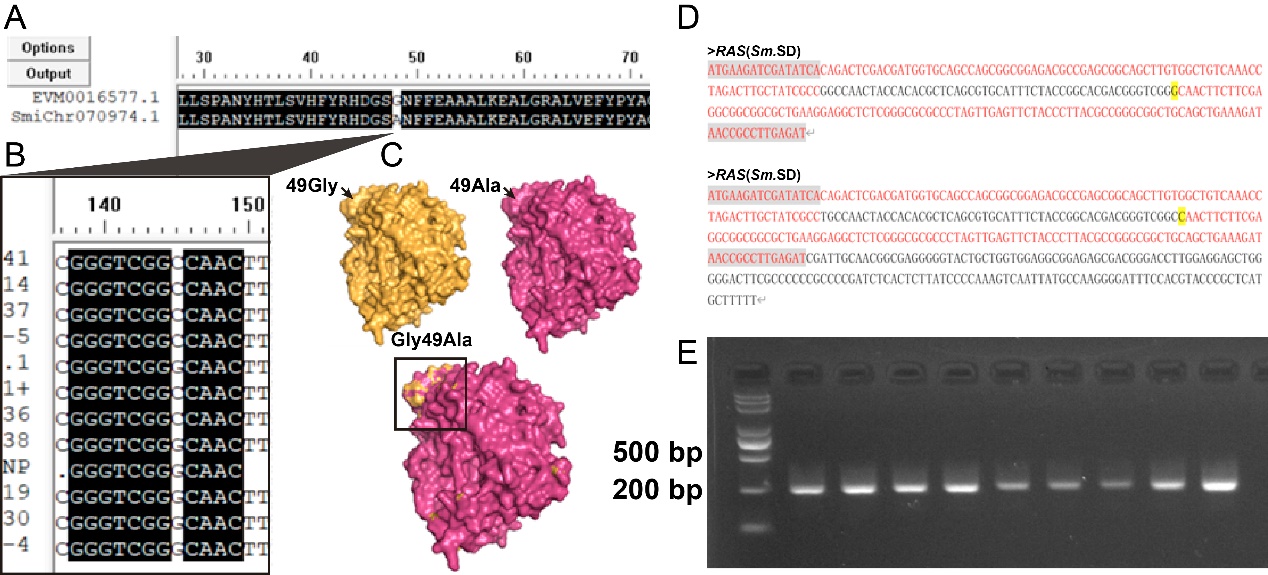


**Fig S15.** Haplotype comparison of rosmarinic acid synthase (RAS) from *Sm.*SC and *Sm.*SD. **A.** Amino acid multiple sequence alignment of RAS from *Sm.*SC and *Sm.*SD. **B.** Nucleotide multiple sequence alignment of RAS from *Sm.*SC and *Sm.*SD. **C.** Comparison of protein 3 D structures of RAS from *Sm.*SC (pink) and *Sm.*SD (yellow). **D.** Cloning primers were designed for the exons where the SNP is located. **E.** Mixed DNA extraction for representative populations and cloning of the exon where the SNP is located.


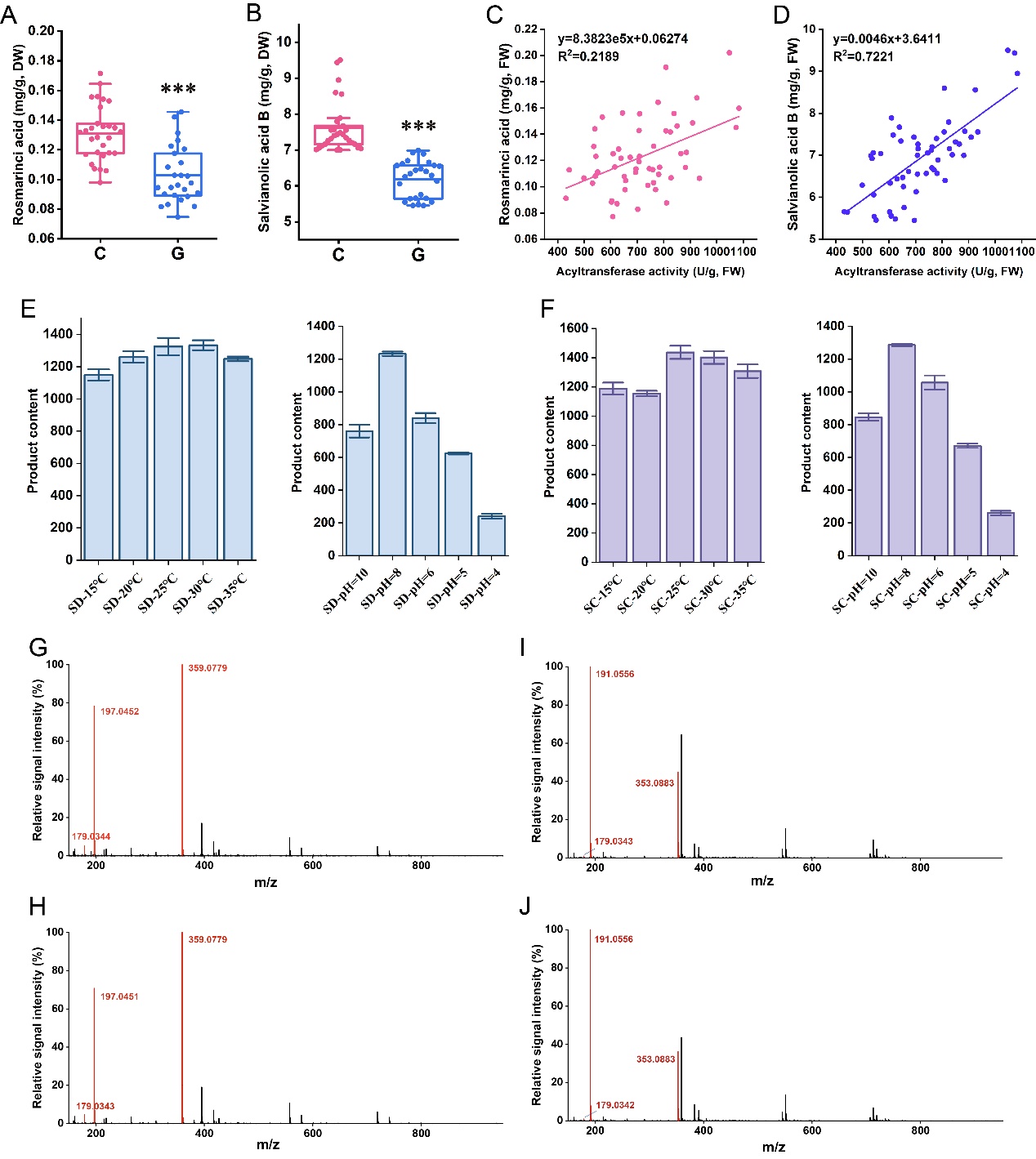


**Fig S16.** Association analysis of RA and SAB content with haplotypes and acyltransferase activity in population samples, and groping of reaction conditions of rosmarinic acid synthase (RAS) from *Sm.*SC and *Sm.*SD. High-resolution mass spectrometry data from the enzymatic reaction system are supplemented **A-B.** Statistics of rosmarinci acid and salvianolic acid B content in the roots of two RAS haplotypes in natural populations. C-D. Statistics of association between acyltransferase activity and rosmarinci acid and salvianolic acid B content in natural populations. **E-F.** Enzymatic reaction efficiencies under different pH and temperature conditions. **G-H.** MS spectra of the reaction system for the polymerization of salvianolic acid and caffeoyl CoA catalyzed by ^149C^RAS (G) and ^149G^RAS (H) to form rosmarinic acid. **I-J.** MS spectra of the reaction system for the polymerization of quinic acid and caffeoyl CoA catalyzed by ^149C^RAS (I) and ^149G^RAS (J) to form caffeoyl quinic acid.


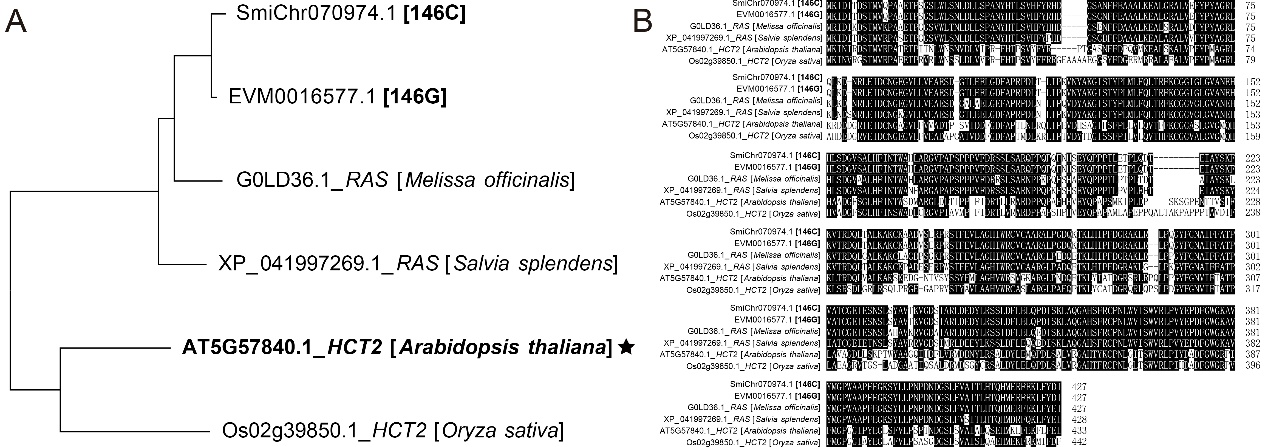


**Fig S17.** Phylogeny and multiple sequence alignment of *SmRAS* and homologous sequences. **A.** The phylogenetic tree based on the NJ model constructed through MEGA7 has the optimal topology generated after 1,000 bootstrap iterations. **B.** Conservative motifs (black modules) between species were identified through multiple sequence alignment of six representative amino acid sequences.


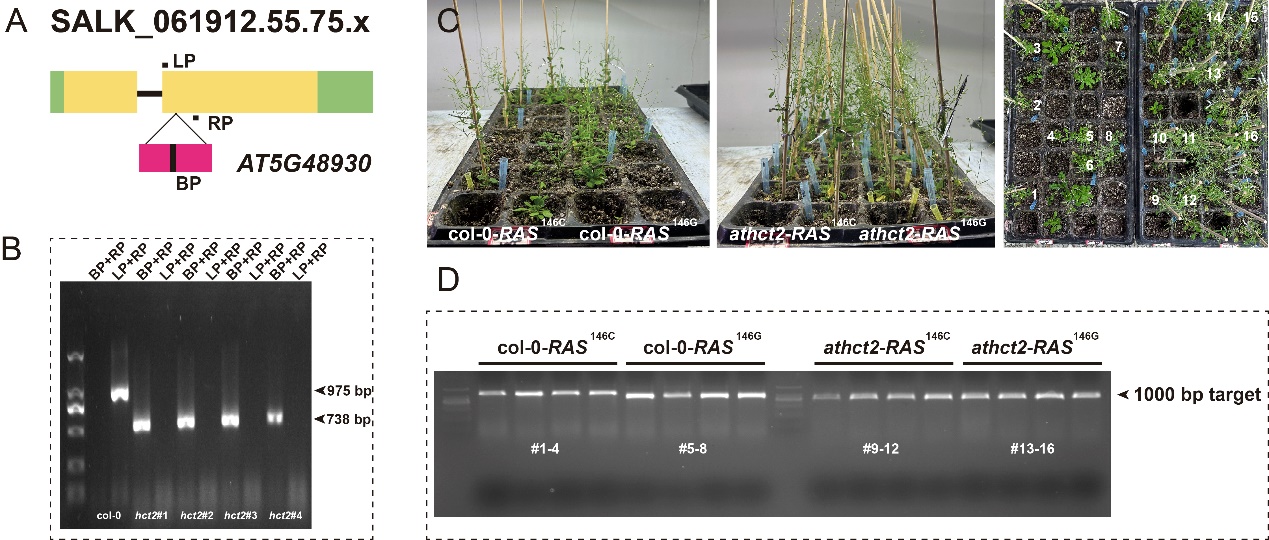


**Fig S18.** Identification of *Arabidopsis* mutants and transgenic plants. **A.** Schematic representation of the structure of the T-DNA insertion mutants. **B.** Triple-primer method PCR identification of T-DNA insertion mutants. C. Comparison of phenotypes between transgenic and mutant *Arabidopsis*. **D.** PCR Identification of *SmRAS*^146C^ and *SmRAS*^146G^ overexpressing *Arabidopsis*.


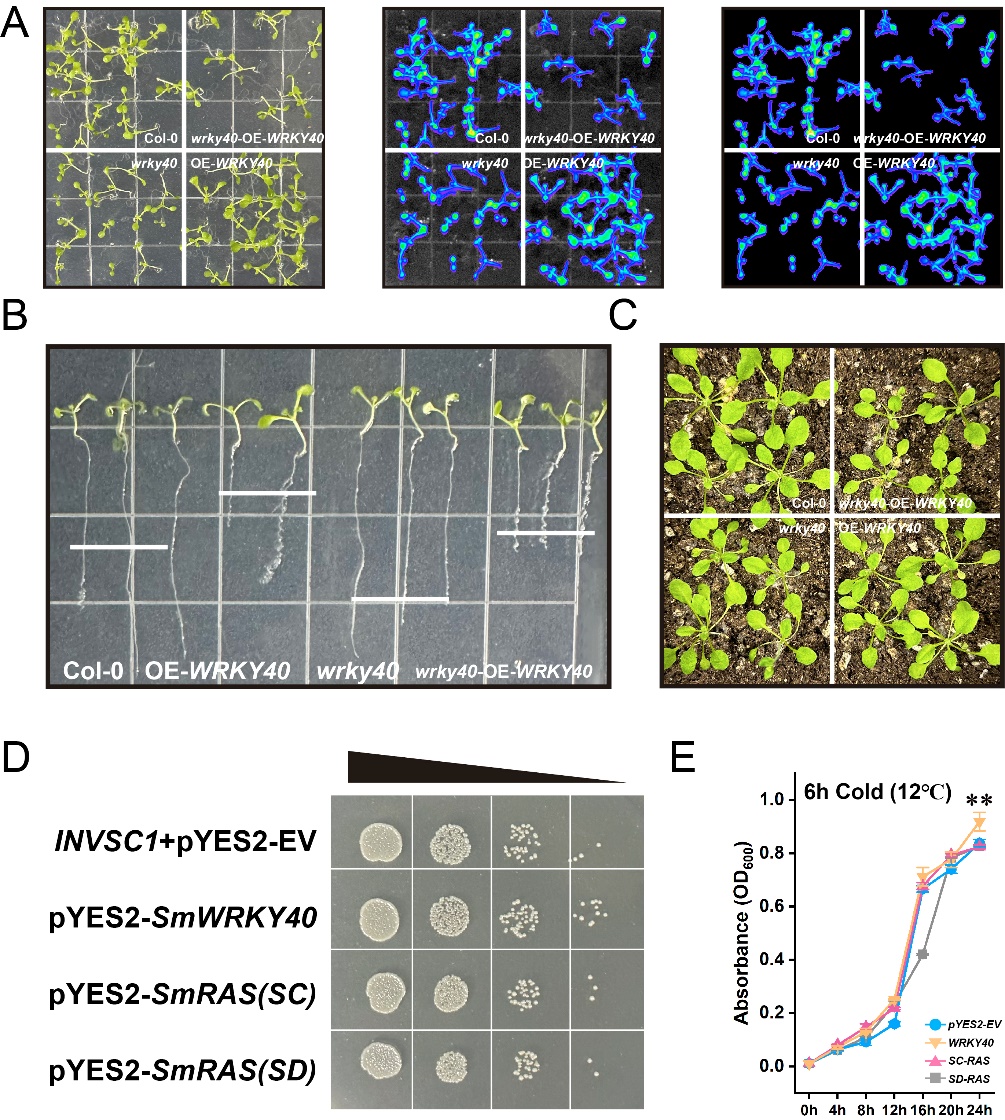


**Fig S19.** Heterologous expression validates the biological functions of *SmWRKY40* and *SmRASs* in response to cold stress. **A.** Plate growth phenotype of wild-type (col-0), OE-*SmWRKY40*, *atwrky40* mutant, and *SmWRKY40* complemented *atwrky40* mutant *A. thaliana* under cold stress. The fluorescence intensity reflects the chlorophyll fluorescence in the plant leaves, and the stronger the fluorescence intensity represents the higher the plant biological activity. **B.** Root length of different genotypes of *Arabidopsis* under cold stress. **C.** Phenotypes of different genotypes of *Arabidopsis* under cold stress. **D.** Growth status of yeast transferred with *pYES2* vector, *pYES2*-*SmWRKY40*, *pYES2*-*RAS*^146C^ and *pYES2*-*RAS*^146G^ on plates under cold stress.


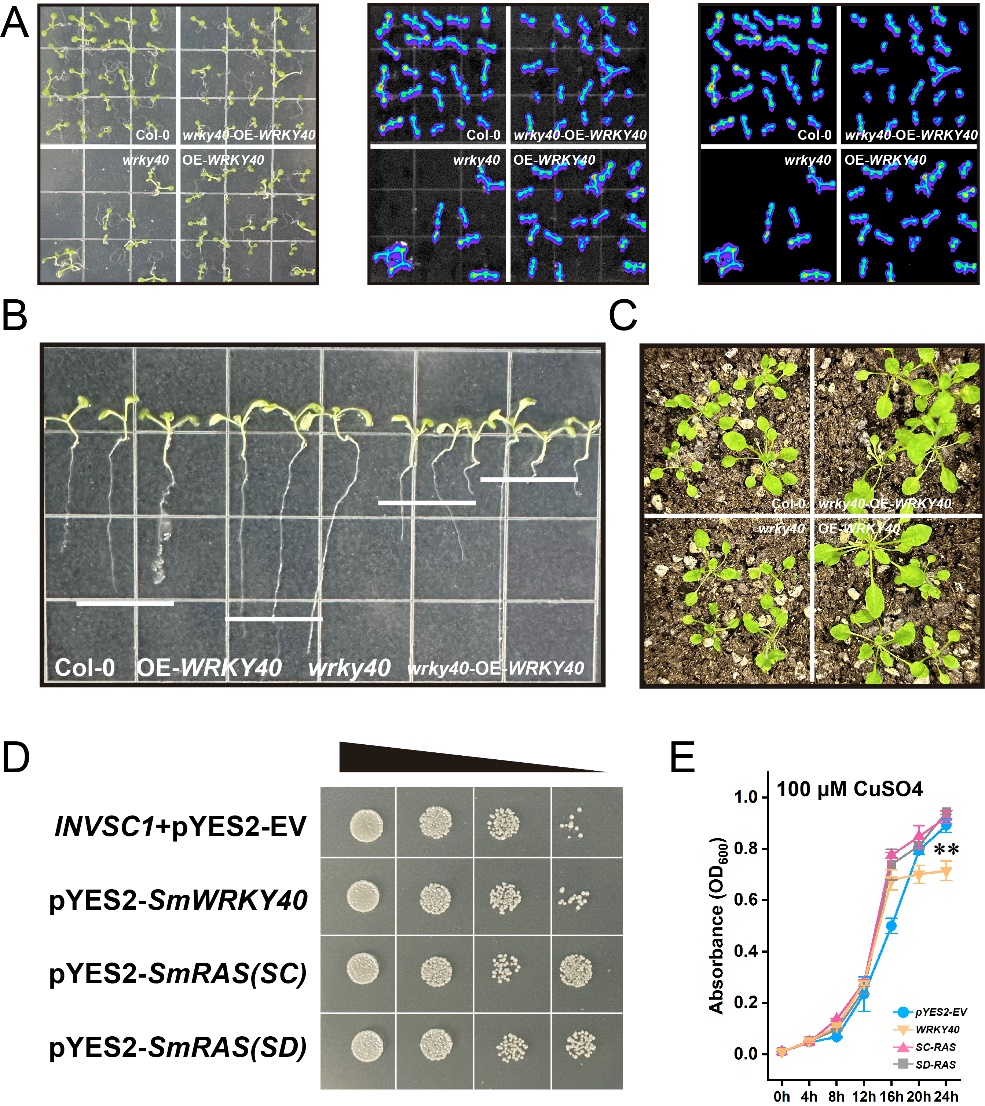


**Fig S20.** Heterologous expression validates the biological functions of *SmWRKY40* and *SmRASs* in response to Cu^2+^ stress. **A.** Plate growth phenotype of wild-type (col-0), OE-*SmWRKY40*, *atwrky40* mutant, and *SmWRKY40* complemented *atwrky40* mutant *A. thaliana* under Cu^2+^ stress. The fluorescence intensity reflects the chlorophyll fluorescence in the plant leaves, and the stronger the fluorescence intensity represents the higher the plant biological activity. **B.** Root length of different genotypes of *Arabidopsis* under Cu^2+^ stress. **C.** Phenotypes of different genotypes of *Arabidopsis* under Cu^2+^ stress. **D.** Growth status of yeast transferred with *pYES2* vector, *pYES2*-*SmWRKY40*, *pYES2*-*RAS*^146C^ and *pYES2*-*RAS*^146G^ on plates under cold stress.

**Supplementary tables**

**Table S1.** Basic information on germplasm resources of *S. miltiorrhiza* with different ecotypes.

**Table S2.** Investigation of the meteorological environment in different *S. miltiorrhiza* production regions.

**Table S3.** Comparative metabolomic analysis of *Sm.*SC and *Sm.*SD.

**Table S4.** Statistics of the whole-genome sequencing data based on the PacBio platform.

**Table S5.** Hi-C assisted assembly data statistics.

**Table S6.** Genomic orthologous identification of *S. miltiorrhiza* and its related species.

**Table S7.** Comparative transcriptomics analysis of *Sm.*SC and *Sm.*SD.

**Table S8.** Comparative analysis of key enzyme genes and metabolites in the phenolic acid metabolic pathway in *Sm.*SC and *Sm.*SD.

**Table S9.** Gene-metabolite regulatory network of phenolic acid metabolism pathway constructed based on integrative omics analysis.

**Table S10.** Data statistics for population resequencing of *Sm.*SC and *Sm.*SD.

**Table S11.** Single nucleotide polymorphism (SNP) statistics on HIC_ASM_6 in *Sm.*SC and *Sm.*SD population resequencing.

**Table S12.** Statistics of large segment variation (Indel) located on HIC_ASM_6 in *Sm.*SC and *Sm.*SD population resequencing.

**Table S13.** Population differentiation (*F*st) statistics of *Sm.*SC and *Sm.*SD on HIC_ASM_6.

**Table S14.** Statistics of the peaks of interaction with SmWRKY40 in phenolic acid metabolism clusters of *Sm.*SC and *Sm.*SD based on DAP-seq.
